# Supplementary material for: Metabolic diversity within the globally abundant Marine Group II Euryarchaea offers insight into ecological patterns
Source: Nat Commun. 2019 Jan 17;10:271. doi: 10.1038/s41467-018-07840-4 (PMC6336850; doi:10.1038/s41467-018-07840-4)
Supplement: Supplementary file 1 — Supplementary Information [file 41467_2018_7840_MOESM1_ESM.pdf]

Metabolic Diversity within the Globally Abundant Marine Group II Euryarchaea Offers Insight into Ecological Patterns

Benjamin J Tully[1,2]

1. Department of Biological Sciences, University of Southern California, Los Angeles, CA, USA

2. Center for Dark Energy Biosphere Investigations, University of Southern California, Los Angeles, CA, USA

Corresponding author: Benjamin J Tully (e-mail: btully@usc.edu; tully.bj@gmail.com)

Supplementary Information

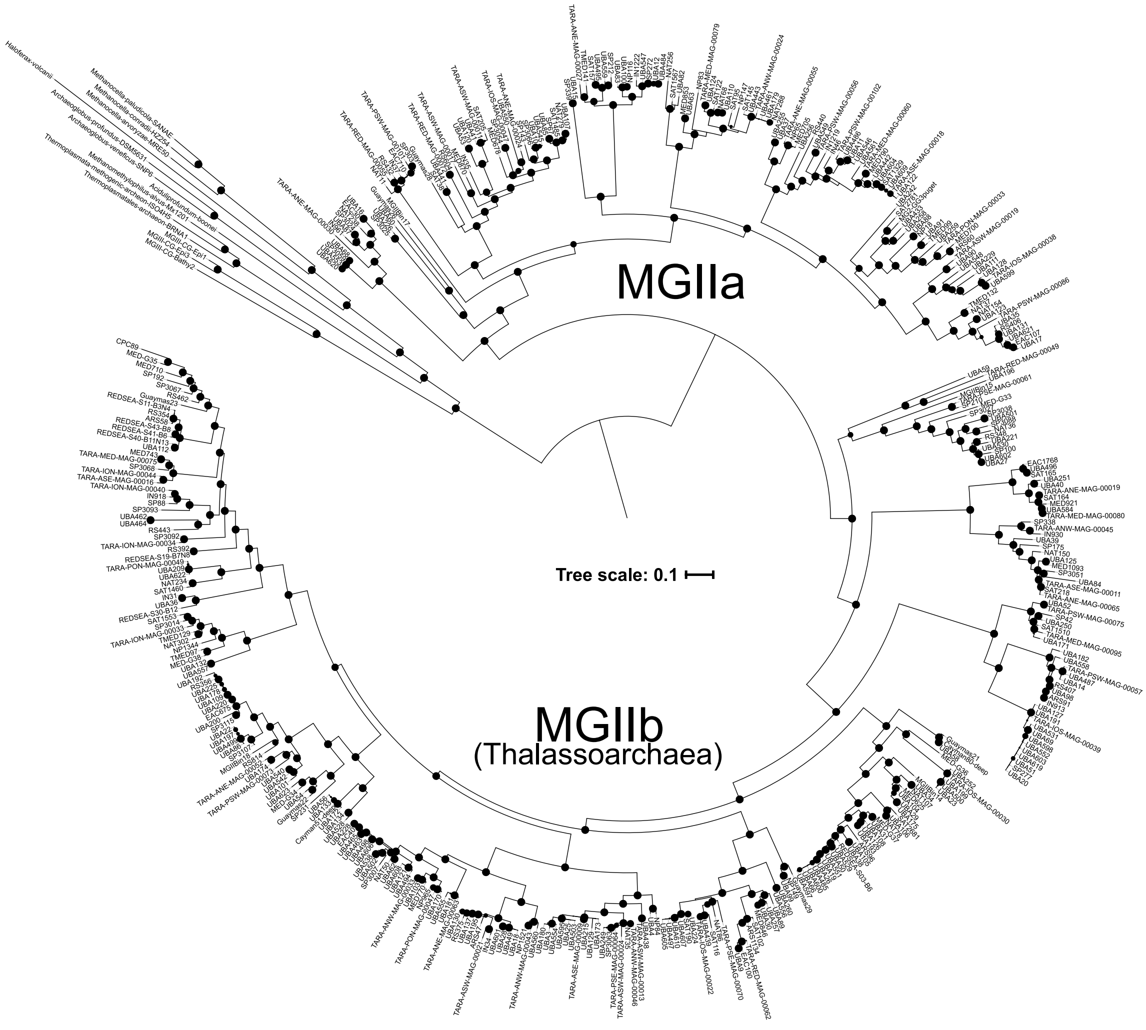

Supplementary Figure 1. A phylogenomic tree constructed using 120 concatenated marker proteins for all of the MGII. Bootstrap values are scaled proportionally between 0.75-1. Source data are provided as a Source Data file.

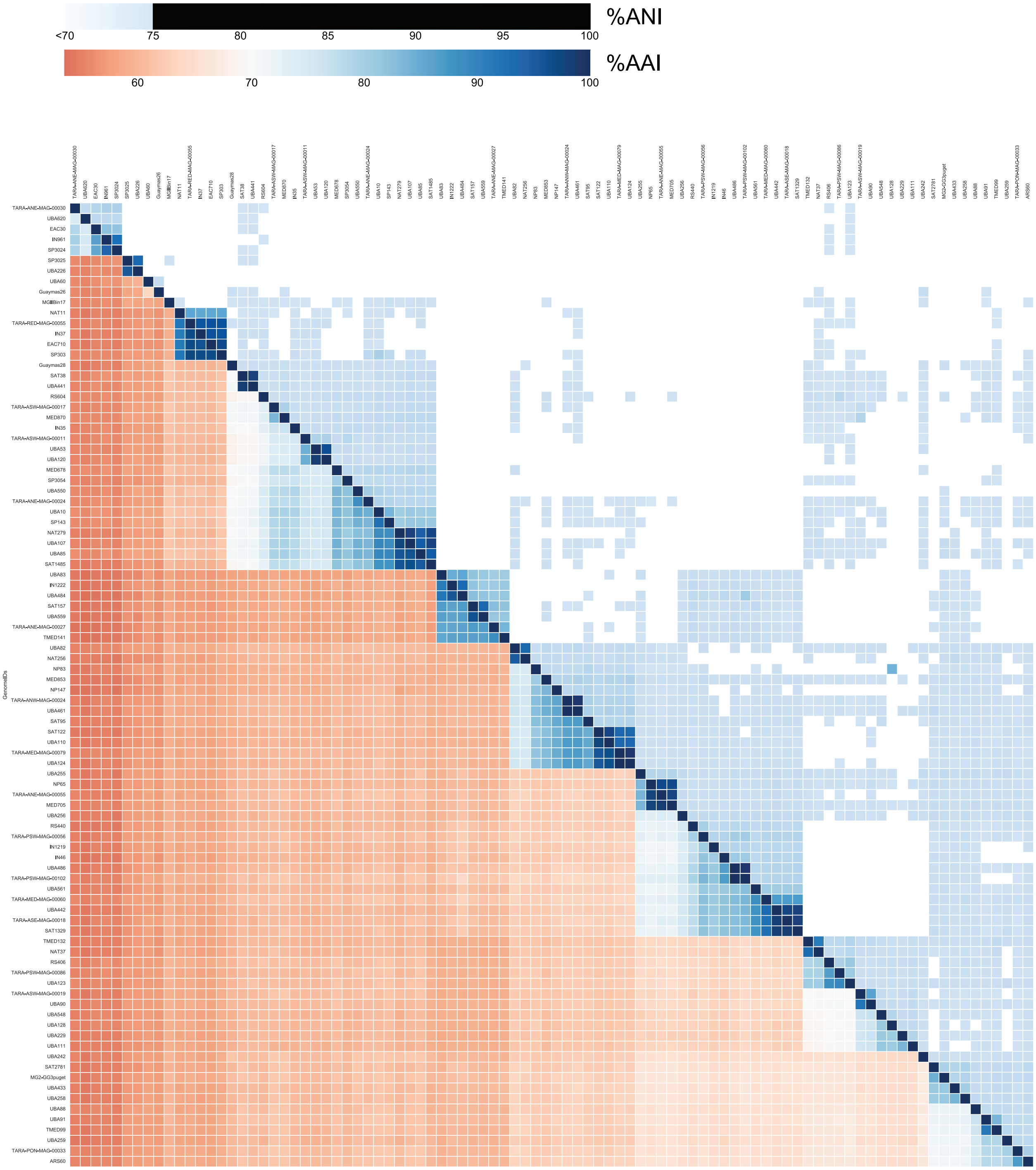

Supplementary Figure 2. Heatmap of pairwise AAI and ANI for the MGIIa. Genomes are ordered based on their placement in the phylogenetic tree in Figure 1. Source data are provided as a Source Data file.

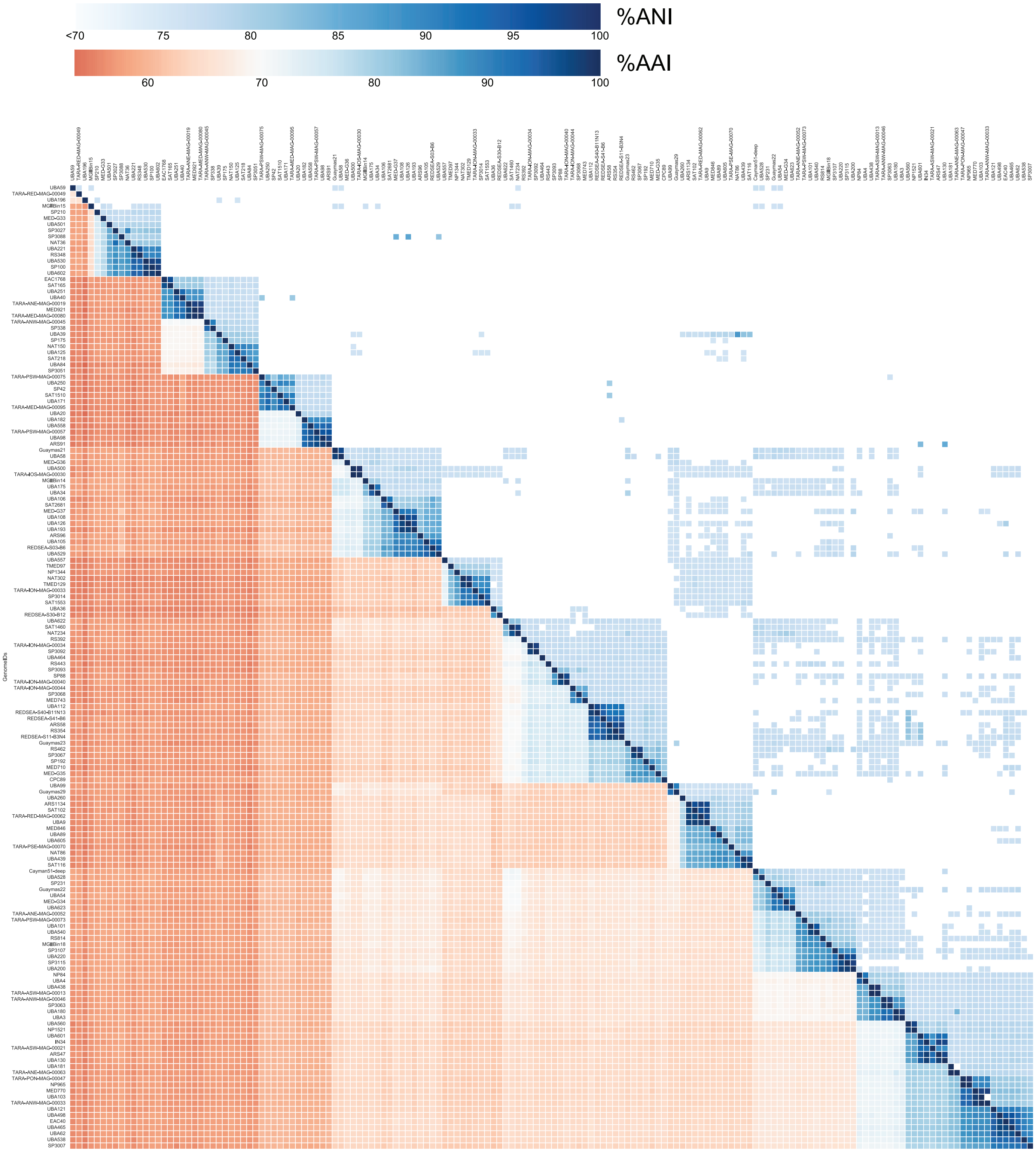

Supplementary Figure 3. Heatmap of pairwise AAI and ANI for the MGIIb. Genomes are ordered based on their placement in the phylogenetic tree in Figure 1. Source data are provided as a Source Data file.

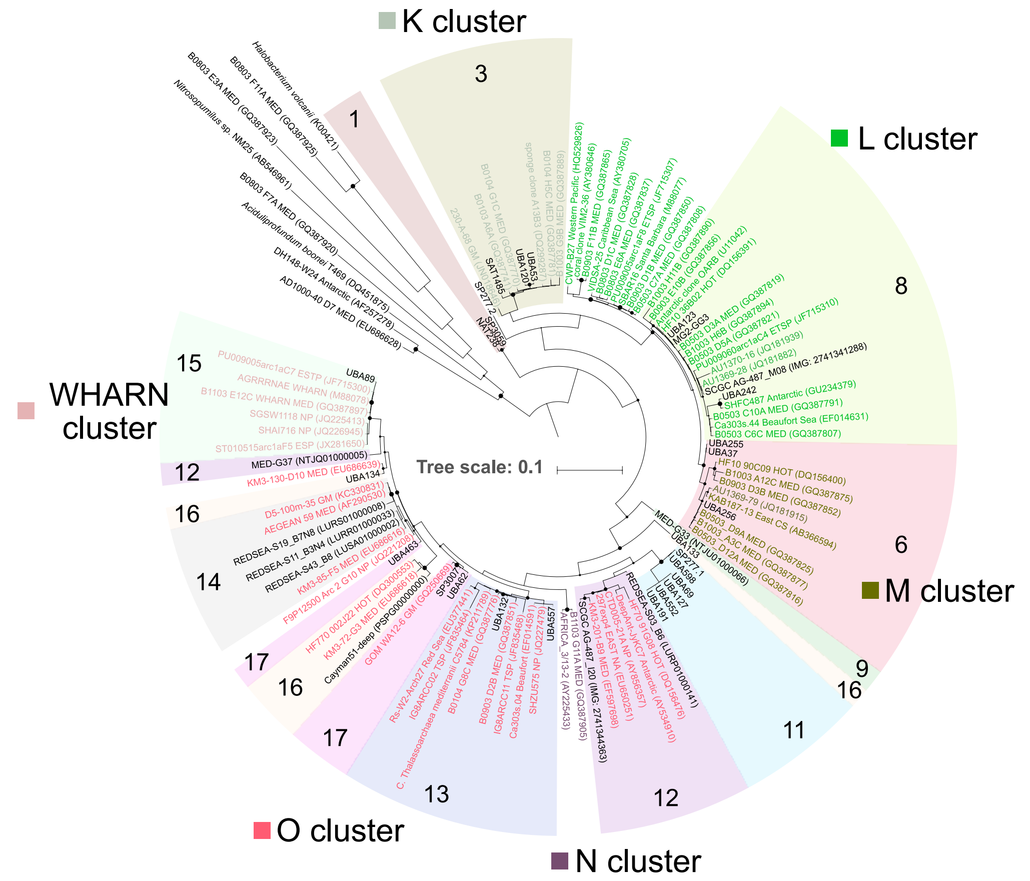

Supplementary Figure 4. A phylogenetic tree of the 16S rRNA gene for 35 MGII genomes combined with previously defined reference sequences. Previously observed clusters (leaves are colored to match corresponding clusters) that could be linked to newly defined subclades based on the occurrence of genome linked 16S rRNA sequences are shown. Internal nodes within large clusters (*e.g.*, O cluster) are used to demarcate different families where appropriate. Source data are provided as a Source Data file.

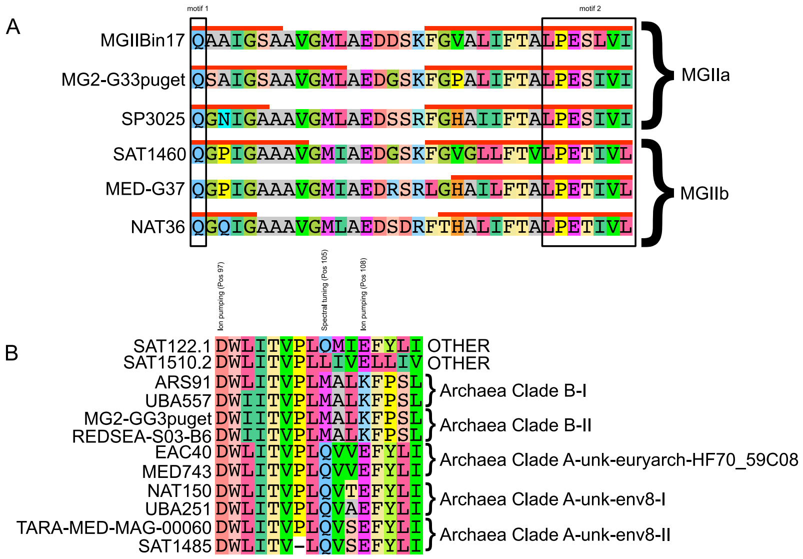

Supplementary Figure 5. A) Alignment *c* ring protein subunit (AtpK) for a selection of genomes. Transmembrane helices predicted using the TMHMM server (v.2.0) are denoted as red lines above the predicted region. Black boxes highlight the two conserved motifs that have been previously identified in Na^+ translocating ATP synthases. B) Alignment of the region used to predict functionality and spectral tuning amongst rhodopsins for a selection of genomes. Group assignments are based on clusters in Supplementary Figure 6. “OTHER” refers to rhodopsin sequences that did not fall within Archaea Clade-A or -B.

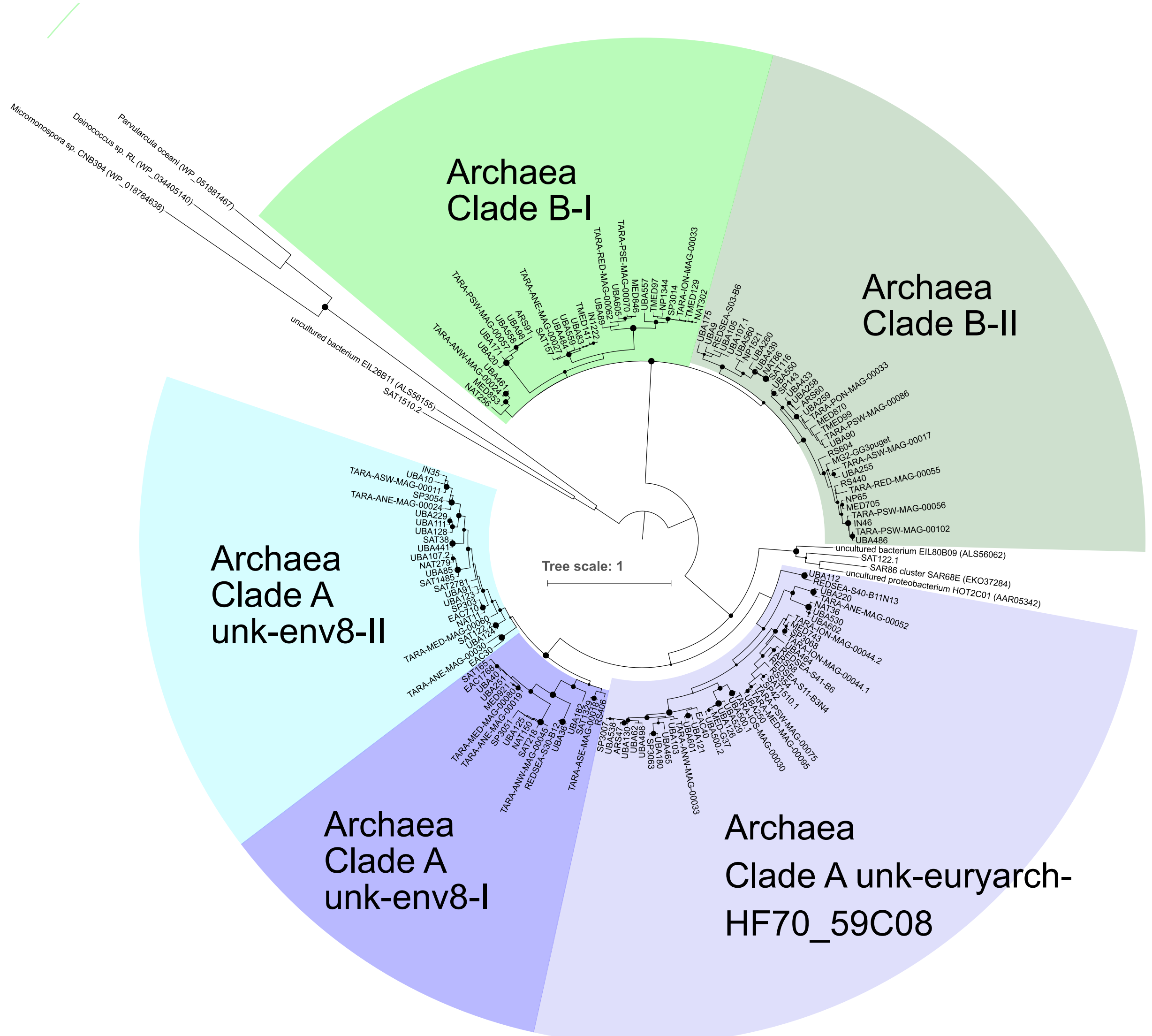

Supplementary Figure 6. Phylogenetic tree of the rhodopsin predicted proteins (226AA alignment) identified in the MGII genomes. Subclades in previously established proteorhodopsin clades are denoted. Bootstrap values are scaled proportionally between 0.75-1. Source data are provided as a Source Data file.

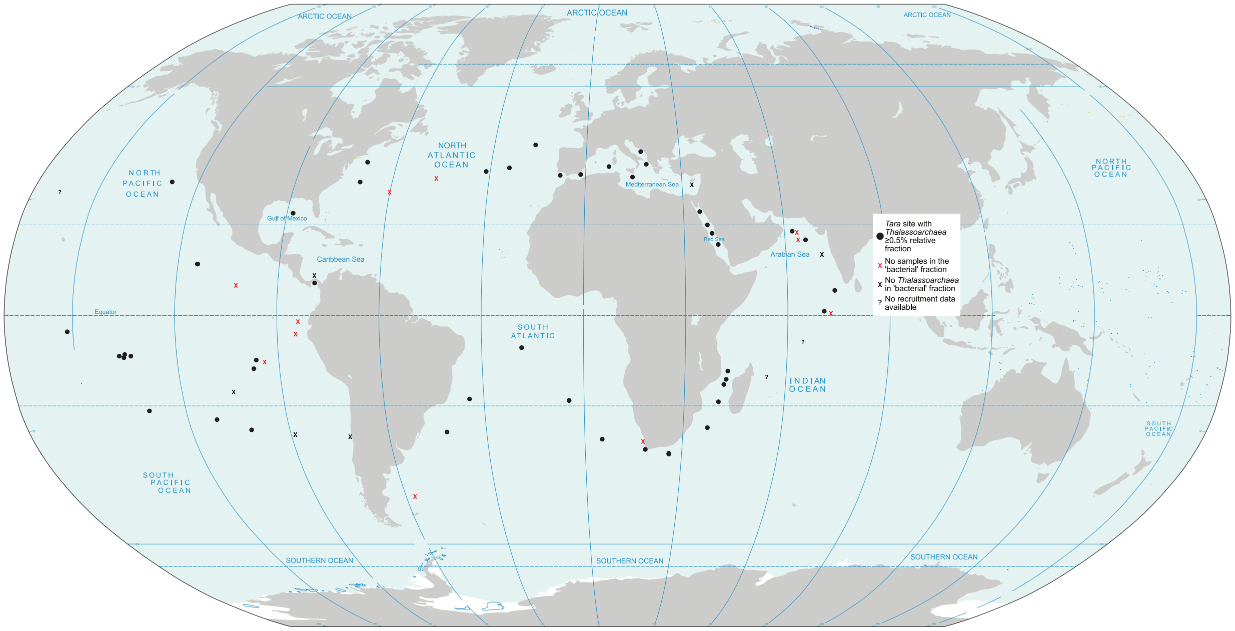

Supplementary Figure 7. Global map detailing the locations of the *Tara* Oceans sampling stations. Stations where at least one metagenomic sample recruited ≥0.5% relative fraction to the thalassoarchaeal genomes are represented as black dots. Stations lacking metagenomic samples in the 'bacterial' size fraction (0.22-3.0µm) are denoted as a red 'X'. Stations with metagenomic samples in the 'bacterial' size fraction but did not recruit ≥0.5% relative fraction are denoted as a black 'X'. Three stations (TARA048, -052, and -132) were not included in this analysis are denoted as question marks. The map in Supplementary Figure 7 was modified under a CC BY-SA 3.0 license from 'Oceans and Seas boundaries map' by Pinpin. The original file and accompanying license can be found here: https://commons.wikimedia.org/wiki/File:Oceans_and_seas_boundaries_map-en.svg.

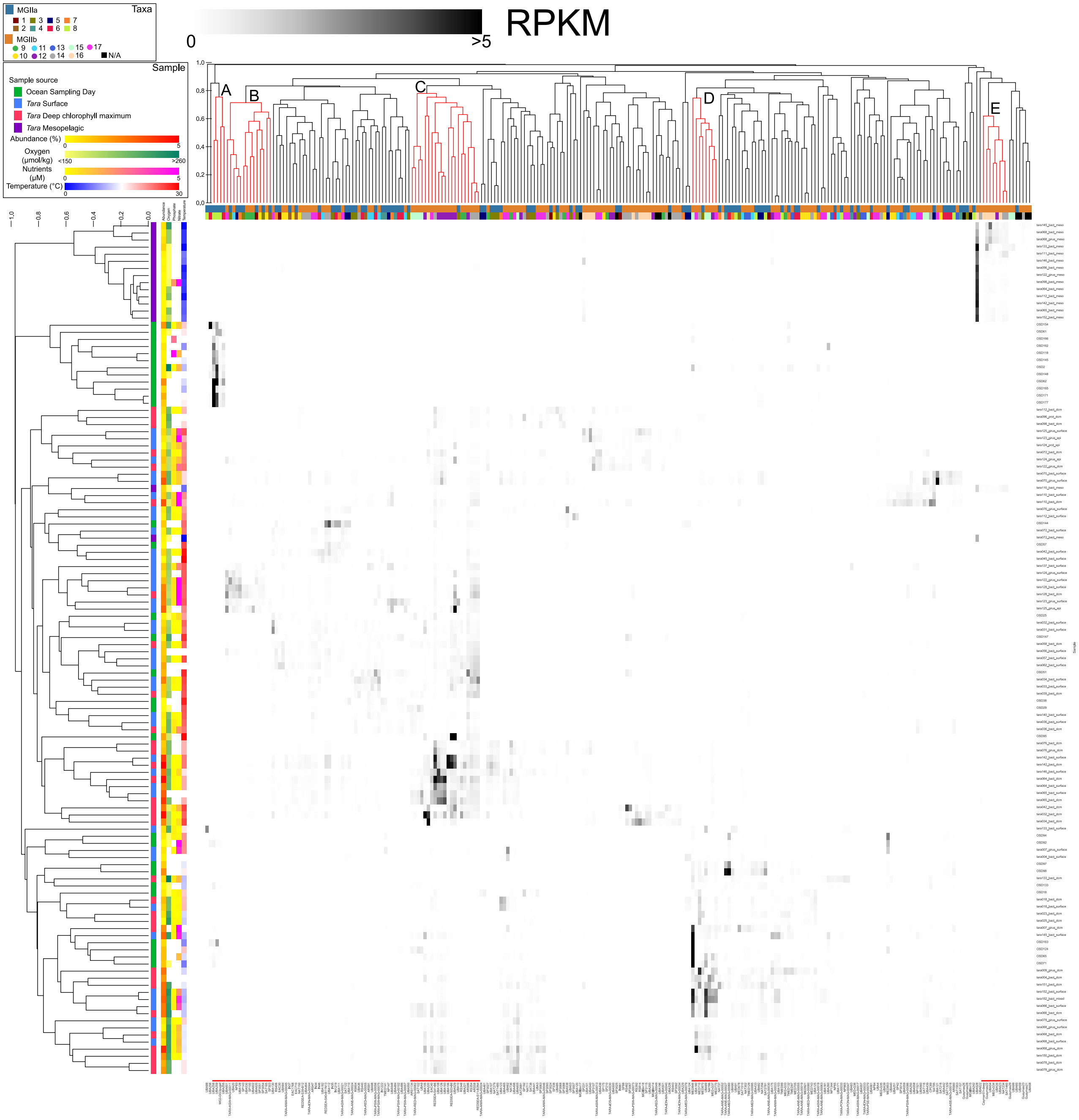

Supplementary Figure 8. A heatmap displaying the RPKM values for all of the MGII genomes in high abundance samples (≥0.5% relative fraction). RPKM values are scaled from 0-5 with values ≥5 in black (median, 0.0001; maximum, 9.25). Samples are average linkage hierarchically clustered based on a Bray-Curtis dissimilarity distance for all MGII RPKM values and the sample source is displayed. The available environmental parameters are presented as colored heatmaps (missing parameters are not displayed). The order of the sample hierarchical clustering and displayed environmental parameters are the same as those presented in Figure 5. Source data are provided as a Source Data file.

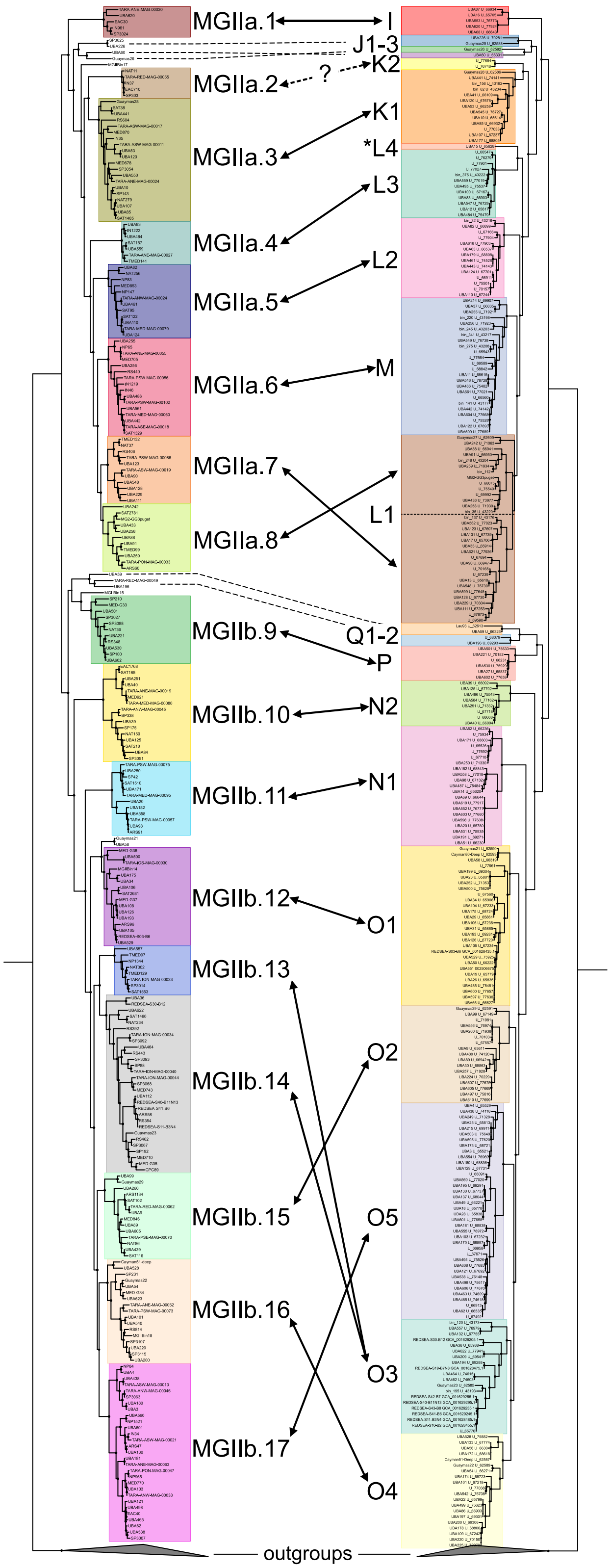

Supplementary Figure 9. Comparison of the MGII phylogenetic tree between this manuscript and Rinke *et al.* (2018). The phylogenetic tree from this manuscript is identical to Figure 1 (n = 258). The Rinke *et al.* phylogenetic tree was constructed using the 270 genomes in the manuscript with

the same methodology described here (HMM search for 120 markers, alignment with MUSCLE, automated trimming, concatenation, and phylogenetic tree with FastTree2). The outgroup is identical for both trees. Bootstrap values are scaled proportionally between 0.75-1.

Supplementary Tables

Supplementary Table 1. Information for all genomes used in study, including source ID numbers, clade assignment, completion stats (length, percent complete, percent contamination, percent strain heterogeneity), and source reference. Estimated completeness (%Comp); estimated contamination (%Contam); estimated strain heterogeneity (%Strain); Size is displayed in Mbp.

Name	Summarized ID	MGII Clade	Source	Accession ID	Size	16S	%Comp	%Contam	%Strain	Reference
Marine group II.B Euryarchaeota SCGC AG-487_I20	AG-487-I20	MGIIb	IMG	2740891966	0.78	yes	42.93	0.00	0.00	GOLD Study ID Gs110190.
Marine group II.A Euryarchaeota archaeon SCGC AG-487_M08	AG-487-M08	MGIIa	IMG	2740891962	0.81	yes	40.00	0.00	0.00	GOLD Study ID Gs110190.
ARS1134	ARS1134	MGIIb	NCBI	NZDA00000000	1.36	no	73.14	0.80	50.00	Tully, B. J. et al., 2018
ARS47	ARS47	MGIIb	NCBI	NYZV00000000	1.17	no	58.40	0.00	0.00	Tully, B. J. et al., 2018
ARS58	ARS58	MGIIb	NCBI	NYZK00000000	1.08	no	65.82	1.44	20.00	Tully, B. J. et al., 2018
ARS60	ARS60	MGIIa	NCBI	NYZH00000000	2.20	no	82.80	1.60	0.00	Tully, B. J. et al., 2018
ARS91	ARS91	MGIIb	NCBI	NYXY00000000	1.31	no	66.40	1.60	0.00	Tully, B. J. et al., 2018
ARS96	ARS96	MGIIb	NCBI	NYXO00000000	1.62	no	61.73	0.55	0.00	Tully, B. J. et al., 2018
Cayman51	Cayman51-deep	MGIIb	NCBI	PSPG00000000	1.48	yes	84.67	0.00	0.00	Li, M. et al., 2015
Cayman80	Cayman80-deep	MGIIb	NCBI	PSPC00000000	1.32	no	68.76	0.80	0.00	Li, M. et al., 2015
CPC89	CPC89	MGIIb	NCBI	NYST00000000	0.88	no	53.24	0.00	0.00	Tully, B. J. et al., 2018
EAC100	EAC100	MGIIb	NCBI	NZTY00000000	1.29	no	68.27	1.60	100.00	Tully, B. J. et al., 2018
EAC107	EAC107	MGIIa	NCBI	NZTR00000000	1.93	no	80.13	2.67	100.00	Tully, B. J. et al., 2018
EAC1768	EAC1768	MGIIb	NCBI	NZSV00000000	1.84	no	72.67	4.00	50.00	Tully, B. J. et al., 2018
EAC30	EAC30	MGIIa	NCBI	NZRR00000000	1.71	no	77.33	0.80	100.00	Tully, B. J. et al., 2018
EAC40	EAC40	MGIIb	NCBI	NZRH00000000	1.32	no	69.16	1.07	50.00	Tully, B. J. et al., 2018
EAC675	EAC675	MGIIb	NCBI	NZOX00000000	1.23	no	62.74	4.84	87.50	Tully, B. J. et al., 2018
EAC710	EAC710	MGIIa	NCBI	NZOE00000000	1.83	no	76.80	2.40	33.33	Tully, B. J. et al., 2018
Guaymas21	Guaymas21	MGIIb	Personal comm	Personal comm	1.50	no	80.00	2.40	33.33	Li, M. et al., 2015
Guaymas22	Guaymas22	MGIIb	Personal comm	Personal comm	1.45	no	81.56	0.64	25.00	Li, M. et al., 2015
Guaymas23	Guaymas23	MGIIb	Personal comm	Personal comm	1.63	no	86.00	2.40	0.00	Li, M. et al., 2015
Guaymas26	Guaymas26	MGIIa	Personal comm	Personal comm	1.75	no	77.73	0.80	0.00	Li, M. et al., 2015
Guaymas28	Guaymas28	MGIIa	Personal comm	Personal comm	1.92	no	85.33	2.86	0.00	Li, M. et al., 2015
Guaymas29	Guaymas29	MGIIb	Personal comm	Personal comm	1.60	no	78.76	2.78	83.33	Tully, B. J. et al., 2018
IN1219	IN1219	MGIIa	NCBI	NZMN00000000	1.60	no	67.73	0.00	0.00	Tully, B. J. et al., 2018
IN1222	IN1222	MGIIa	NCBI	NZMM00000000	1.71	no	69.73	2.40	66.67	Tully, B. J. et al., 2018
IN31	IN31	MGIIb	NCBI	NZLQ00000000	1.06	no	51.81	0.00	0.00	Tully, B. J. et al., 2018
IN34	IN34	MGIIb	NCBI	NZLN00000000	1.12	no	56.14	1.33	66.67	Tully, B. J. et al., 2018
IN35	IN35	MGIIa	NCBI	NZLM00000000	1.98	no	84.13	2.80	50.00	Tully, B. J. et al., 2018
IN37	IN37	MGIIa	NCBI	NZLK00000000	1.72	no	71.64	0.93	50.00	Tully, B. J. et al., 2018
IN46	IN46	MGIIa	NCBI	NZLA00000000	1.61	no	63.25	1.60	0.00	Tully, B. J. et al., 2018
IN913	IN913	MGIIb	ANVIO-REFINED	figshare	1.18	no	61.60	0.86	0.00	Tully, B. J. et al., 2018
IN918	IN918	MGIIb	ANVIO-REFINED	figshare	1.20	no	60.80	1.60	0.00	Tully, B. J. et al., 2018
IN930	IN930	MGIIb	NCBI	NZKI00000000	1.32	no	53.37	2.40	0.00	Tully, B. J. et al., 2018
IN961	IN961	MGIIa	NCBI	NZKH00000000	1.50	no	72.40	0.80	100.00	Tully, B. J. et al., 2018
Marine Group II euryarchaeote MED-G33	MED-G33	MGIIb	NCBI	NTJU00000000	1.27	yes	57.26	0.00	0.00	Haro-Moreno, J. M. *et al.,* 2018
Marine Group II euryarchaeote MED-G34	MED-G34	MGIIb	NCBI	NTJT00000000	1.03	no	59.35	0.00	0.00	Haro-Moreno, J. M. *et al.,* 2018
Marine Group II euryarchaeote MED-G35	MED-G35	MGIIb	NCBI	NTJS00000000	1.20	no	65.39	0.00	0.00	Haro-Moreno, J. M. *et al.,* 2018
Marine Group II euryarchaeote MED-G36	MED-G36	MGIIb	NCBI	NTJR00000000	0.89	no	56.40	0.00	0.00	Haro-Moreno, J. M. *et al.,* 2018
Marine Group II euryarchaeote MED-G37	MED-G37	MGIIb	NCBI	NTJQ00000000	1.28	yes	71.73	0.00	0.00	Haro-Moreno, J. M. *et al.,* 2018
Marine Group II euryarchaeote MED-G38	MED-G38	MGIIb	NCBI	NTJP00000000	1.37	no	73.93	0.00	0.00	Haro-Moreno, J. M. *et al.,* 2018
MED1093	MED1093	MGIIb	ANVIO-REFINED	figshare	1.84	no	76.93	3.47	100.00	Tully, B. J. et al., 2018
MED211	MED211	MGIIa	NCBI	NZJG00000000	1.18	no	52.00	3.20	25.00	Tully, B. J. et al., 2018
MED625	MED625	MGIIb	NCBI	NZIB00000000	1.44	no	62.67	2.00	0.00	Tully, B. J. et al., 2018
MED678	MED678	MGIIa	NCBI	NZGQ00000000	1.62	no	56.74	0.80	100.00	Tully, B. J. et al., 2018
MED700	MED700	MGIIa	NCBI	NZGA00000000	1.40	no	59.28	0.00	0.00	Tully, B. J. et al., 2018
MED705	MED705	MGIIa	NCBI	NZFV00000000	1.81	no	74.53	0.00	0.00	Tully, B. J. et al., 2018
MED710	MED710	MGIIb	figshare	figshare	1.04	no	59.74	0.00	0.00	Tully, B. J. et al., 2018
MED743	MED743	MGIIb	NCBI	NZEY00000000	1.94	no	82.93	4.00	0.00	Tully, B. J. et al., 2018
MED770	MED770	MGIIb	NCBI	PAGY00000000	1.22	no	64.76	1.87	33.33	Tully, B. J. et al., 2018
MED790	MED790	MGIIa	NCBI	PAGK00000000	1.64	no	67.82	3.07	80.00	Tully, B. J. et al., 2018
MED846	MED846	MGIIb	NCBI	PAET00000000	1.33	no	74.27	3.36	90.00	Tully, B. J. et al., 2018

MED853	MED853	MGIIa	ANVIO-REFINED	figshare	1.91	no	81.33	3.2	75.00	Tully, B. J. et al., 2018
MED870	MED870	MGIIa	NCBI	PAEE00000000	1.61	no	74.13	2.40	100.00	Tully, B. J. et al., 2018
MED875	MED875	MGIIb	NCBI	PADZ00000000	1.31	no	51.02	2.67	100.00	Tully, B. J. et al., 2018
MED921	MED921	MGIIb	ANVIO-REFINED	figshare	1.67	no	74.93	1.60	100.00	Tully, B. J. et al., 2018
MG2-GG3	MG2-GG3puget	MGIIa	NCBI	AHCG00000000	2.06	yes	83.20	0.00	0.00	Iverson, V. et al., 2012
Euryarchaeota MGII Bin14	MGIIBin14	MGIIb	IMG	2651870037	1.39	no	71.20	0.80	100.00	Thrash, J. C. *et al.,* 2017
Euryarchaeota MGII Bin15	MGIIBin15	MGIIb	IMG	2651870038	1.89	yes	83.20	1.60	0.00	Thrash, J. C. *et al.,* 2017
Euryarchaeota MGII Bin17	MGIIBin17	MGIIa	IMG	2651870039	1.80	yes	81.90	0.10	50.00	Thrash, J. C. *et al.,* 2017
Euryarchaeota MGII Bin18	MGIIBin18	MGIIb	IMG	2651870040	1.03	no	61.10	0.80	100.00	Thrash, J. C. *et al.,* 2017
NAT107	NAT107	MGIIb	NCBI	PABX00000000	1.26	no	66.32	1.20	50.00	Tully, B. J. et al., 2018
NAT11	NAT11	MGIIa	NCBI	PABV00000000	1.91	no	78.93	0.93	0.00	Tully, B. J. et al., 2018
NAT150	NAT150	MGIIb	NCBI	PADI00000000	1.59	no	63.64	1.60	100.00	Tully, B. J. et al., 2018
NAT154	NAT154	MGIIa	NCBI	PADF00000000	1.98	no	82.40	4.80	50.00	Tully, B. J. et al., 2018
NAT234	NAT234	MGIIb	NCBI	NZZZ00000000	1.36	no	65.69	4.00	50.00	Tully, B. J. et al., 2018
NAT238	NAT238	MGIIa	NCBI	NZZW00000000	1.89	yes	82.87	7.60	66.67	Tully, B. J. et al., 2018
NAT256	NAT256	MGIIa	NCBI	NZZD00000000	1.24	no	51.48	2.40	100.00	Tully, B. J. et al., 2018
NAT279	NAT279	MGIIa	NCBI	NZYG00000000	1.34	no	53.57	3.12	50.00	Tully, B. J. et al., 2018
NAT288	NAT288	MGIIa	NCBI	NZXZ00000000	1.67	no	76.53	0.00	0.00	Tully, B. J. et al., 2018
NAT302	NAT302	MGIIb	NCBI	NZXS00000000	1.41	no	60.35	3.73	100.00	Tully, B. J. et al., 2018
NAT35	NAT35	MGIIb	NCBI	NZXO00000000	1.05	no	55.82	1.60	75.00	Tully, B. J. et al., 2018
NAT36	NAT36	MGIIb	NCBI	NZXM00000000	1.39	no	58.45	1.26	33.33	Tully, B. J. et al., 2018
NAT37	NAT37	MGIIa	NCBI	NZXL00000000	1.60	no	65.01	0.80	100.00	Tully, B. J. et al., 2018
NAT50	NAT50	MGIIb	NCBI	NZWN00000000	0.97	no	58.36	1.60	50.00	Tully, B. J. et al., 2018
NAT68	NAT68	MGIIa	NCBI	NZVG00000000	2.09	no	82.53	3.20	75.00	Tully, B. J. et al., 2018
NAT78	NAT78	MGIIb	NCBI	NZUU00000000	1.13	no	52.27	0.80	100.00	Tully, B. J. et al., 2018
NAT86	NAT86	MGIIb	NCBI	NZUM00000000	1.08	no	54.33	1.60	0.00	Tully, B. J. et al., 2018
NP108	NP108	MGIIb	NCBI	PCCC00000000	1.06	no	55.76	0.80	0.00	Tully, B. J. et al., 2018
NP116	NP116	MGIIa	NCBI	PCBQ00000000	1.46	no	65.60	0.84	100.00	Tully, B. J. et al., 2018
NP1344	NP1344	MGIIb	NCBI	PCAI00000000	1.12	no	60.39	0.27	0.00	Tully, B. J. et al., 2018
NP147	NP147	MGIIa	NCBI	PBZN00000000	1.33	no	50.74	0.00	0.00	Tully, B. J. et al., 2018
NP149	NP149	MGIIb	NCBI	PBZK00000000	1.20	no	55.96	2.80	60.00	Tully, B. J. et al., 2018
NP1521	NP1521	MGIIb	NCBI	PBZE00000000	1.28	no	75.58	0.80	0.00	Tully, B. J. et al., 2018
NP18	NP18	MGIIa	NCBI	PBYY00000000	1.99	no	75.33	0.00	0.00	Tully, B. J. et al., 2018
NP65	NP65	MGIIa	NCBI	PBTH00000000	1.73	no	79.40	0.00	0.00	Tully, B. J. et al., 2018
NP83	NP83	MGIIa	NCBI	PBSP00000000	1.48	no	60.83	0.13	100.00	Tully, B. J. et al., 2018
NP84	NP84	MGIIb	NCBI	PBSO00000000	1.24	no	69.20	0.00	0.00	Tully, B. J. et al., 2018
NP965	NP965	MGIIb	NCBI	PBRB00000000	1.42	no	75.00	4.80	83.33	Tully, B. J. et al., 2018
REDSEA-S03-B6	REDSEA-S03-B6	MGIIb	NCBI	LURP00000000	1.32	yes	64.47	2.30	62.50	Haroon, M. F. *et al.,* 2016
REDSEA-S11-B3N4	REDSEA-S11-B3N4	MGIIb	NCBI	LURR00000000	1.30	yes	81.96	0.00	0.00	Haroon, M. F. *et al.,* 2016
REDSEA-S19-B7N8	REDSEA-S19-B7N8	MGIIb	NCBI	LURS00000000	1.17	yes	70.40	0.80	0.00	Haroon, M. F. *et al.,* 2016
REDSEA-S30-B12	REDSEA-S30-B12	MGIIb	NCBI	LURV00000000	1.27	no	67.79	0.80	0.00	Haroon, M. F. *et al.,* 2016
REDSEA-S40-B11N13	REDSEA-S40-B11N13	MGIIb	NCBI	LURX00000000	1.24	no	71.96	0.00	0.00	Haroon, M. F. *et al.,* 2016
REDSEA-S41-B6	REDSEA-S41-B6	MGIIb	NCBI	LURY00000000	1.13	no	71.35	1.12	66.67	Haroon, M. F. *et al.,* 2016
REDSEA-S43-B8	REDSEA-S43-B8	MGIIb	NCBI	LUSA00000000	1.10	yes	72.13	0.00	0.00	Haroon, M. F. *et al.,* 2016
RS348	RS348	MGIIb	NCBI	PBPV00000000	1.84	no	78.51	1.92	100.00	Tully, B. J. et al., 2018
RS354	RS354	MGIIb	NCBI	PBPQ00000000	1.25	no	68.24	1.60	50.00	Tully, B. J. et al., 2018
RS356	RS356	MGIIb	NCBI	PBPO00000000	0.74	no	52.28	0.00	0.00	Tully, B. J. et al., 2018
RS375	RS375	MGIIb	NCBI	PBPA00000000	1.08	no	65.07	0.00	0.00	Tully, B. J. et al., 2018
RS392	RS392	MGIIb	NCBI	PBOL00000000	1.41	no	76.40	0.80	0.00	Tully, B. J. et al., 2018
RS406	RS406	MGIIa	NCBI	PBWC00000000	2.03	no	80.93	1.60	100.00	Tully, B. J. et al., 2018
RS407	RS407	MGIIb	NCBI	PBWB00000000	1.11	no	56.93	0.80	100.00	Tully, B. J. et al., 2018
RS432	RS432	MGIIa	NCBI	PBVG00000000	1.79	no	70.00	4.00	80.00	Tully, B. J. et al., 2018
RS440	RS440	MGIIa	NCBI	PBUZ00000000	1.84	no	74.53	3.20	100.00	Tully, B. J. et al., 2018
RS443	RS443	MGIIb	NCBI	PBUW00000000	1.13	no	66.00	4.29	75.00	Tully, B. J. et al., 2018
RS462	RS462	MGIIb	NCBI	PBUH00000000	1.26	no	63.60	3.20	0.00	Tully, B. J. et al., 2018
RS604	RS604	MGIIa	NCBI	PBXF00000000	1.88	no	75.60	4.11	52.63	Tully, B. J. et al., 2018
RS814	RS814	MGIIb	NCBI	PBWO00000000	1.38	no	74.56	2.74	72.73	Tully, B. J. et al., 2018
SAT102	SAT102	MGIIb	NCBI	PAYN00000000	1.03	no	57.73	0.00	0.00	Tully, B. J. et al., 2018
SAT116	SAT116	MGIIb	NCBI	PAXY00000000	1.29	no	70.34	1.60	50.00	Tully, B. J. et al., 2018
SAT122	SAT122	MGIIa	NCBI	PAXR00000000	1.90	no	70.81	1.60	0.00	Tully, B. J. et al., 2018
SAT1329	SAT1329	MGIIa	NCBI	PAWY00000000	1.63	no	77.06	2.40	100.00	Tully, B. J. et al., 2018
SAT145	SAT145	MGIIa	NCBI	PAYA00000000	1.81	no	80.53	1.60	0.00	Tully, B. J. et al., 2018
SAT1460	SAT1460	MGIIb	NCBI	PAUZ00000000	1.09	no	59.61	2.40	33.33	Tully, B. J. et al., 2018
SAT1485	SAT1485	MGIIa	ANVIO-REFINED	figshare	1.27	no	56.40	3.20	60.00	Tully, B. J. et al., 2018
SAT1510	SAT1510	MGIIb	NCBI	PAUH00000000	1.55	no	75.02	4.00	37.50	Tully, B. J. et al., 2018
SAT1553	SAT1553	MGIIb	NCBI	PATZ00000000	1.59	no	76.27	4.00	33.33	Tully, B. J. et al., 2018
SAT1567	SAT1567	MGIIa	NCBI	PATV00000000	1.67	no	69.07	4.33	75.00	Tully, B. J. et al., 2018
SAT157	SAT157	MGIIa	NCBI	PATU00000000	2.48	no	81.20	4.27	66.67	Tully, B. J. et al., 2018
SAT164	SAT164	MGIIb	NCBI	PATD00000000	1.72	no	77.73	0.06	0.00	Tully, B. J. et al., 2018
SAT165	SAT165	MGIIb	NCBI	PATC00000000	1.70	no	77.69	0.80	100.00	Tully, B. J. et al., 2018
SAT190	SAT190	MGIIb	NCBI	PASD00000000	1.24	no	65.07	0.80	100.00	Tully, B. J. et al., 2018
SAT205	SAT205	MGIIa	NCBI	PAOW00000000	1.70	no	71.94	0.00	0.00	Tully, B. J. et al., 2018
SAT218	SAT218	MGIIb	NCBI	PAOK00000000	1.64	no	67.27	1.60	100.00	Tully, B. J. et al., 2018
SAT2681	SAT2681	MGIIb	NCBI	PANM00000000	1.14	no	62.78	0.80	0.00	Tully, B. J. et al., 2018
SAT2781	SAT2781	MGIIa	NCBI	PANB00000000	1.50	no	64.27	3.73	100.00	Tully, B. J. et al., 2018
SAT2971	SAT2971	MGIIa	ANVIO-REFINED	figshare	1.30	no	55.78	0.8	0.00	Tully, B. J. et al., 2018

SAT38	SAT38	MGIIa	NCBI	PALS00000000	1.89	no	80.13	3.07	83.33	Tully, B. J. et al., 2018
SAT95	SAT95	MGIIa	NCBI	PAJM00000000	1.89	no	81.60	0.80	100.00	Tully, B. J. et al., 2018
SP100	SP100	MGIIb	NCBI	PARS00000000	1.46	no	59.42	0.80	0.00	Tully, B. J. et al., 2018
SP143	SP143	MGIIa	NCBI	PAQE00000000	1.53	no	63.33	1.60	100.00	Tully, B. J. et al., 2018
SP170	SP170	MGIIa	NCBI	PAPE00000000	1.46	no	53.29	1.60	0.00	Tully, B. J. et al., 2018
SP175	SP175	MGIIb	NCBI	PAOZ00000000	1.77	no	62.67	1.60	0.00	Tully, B. J. et al., 2018
SP189	SP189	MGIIa	NCBI	PBNL00000000	1.70	no	81.74	3.23	25.00	Tully, B. J. et al., 2018
SP192	SP192	MGIIb	NCBI	PBNI00000000	0.87	no	52.44	0.00	0.00	Tully, B. J. et al., 2018
SP210	SP210	MGIIb	NCBI	PBMO000000000	1.64	no	77.16	1.60	50.00	Tully, B. J. et al., 2018
SP212	SP212	MGIIa	NCBI	PBMM00000000	1.34	no	59.33	1.60	50.00	Tully, B. J. et al., 2018
SP231	SP231	MGIIb	NCBI	PBLT00000000	0.81	no	52.42	1.60	50.00	Tully, B. J. et al., 2018
SP272	SP272	MGIIa	NCBI	PBKJ00000000	1.94	no	79.76	1.60	50.00	Tully, B. J. et al., 2018
SP277	SP277	MGIIb	NCBI	PBKE00000000	1.13	yes	64.67	0.38	0.00	Tully, B. J. et al., 2018
SP3007	SP3007	MGIIb	NCBI	PBIF00000000	1.09	yes	54.22	3.60	80.00	Tully, B. J. et al., 2018
SP3014	SP3014	MGIIb	NCBI	PBHZ00000000	1.67	no	75.33	2.40	66.67	Tully, B. J. et al., 2018
SP3024	SP3024	MGIIa	NCBI	PBHR00000000	1.82	no	77.73	3.26	20.00	Tully, B. J. et al., 2018
SP3025	SP3025	MGIIa	NCBI	PBHQ00000000	1.89	no	74.67	3.36	66.67	Tully, B. J. et al., 2018
SP3027	SP3027	MGIIb	NCBI	PBHP00000000	1.18	no	54.22	4.59	71.43	Tully, B. J. et al., 2018
SP303	SP303	MGIIa	NCBI	PBHM00000000	1.74	no	73.20	1.60	50.00	Tully, B. J. et al., 2018
SP3038	SP3038	MGIIb	NCBI	PBHF00000000	1.32	no	64.02	2.13	100.00	Tully, B. J. et al., 2018
SP3051	SP3051	MGIIb	NCBI	PBGT00000000	1.65	no	60.99	2.40	66.67	Tully, B. J. et al., 2018
SP3054	SP3054	MGIIa	NCBI	PBGS00000000	2.03	no	74.93	4.00	66.67	Tully, B. J. et al., 2018
SP3059	SP3059	MGIIa	ANVIO-REFINED	figshare	1.80	yes	81.20	0.80	100.00	Tully, B. J. et al., 2018
SP3063	SP3063	MGIIb	ANVIO-REFINED	figshare	1.48	no	86.40	2.40	66.67	Tully, B. J. et al., 2018
SP3066	SP3066	MGIIa	NCBI	PBGJ00000000	1.53	no	70.93	3.60	100.00	Tully, B. J. et al., 2018
SP3067	SP3067	MGIIb	NCBI	PBGI00000000	1.14	no	58.47	4.80	100.00	Tully, B. J. et al., 2018
SP3068	SP3068	MGIIb	NCBI	PBGH00000000	1.56	no	76.81	4.80	100.00	Tully, B. J. et al., 2018
SP3088	SP3088	MGIIb	NCBI	PBFT00000000	1.44	no	58.74	4.00	11.11	Tully, B. J. et al., 2018
SP3092	SP3092	MGIIb	NCBI	PBFP00000000	1.36	no	67.20	2.53	28.57	Tully, B. J. et al., 2018
SP3093	SP3093	MGIIb	NCBI	PBFO00000000	1.17	no	58.67	3.20	25.00	Tully, B. J. et al., 2018
SP3107	SP3107	MGIIb	NCBI	PBFF00000000	1.30	no	73.78	3.20	83.33	Tully, B. J. et al., 2018
SP3115	SP3115	MGIIb	NCBI	PBEZ00000000	0.87	no	53.60	4.00	22.22	Tully, B. J. et al., 2018
SP338	SP338	MGIIb	NCBI	PBDV00000000	1.58	no	67.02	0.00	0.00	Tully, B. J. et al., 2018
SP339	SP339	MGIIa	NCBI	PBDU00000000	2.03	no	68.80	1.52	0.00	Tully, B. J. et al., 2018
SP42	SP42	MGIIb	NCBI	PBBV00000000	1.21	no	66.09	0.00	0.00	Tully, B. J. et al., 2018
SP67	SP67	MGIIa	NCBI	PAZU00000000	1.94	no	63.96	0.80	0.00	Tully, B. J. et al., 2018
SP88	SP88	MGIIb	NCBI	PAYZ00000000	1.58	no	79.07	0.80	0.00	Tully, B. J. et al., 2018
TARA-ANE-MAG-00019	TARA-ANE-MAG-00019	MGIIb	figshare	figshare	1.85	no	84.53	1.60	0.00	Delmont, T. O. *et al.,* 2018
TARA-ANE-MAG-00024	TARA-ANE-MAG-00024	MGIIa	figshare	figshare	1.89	no	82.93	0.00	0.00	Delmont, T. O. *et al.,* 2018
TARA-ANE-MAG-00027	TARA-ANE-MAG-00027	MGIIa	figshare	figshare	1.92	no	79.60	0.80	0.00	Delmont, T. O. *et al.,* 2018
TARA-ANE-MAG-00030	TARA-ANE-MAG-00030	MGIIa	figshare	figshare	1.65	no	80.40	2.86	20.00	Delmont, T. O. *et al.,* 2018
TARA-ANE-MAG-00052	TARA-ANE-MAG-00052	MGIIb	figshare	figshare	1.25	no	68.90	0.00	0.00	Delmont, T. O. *et al.,* 2018
TARA-ANE-MAG-00055	TARA-ANE-MAG-00055	MGIIa	figshare	figshare	1.54	no	72.69	0.00	0.00	Delmont, T. O. *et al.,* 2018
TARA-ANE-MAG-00063	TARA-ANE-MAG-00063	MGIIb	figshare	figshare	1.14	no	60.82	0.00	0.00	Delmont, T. O. *et al.,* 2018
TARA-ANE-MAG-00065	TARA-ANE-MAG-00065	MGIIb	figshare	figshare	1.55	no	64.00	1.60	66.67	Delmont, T. O. *et al.,* 2018
TARA-ANW-MAG-00024	TARA-ANW-MAG-00024	MGIIa	figshare	figshare	1.81	no	80.00	0.04	100.00	Delmont, T. O. *et al.,* 2018
TARA-ANW-MAG-00033	TARA-ANW-MAG-00033	MGIIb	figshare	figshare	1.32	no	75.34	0.00	0.00	Delmont, T. O. *et al.,* 2018
TARA-ANW-MAG-00043	TARA-ANW-MAG-00043	MGIIb	figshare	figshare	1.18	no	64.27	0.50	100.00	Delmont, T. O. *et al.,* 2018
TARA-ANW-MAG-00045	TARA-ANW-MAG-00045	MGIIb	figshare	figshare	1.63	no	66.53	0.00	0.00	Delmont, T. O. *et al.,* 2018
TARA-ANW-MAG-00046	TARA-ASE-MAG-00009	MGIIb	figshare	figshare	1.01	no	65.20	0.80	100.00	Delmont, T. O. *et al.,* 2018
TARA-ASE-MAG-00009	TARA-ASE-MAG-00011	MGIIb	figshare	figshare	1.47	no	61.07	4.04	42.86	Delmont, T. O. *et al.,* 2018
TARA-ASE-MAG-00011	TARA-ASE-MAG-00011	MGIIb	figshare	figshare	1.47	no	25.33	0.16	n.d.	Delmont, T. O. *et al.,* 2018
TARA-ASE-MAG-00016	TARA-ASE-MAG-00016	MGIIb	figshare	figshare	1.35	no	70.56	5.78	22.22	Delmont, T. O. *et al.,* 2018
TARA-ASE-MAG-00018	TARA-ASE-MAG-00018	MGIIa	figshare	figshare	1.43	no	64.51	0.00	0.00	Delmont, T. O. *et al.,* 2018
TARA-ASW-MAG-00011	TARA-ASW-MAG-00011	MGIIa	figshare	figshare	1.96	no	83.20	0.00	0.00	Delmont, T. O. *et al.,* 2018
TARA-ASW-MAG-00013	TARA-ASW-MAG-00013	MGIIb	figshare	figshare	1.24	no	70.93	0.00	0.00	Delmont, T. O. *et al.,* 2018
TARA-ASW-MAG-00017	TARA-ASW-MAG-00017	MGIIa	figshare	figshare	1.83	no	71.69	1.87	0.00	Delmont, T. O. *et al.,* 2018
TARA-ASW-MAG-00019	TARA-ASW-MAG-00019	MGIIa	figshare	figshare	1.51	no	66.67	0.84	25.00	Delmont, T. O. *et al.,* 2018
TARA-ASW-MAG-00021	TARA-ASW-MAG-00021	MGIIb	figshare	figshare	1.15	no	66.09	1.73	100.00	Delmont, T. O. *et al.,* 2018

TARA-ASW-MAG-00024	TARA-ASW-MAG-00024	MGIIb	figshare	figshare	1.09	no	63.87	5.20	33.33	Delmont, T. O. *et al.,* 2018
TARA-ION-MAG-00033	TARA-ION-MAG-00033	MGIIb	figshare	figshare	1.51	no	83.27	0.93	50.00	Delmont, T. O. *et al.,* 2018
TARA-ION-MAG-00034	TARA-ION-MAG-00034	MGIIb	figshare	figshare	1.32	no	70.09	0.80	0.00	Delmont, T. O. *et al.,* 2018
TARA-ION-MAG-00040	TARA-ION-MAG-00040	MGIIb	figshare	figshare	1.20	no	64.13	0.92	33.33	Delmont, T. O. *et al.,* 2018
TARA-ION-MAG-00044	TARA-ION-MAG-00044	MGIIb	figshare	figshare	1.27	no	74.42	0.00	0.00	Delmont, T. O. *et al.,* 2018
TARA-IOS-MAG-00022	TARA-IOS-MAG-00022	MGIIb	figshare	figshare	1.34	no	79.87	0.80	100.00	Delmont, T. O. *et al.,* 2018
TARA-IOS-MAG-00030	TARA-IOS-MAG-00030	MGIIb	figshare	figshare	1.23	no	73.47	0.00	0.00	Delmont, T. O. *et al.,* 2018
TARA-IOS-MAG-00038	TARA-IOS-MAG-00038	MGIIa	figshare	figshare	1.55	no	69.15	2.40	33.33	Delmont, T. O. *et al.,* 2018
TARA-IOS-MAG-00039	TARA-IOS-MAG-00039	MGIIb	figshare	figshare	1.27	no	73.43	2.40	33.33	Delmont, T. O. *et al.,* 2018
TARA-IOS-MAG-00047	TARA-IOS-MAG-00047	MGIIa	figshare	figshare	1.63	no	69.76	0.40	100.00	Delmont, T. O. *et al.,* 2018
TARA-MED-MAG-00060	TARA-MED-MAG-00060	MGIIa	figshare	figshare	1.62	no	77.14	1.82	80.00	Delmont, T. O. *et al.,* 2018
TARA-MED-MAG-00075	TARA-MED-MAG-00075	MGIIb	figshare	figshare	1.35	no	79.87	0.00	0.00	Delmont, T. O. *et al.,* 2018
TARA-MED-MAG-00079	TARA-MED-MAG-00079	MGIIa	figshare	figshare	1.71	no	73.33	0.00	0.00	Delmont, T. O. *et al.,* 2018
TARA-MED-MAG-00080	TARA-MED-MAG-00080	MGIIb	figshare	figshare	1.77	no	78.40	0.80	0.00	Delmont, T. O. *et al.,* 2018
TARA-MED-MAG-00095	TARA-MED-MAG-00095	MGIIb	figshare	figshare	1.41	no	76.71	0.00	0.00	Delmont, T. O. *et al.,* 2018
TARA-PON-MAG-00033	TARA-PON-MAG-00033	MGIIa	figshare	figshare	1.84	no	75.73	0.00	0.00	Delmont, T. O. *et al.,* 2018
TARA-PON-MAG-00047	TARA-PON-MAG-00047	MGIIb	figshare	figshare	1.22	no	61.69	3.66	66.67	Delmont, T. O. *et al.,* 2018
TARA-PON-MAG-00049	TARA-PON-MAG-00049	MGIIb	figshare	figshare	0.95	no	59.96	0.16	0.00	Delmont, T. O. *et al.,* 2018
TARA-PSE-MAG-00061	TARA-PSE-MAG-00061	MGIIb	figshare	figshare	1.64	no	75.12	2.27	0.00	Delmont, T. O. *et al.,* 2018
TARA-PSE-MAG-00064	TARA-PSE-MAG-00064	MGIIb	figshare	figshare	1.25	no	69.47	1.60	50.00	Delmont, T. O. *et al.,* 2018
TARA-PSE-MAG-00070	TARA-PSE-MAG-00070	MGIIb	figshare	figshare	1.14	no	64.94	0.80	50.00	Delmont, T. O. *et al.,* 2018
TARA-PSW-MAG-00056	TARA-PSW-MAG-00056	MGIIa	figshare	figshare	1.86	no	80.80	1.60	100.00	Delmont, T. O. *et al.,* 2018
TARA-PSW-MAG-00057	TARA-PSW-MAG-00057	MGIIb	figshare	figshare	1.44	no	76.40	0.00	0.00	Delmont, T. O. *et al.,* 2018
TARA-PSW-MAG-00073	TARA-PSW-MAG-00073	MGIIb	figshare	figshare	1.14	no	71.96	0.04	0.00	Delmont, T. O. *et al.,* 2018
TARA-PSW-MAG-00075	TARA-PSW-MAG-00075	MGIIb	figshare	figshare	1.42	no	80.27	0.00	0.00	Delmont, T. O. *et al.,* 2018
TARA-PSW-MAG-00086	TARA-PSW-MAG-00086	MGIIa	figshare	figshare	1.91	no	70.00	1.42	50.00	Delmont, T. O. *et al.,* 2018
TARA-PSW-MAG-00101	TARA-PSW-MAG-00101	MGIIa	figshare	figshare	1.35	no	60.61	0.80	100.00	Delmont, T. O. *et al.,* 2018
TARA-PSW-MAG-00102	TARA-PSW-MAG-00102	MGIIa	figshare	figshare	1.51	no	63.21	0.40	100.00	Delmont, T. O. *et al.,* 2018
TARA-RED-MAG-00049	TARA-RED-MAG-00049	MGIIb	figshare	figshare	1.57	no	74.56	0.96	50.00	Delmont, T. O. *et al.,* 2018
TARA-RED-MAG-00055	TARA-RED-MAG-00055	MGIIa	figshare	figshare	1.82	no	79.60	0.06	0.00	Delmont, T. O. *et al.,* 2018
TARA-RED-MAG-00062	TARA-RED-MAG-00062	MGIIb	figshare	figshare	1.27	no	74.22	0.40	0.00	Delmont, T. O. *et al.,* 2018
TARA-RED-MAG-00072	TARA-RED-MAG-00072	MGIIa	figshare	figshare	1.64	no	71.73	1.60	50.00	Delmont, T. O. *et al.,* 2018
TMED129	TMED129	MGIIb	NCBI	NHGE00000000	1.40	no	69.33	0.00	0.00	Tully, B. J. et al., 2017
TMED132	TMED132	MGIIa	ANVIO-REFINED	figshare	1.50	no	66.67	1.72	75.00	Tully, B. J. et al., 2017
TMED141	TMED141	MGIIa	NCBI	NHGQ00000000	2.36	no	67.53	4.00	0.00	Tully, B. J. et al., 2017
TMED248	TMED248	MGIIa	NCBI	NHKT00000000	1.12	no	52.67	1.60	100.00	Tully, B. J. et al., 2017
TMED97	TMED97	MGIIb	NCBI	NHEY00000000	1.63	no	76.74	0.80	0.00	Tully, B. J. et al., 2017
TMED99	TMED99	MGIIa	NCBI	NHFA00000000	2.09	no	76.53	3.24	33.33	Tully, B. J. et al., 2017
UBA10	UBA10	MGIIa	NCBI	ERX556126	1.71	no	75.09	0.00	0.00	Parks, D. H. *et al.,* 2017
UBA100	UBA100	MGIIa	NCBI	ERX556031	1.55	no	66.72	0.80	0.00	Parks, D. H. *et al.,* 2017
UBA101	UBA101	MGIIb	NCBI	ERX556108	1.12	no	67.73	2.00	33.33	Parks, D. H. *et al.,* 2017
UBA103	UBA103	MGIIb	NCBI	ERX556101	1.31	no	77.61	0.00	0.00	Parks, D. H. *et al.,* 2017
UBA104	UBA104	MGIIb	NCBI	ERX556101	1.39	no	66.74	0.00	0.00	Parks, D. H. *et al.,* 2017
UBA105	UBA105	MGIIb	NCBI	ERX556101	1.35	no	73.28	2.04	25.00	Parks, D. H. *et al.,* 2017
UBA106	UBA106	MGIIb	NCBI	ERX556103	1.08	no	64.13	0.00	0.00	Parks, D. H. *et al.,* 2017
UBA107	UBA107	MGIIa	NCBI	ERX556103	1.68	no	69.73	0.97	0.00	Parks, D. H. *et al.,* 2017
UBA108	UBA108	MGIIb	NCBI	ERX556103	0.91	no	57.40	0.00	0.00	Parks, D. H. *et al.,* 2017
UBA109	UBA109	MGIIb	NCBI	ERX556103	1.24	no	67.60	0.80	100.00	Parks, D. H. *et al.,* 2017
UBA11	UBA11	MGIIa	NCBI	ERX556126	1.86	no	72.76	0.00	0.00	Parks, D. H. *et al.,* 2017
UBA110	UBA110	MGIIa	NCBI	ERX556103	1.81	no	76.31	0.00	0.00	Parks, D. H. *et al.,* 2017
UBA111	UBA111	MGIIa	NCBI	ERX556106	1.43	no	59.25	0.80	0.00	Parks, D. H. *et al.,* 2017
UBA112	UBA112	MGIIb	NCBI	ERX556105	0.86	no	55.45	0.00	0.00	Parks, D. H. *et al.,* 2017

UBA12	UBA12	MGIIa	NCBI	ERX556126	1.89	no	80.53	0.80	0.00	Parks, D. H. *et al.*, 2017
UBA120	UBA120	MGIIa	NCBI	ERX555919	1.72	no	75.56	0.80	100.00	Parks, D. H. *et al.*, 2017
UBA121	UBA121	MGIIb	NCBI	ERX555918	1.30	no	73.82	0.08	0.00	Parks, D. H. *et al.*, 2017
UBA122	UBA122	MGIIa	NCBI	ERX555918	1.70	no	72.00	0.00	0.00	Parks, D. H. *et al.*, 2017
UBA123	UBA123	MGIIa	NCBI	ERX555914	1.87	yes	80.53	4.00	0.00	Parks, D. H. *et al.*, 2017
UBA124	UBA124	MGIIa	NCBI	ERX555914	1.88	no	80.00	0.00	0.00	Parks, D. H. *et al.*, 2017
UBA125	UBA125	MGIIb	NCBI	ERX555914	1.82	no	80.51	0.04	100.00	Parks, D. H. *et al.*, 2017
UBA126	UBA126	MGIIb	NCBI	ERX555917	1.45	no	82.51	0.93	100.00	Parks, D. H. *et al.*, 2017
UBA127	UBA127	MGIIb	NCBI	ERX555916	1.16	yes	60.56	0.80	0.00	Parks, D. H. *et al.*, 2017
UBA128	UBA128	MGIIa	NCBI	ERX555916	1.62	no	80.73	3.52	33.33	Parks, D. H. *et al.*, 2017
UBA129	UBA129	MGIIb	NCBI	ERX555913	1.16	no	66.09	2.40	0.00	Parks, D. H. *et al.*, 2017
UBA13	UBA13	MGIIa	NCBI	ERX556126	1.60	no	73.33	2.40	0.00	Parks, D. H. *et al.*, 2017
UBA130	UBA130	MGIIb	NCBI	ERX555912	1.35	no	80.13	0.00	0.00	Parks, D. H. *et al.*, 2017
UBA131	UBA131	MGIIa	NCBI	ERX555912	1.86	no	81.19	0.80	0.00	Parks, D. H. *et al.*, 2017
UBA132	UBA132	MGIIb	NCBI	SRX802143	1.57	yes	83.73	0.00	0.00	Parks, D. H. *et al.*, 2017
UBA133	UBA133	MGIIb	NCBI	SRX1044556	1.39	yes	71.32	0.80	100.00	Parks, D. H. *et al.*, 2017
UBA134	UBA134	MGIIb	NCBI	SRX1044554	1.21	yes	56.85	0.00	0.00	Parks, D. H. *et al.*, 2017
UBA137	UBA137	MGIIb	NCBI	ERX556022	1.22	no	68.80	0.00	0.00	Parks, D. H. *et al.*, 2017
UBA14	UBA14	MGIIb	NCBI	ERX556126	1.12	no	62.13	0.80	0.00	Parks, D. H. *et al.*, 2017
UBA15	UBA15	MGIIa	NCBI	ERX556126	2.33	no	72.27	4.00	0.00	Parks, D. H. *et al.*, 2017
UBA16	UBA16	MGIIa	NCBI	ERX556129	1.66	no	77.81	0.80	0.00	Parks, D. H. *et al.*, 2017
UBA17	UBA17	MGIIa	NCBI	ERX556129	1.68	no	73.33	0.80	0.00	Parks, D. H. *et al.*, 2017
UBA170	UBA170	MGIIb	NCBI	ERX555907	1.12	no	64.62	0.00	0.00	Parks, D. H. *et al.*, 2017
UBA171	UBA171	MGIIb	NCBI	ERX555907	1.34	no	74.40	0.00	0.00	Parks, D. H. *et al.*, 2017
UBA172	UBA172	MGIIb	NCBI	SRX1044543	1.23	no	64.94	0.00	0.00	Parks, D. H. *et al.*, 2017
UBA173	UBA173	MGIIb	NCBI	ERX556040	1.29	no	68.69	0.00	0.00	Parks, D. H. *et al.*, 2017
UBA174	UBA174	MGIIb	NCBI	ERX556040	1.36	no	68.80	3.33	14.29	Parks, D. H. *et al.*, 2017
UBA175	UBA175	MGIIb	NCBI	ERX556040	1.27	no	70.00	0.00	0.00	Parks, D. H. *et al.*, 2017
UBA177	UBA177	MGIIa	NCBI	ERX556059	1.50	no	62.47	0.00	0.00	Parks, D. H. *et al.*, 2017
UBA178	UBA178	MGIIb	NCBI	ERX556059	1.30	no	66.55	0.00	0.00	Parks, D. H. *et al.*, 2017
UBA179	UBA179	MGIIa	NCBI	ERX556059	1.82	no	78.13	0.00	0.00	Parks, D. H. *et al.*, 2017
UBA18	UBA18	MGIIb	NCBI	ERX552241	1.29	no	71.20	0.00	0.00	Parks, D. H. *et al.*, 2017
UBA180	UBA180	MGIIb	NCBI	ERX556054	1.35	no	69.80	2.40	0.00	Parks, D. H. *et al.*, 2017
UBA181	UBA181	MGIIb	NCBI	ERX556054	1.33	no	77.41	0.80	0.00	Parks, D. H. *et al.*, 2017
UBA182	UBA182	MGIIb	NCBI	ERX556056	1.37	no	73.20	0.00	0.00	Parks, D. H. *et al.*, 2017
UBA19	UBA19	MGIIb	NCBI	ERX552241	1.39	no	75.33	0.00	0.00	Parks, D. H. *et al.*, 2017
UBA191	UBA191	MGIIb	NCBI	ERX555933	1.29	yes	66.00	0.00	0.00	Parks, D. H. *et al.*, 2017
UBA192	UBA192	MGIIb	NCBI	ERX555931	1.00	no	51.83	0.00	0.00	Parks, D. H. *et al.*, 2017
UBA193	UBA193	MGIIb	NCBI	ERX555931	1.19	no	67.27	0.00	0.00	Parks, D. H. *et al.*, 2017
UBA194	UBA194	MGIIb	NCBI	ERX555931	1.09	no	60.40	0.80	0.00	Parks, D. H. *et al.*, 2017
UBA195	UBA195	MGIIb	NCBI	ERX555931	1.31	no	72.40	1.47	0.00	Parks, D. H. *et al.*, 2017
UBA196	UBA196	MGIIb	NCBI	ERX555931	1.82	no	72.40	3.32	28.57	Parks, D. H. *et al.*, 2017
UBA197	UBA197	MGIIb	NCBI	ERX555932	1.08	no	64.36	0.53	50.00	Parks, D. H. *et al.*, 2017
UBA199	UBA199	MGIIb	NCBI	ERX555932	1.55	no	84.00	2.53	0.00	Parks, D. H. *et al.*, 2017
UBA20	UBA20	MGIIb	NCBI	ERX552241	1.40	no	76.40	0.00	0.00	Parks, D. H. *et al.*, 2017
UBA200	UBA200	MGIIb	NCBI	ERX555932	1.07	no	62.00	0.00	0.00	Parks, D. H. *et al.*, 2017
UBA209	UBA209	MGIIb	NCBI	SRX648501	1.28	no	66.93	1.60	0.00	Parks, D. H. *et al.*, 2017
UBA21	UBA21	MGIIa	NCBI	ERX552241	1.14	no	51.87	0.00	0.00	Parks, D. H. *et al.*, 2017
UBA214	UBA214	MGIIa	NCBI	ERX552303	1.29	no	62.53	0.00	0.00	Parks, D. H. *et al.*, 2017
UBA215	UBA215	MGIIb	NCBI	ERX552302	0.77	no	52.14	0.00	0.00	Parks, D. H. *et al.*, 2017
UBA22	UBA22	MGIIb	NCBI	ERX552243	1.18	no	64.30	0.00	0.00	Parks, D. H. *et al.*, 2017
UBA220	UBA220	MGIIb	NCBI	ERX556028	1.31	no	79.36	1.60	0.00	Parks, D. H. *et al.*, 2017
UBA221	UBA221	MGIIb	NCBI	ERX556028	1.47	no	56.40	0.00	0.00	Parks, D. H. *et al.*, 2017
UBA222	UBA222	MGIIb	NCBI	ERX556028	1.15	no	57.23	0.80	100.00	Parks, D. H. *et al.*, 2017
UBA224	UBA224	MGIIb	NCBI	ERX556075	1.33	no	74.27	0.80	0.00	Parks, D. H. *et al.*, 2017
UBA225	UBA225	MGIIb	NCBI	ERX556098	1.22	no	64.22	0.00	0.00	Parks, D. H. *et al.*, 2017
UBA226	UBA226	MGIIa	NCBI	ERX556098	1.62	no	71.07	0.00	0.00	Parks, D. H. *et al.*, 2017
UBA227	UBA227	MGIIb	NCBI	ERX556091	1.03	no	51.28	0.00	0.00	Parks, D. H. *et al.*, 2017
UBA228	UBA228	MGIIb	NCBI	ERX556091	1.27	no	63.10	2.40	0.00	Parks, D. H. *et al.*, 2017
UBA229	UBA229	MGIIa	NCBI	ERX556094	1.72	no	75.29	1.60	0.00	Parks, D. H. *et al.*, 2017
UBA23	UBA23	MGIIb	NCBI	ERX552243	1.49	no	84.93	0.00	0.00	Parks, D. H. *et al.*, 2017
UBA242	UBA242	MGIIa	NCBI	SRX1050770	1.81	yes	82.40	0.00	0.00	Parks, D. H. *et al.*, 2017
UBA249	UBA249	MGIIb	NCBI	ERX556065	1.34	no	77.12	0.53	0.00	Parks, D. H. *et al.*, 2017
UBA25	UBA25	MGIIb	NCBI	ERX552244	1.10	no	61.83	1.60	100.00	Parks, D. H. *et al.*, 2017
UBA250	UBA250	MGIIb	NCBI	ERX556060	1.26	no	75.20	0.80	0.00	Parks, D. H. *et al.*, 2017
UBA251	UBA251	MGIIb	NCBI	ERX556060	1.76	no	81.27	1.60	50.00	Parks, D. H. *et al.*, 2017
UBA252	UBA252	MGIIb	NCBI	ERX556063	1.39	no	82.80	0.00	0.00	Parks, D. H. *et al.*, 2017
UBA253	UBA253	MGIIa	NCBI	ERX556088	1.17	no	50.18	0.00	0.00	Parks, D. H. *et al.*, 2017
UBA255	UBA255	MGIIa	NCBI	SRX802077	1.94	yes	83.47	0.00	0.00	Parks, D. H. *et al.*, 2017
UBA256	UBA256	MGIIa	NCBI	SRX802076	1.79	yes	84.13	0.00	0.00	Parks, D. H. *et al.*, 2017
UBA257	UBA257	MGIIb	NCBI	SRX802076	1.21	no	69.60	0.80	0.00	Parks, D. H. *et al.*, 2017
UBA258	UBA258	MGIIa	NCBI	SRX802076	1.88	no	73.07	4.53	37.50	Parks, D. H. *et al.*, 2017
UBA259	UBA259	MGIIa	NCBI	SRX802076	1.17	no	67.47	0.00	0.00	Parks, D. H. *et al.*, 2017
UBA26	UBA26	MGIIb	NCBI	ERX552249	1.36	no	75.20	0.00	0.00	Parks, D. H. *et al.*, 2017
UBA260	UBA260	MGIIb	NCBI	SRX802076	1.45	no	73.26	3.07	60.00	Parks, D. H. *et al.*, 2017
UBA27	UBA27	MGIIb	NCBI	ERX552249	1.78	no	74.80	0.00	0.00	Parks, D. H. *et al.*, 2017
UBA28	UBA28	MGIIb	NCBI	ERX552249	1.29	no	73.33	0.00	0.00	Parks, D. H. *et al.*, 2017
UBA29	UBA29	MGIIb	NCBI	SRX672316	1.46	no	68.22	0.00	0.00	Parks, D. H. *et al.*, 2017
UBA3	UBA3	MGIIb	NCBI	ERX556017	0.95	no	52.10	0.00	0.00	Parks, D. H. *et al.*, 2017
UBA30	UBA30	MGIIb	NCBI	SRX672316	1.35	no	66.00	0.00	0.00	Parks, D. H. *et al.*, 2017
UBA31	UBA31	MGIIb	NCBI	SRX672316	1.29	no	63.73	0.00	0.00	Parks, D. H. *et al.*, 2017
UBA34	UBA34	MGIIb	NCBI	ERX555973	1.40	no	75.88	2.40	60.00	Parks, D. H. *et al.*, 2017
UBA35	UBA35	MGIIa	NCBI	ERX555971	1.43	no	60.67	2.00	0.00	Parks, D. H. *et al.*, 2017
UBA36	UBA36	MGIIb	NCBI	ERX555978	1.34	no	69.72	1.20	100.00	Parks, D. H. *et al.*, 2017

UBA37	UBA37	MGIIa	NCBI	SRX803008	1.83	yes	80.58	0.00	0.00	Parks, D. H. *et al.*, 2017
UBA38	UBA38	MGIIb	NCBI	ERX556003	0.99	no	52.13	0.00	0.00	Parks, D. H. *et al.*, 2017
UBA39	UBA39	MGIIb	NCBI	ERX556005	1.58	no	64.94	1.33	33.33	Parks, D. H. *et al.*, 2017
UBA4	UBA4	MGIIb	NCBI	ERX556017	0.93	no	55.47	0.00	0.00	Parks, D. H. *et al.*, 2017
UBA40	UBA40	MGIIb	NCBI	ERX556005	1.80	no	78.67	1.64	0.00	Parks, D. H. *et al.*, 2017
UBA41	UBA41	MGIIa	NCBI	ERX556009	1.91	no	80.48	1.60	33.33	Parks, D. H. *et al.*, 2017
UBA433	UBA433	MGIIa	NCBI	SRX672291	1.84	no	80.02	1.60	50.00	Parks, D. H. *et al.*, 2017
UBA438	UBA438	MGIIb	NCBI	ERX555987	1.28	no	72.93	0.00	0.00	Parks, D. H. *et al.*, 2017
UBA439	UBA439	MGIIb	NCBI	ERX555987	1.39	no	81.73	0.00	0.00	Parks, D. H. *et al.*, 2017
UBA441	UBA441	MGIIa	NCBI	ERX555984	1.70	no	75.61	2.66	9.09	Parks, D. H. *et al.*, 2017
UBA442	UBA442	MGIIa	NCBI	ERX555984	1.76	no	77.84	0.04	100.00	Parks, D. H. *et al.*, 2017
UBA443	UBA443	MGIIa	NCBI	ERX555984	1.85	no	82.93	0.00	0.00	Parks, D. H. *et al.*, 2017
UBA461	UBA461	MGIIa	NCBI	SRX514547	1.88	no	83.42	0.00	0.00	Parks, D. H. *et al.*, 2017
UBA462	UBA462	MGIIb	NCBI	SRX514548	1.25	no	69.96	0.00	0.00	Parks, D. H. *et al.*, 2017
UBA463	UBA463	MGIIb	NCBI	SRX514548	1.34	yes	80.93	0.00	0.00	Parks, D. H. *et al.*, 2017
UBA464	UBA464	MGIIb	NCBI	SRX514549	1.23	no	70.76	0.00	0.00	Parks, D. H. *et al.*, 2017
UBA465	UBA465	MGIIb	NCBI	SRX514549	1.36	no	79.73	3.20	33.33	Parks, D. H. *et al.*, 2017
UBA484	UBA484	MGIIa	NCBI	ERX552297	2.00	no	83.33	3.60	0.00	Parks, D. H. *et al.*, 2017
UBA485	UBA485	MGIIb	NCBI	ERX552297	1.23	no	71.07	0.80	100.00	Parks, D. H. *et al.*, 2017
UBA486	UBA486	MGIIa	NCBI	ERX552297	1.80	no	76.36	0.80	0.00	Parks, D. H. *et al.*, 2017
UBA487	UBA487	MGIIb	NCBI	ERX552297	1.27	no	66.00	0.00	0.00	Parks, D. H. *et al.*, 2017
UBA49	UBA49	MGIIb	NCBI	ERX556134	1.04	no	57.20	0.80	0.00	Parks, D. H. *et al.*, 2017
UBA494	UBA494	MGIIb	NCBI	ERX552298	1.19	no	71.69	0.00	0.00	Parks, D. H. *et al.*, 2017
UBA495	UBA495	MGIIa	NCBI	ERX552298	1.87	no	79.60	0.80	0.00	Parks, D. H. *et al.*, 2017
UBA496	UBA496	MGIIb	NCBI	ERX552298	1.56	no	68.23	1.60	50.00	Parks, D. H. *et al.*, 2017
UBA497	UBA497	MGIIb	NCBI	ERX552274	1.43	no	82.27	0.00	0.00	Parks, D. H. *et al.*, 2017
UBA498	UBA498	MGIIb	NCBI	ERX552274	1.26	no	72.13	0.13	100.00	Parks, D. H. *et al.*, 2017
UBA499	UBA499	MGIIb	NCBI	ERX552275	1.16	no	63.99	0.00	0.00	Parks, D. H. *et al.*, 2017
UBA50	UBA50	MGIIb	NCBI	ERX556134	1.39	no	73.60	0.00	0.00	Parks, D. H. *et al.*, 2017
UBA500	UBA500	MGIIb	NCBI	ERX552275	1.50	no	84.93	0.80	0.00	Parks, D. H. *et al.*, 2017
UBA501	UBA501	MGIIb	NCBI	ERX552275	1.51	no	71.60	1.60	50.00	Parks, D. H. *et al.*, 2017
UBA502	UBA502	MGIIb	NCBI	ERX552272	1.08	no	56.13	0.00	0.00	Parks, D. H. *et al.*, 2017
UBA503	UBA503	MGIIb	NCBI	ERX552277	1.18	no	62.23	0.00	0.00	Parks, D. H. *et al.*, 2017
UBA51	UBA51	MGIIb	NCBI	ERX556134	1.39	no	69.60	2.40	0.00	Parks, D. H. *et al.*, 2017
UBA52	UBA52	MGIIb	NCBI	ERX556134	1.44	no	76.40	2.40	25.00	Parks, D. H. *et al.*, 2017
UBA528	UBA528	MGIIb	NCBI	SRX147858	1.11	no	61.92	0.93	25.00	Parks, D. H. *et al.*, 2017
UBA529	UBA529	MGIIb	NCBI	ERX555990	1.37	no	76.40	0.00	0.00	Parks, D. H. *et al.*, 2017
UBA53	UBA53	MGIIa	NCBI	ERX556130	1.73	yes	80.22	0.00	0.00	Parks, D. H. *et al.*, 2017
UBA530	UBA530	MGIIb	NCBI	ERX555990	1.62	no	71.42	0.80	0.00	Parks, D. H. *et al.*, 2017
UBA531	UBA531	MGIIb	NCBI	ERX555990	1.35	no	71.94	0.00	0.00	Parks, D. H. *et al.*, 2017
UBA538	UBA538	MGIIb	NCBI	ERX555999	1.20	no	68.27	0.00	0.00	Parks, D. H. *et al.*, 2017
UBA54	UBA54	MGIIb	NCBI	ERX556139	1.13	no	59.73	0.00	0.00	Parks, D. H. *et al.*, 2017
UBA540	UBA540	MGIIb	NCBI	ERX552281	1.10	no	55.57	0.00	0.00	Parks, D. H. *et al.*, 2017
UBA542	UBA542	MGIIb	NCBI	ERX552268	0.99	no	53.23	0.00	0.00	Parks, D. H. *et al.*, 2017
UBA545	UBA545	MGIIa	NCBI	ERX552262	1.53	no	68.32	2.67	20.00	Parks, D. H. *et al.*, 2017
UBA546	UBA546	MGIIa	NCBI	ERX552262	1.70	no	68.27	0.80	100.00	Parks, D. H. *et al.*, 2017
UBA547	UBA547	MGIIa	NCBI	ERX552262	1.87	no	78.93	0.80	0.00	Parks, D. H. *et al.*, 2017
UBA548	UBA548	MGIIa	NCBI	ERX552262	1.75	no	73.25	1.76	0.00	Parks, D. H. *et al.*, 2017
UBA549	UBA549	MGIIa	NCBI	ERX552262	1.85	no	80.02	0.00	0.00	Parks, D. H. *et al.*, 2017
UBA550	UBA550	MGIIa	NCBI	ERX552262	1.15	no	54.21	0.00	0.00	Parks, D. H. *et al.*, 2017
UBA551	UBA551	MGIIb	NCBI	ERX552262	1.19	no	66.93	0.00	0.00	Parks, D. H. *et al.*, 2017
UBA552	UBA552	MGIIb	NCBI	ERX552264	1.36	yes	72.00	0.80	0.00	Parks, D. H. *et al.*, 2017
UBA553	UBA553	MGIIa	NCBI	ERX552264	1.76	no	80.40	0.00	0.00	Parks, D. H. *et al.*, 2017
UBA554	UBA554	MGIIb	NCBI	ERX555951	1.06	no	59.87	0.00	0.00	Parks, D. H. *et al.*, 2017
UBA555	UBA555	MGIIb	NCBI	ERX555951	1.17	no	66.80	0.00	0.00	Parks, D. H. *et al.*, 2017
UBA556	UBA556	MGIIb	NCBI	SRX802189	1.34	no	68.11	0.80	0.00	Parks, D. H. *et al.*, 2017
UBA557	UBA557	MGIIb	NCBI	SRX802189	1.52	yes	84.53	0.00	0.00	Parks, D. H. *et al.*, 2017
UBA558	UBA558	MGIIb	NCBI	ERX555954	1.44	no	76.40	1.60	0.00	Parks, D. H. *et al.*, 2017
UBA559	UBA559	MGIIa	NCBI	ERX555954	1.73	no	68.51	0.86	0.00	Parks, D. H. *et al.*, 2017
UBA56	UBA56	MGIIb	NCBI	SRX1075082	1.23	no	63.31	0.00	0.00	Parks, D. H. *et al.*, 2017
UBA560	UBA560	MGIIb	NCBI	ERX555954	1.34	no	72.36	3.20	0.00	Parks, D. H. *et al.*, 2017
UBA561	UBA561	MGIIa	NCBI	ERX555954	1.95	no	76.27	0.00	0.00	Parks, D. H. *et al.*, 2017
UBA562	UBA562	MGIIa	NCBI	ERX555957	1.56	no	61.81	2.20	54.55	Parks, D. H. *et al.*, 2017
UBA563	UBA563	MGIIa	NCBI	ERX555957	1.93	no	66.87	3.20	50.00	Parks, D. H. *et al.*, 2017
UBA58	UBA58	MGIIb	NCBI	SRX1075082	1.58	no	84.62	0.80	0.00	Parks, D. H. *et al.*, 2017
UBA584	UBA584	MGIIb	NCBI	ERX556032	1.61	no	72.07	1.33	0.00	Parks, D. H. *et al.*, 2017
UBA59	UBA59	MGIIb	NCBI	SRX1075082	1.84	no	73.37	0.93	0.00	Parks, D. H. *et al.*, 2017
UBA595	UBA595	MGIIb	NCBI	ERX552258	1.05	no	59.16	0.80	0.00	Parks, D. H. *et al.*, 2017
UBA596	UBA596	MGIIa	NCBI	ERX552258	1.31	no	56.88	0.00	0.00	Parks, D. H. *et al.*, 2017
UBA597	UBA597	MGIIb	NCBI	ERX552257	1.33	no	73.60	0.00	0.00	Parks, D. H. *et al.*, 2017
UBA598	UBA598	MGIIb	NCBI	ERX552257	1.07	yes	56.53	0.00	0.00	Parks, D. H. *et al.*, 2017
UBA599	UBA599	MGIIa	NCBI	ERX552257	1.56	no	58.63	0.00	0.00	Parks, D. H. *et al.*, 2017
UBA60	UBA60	MGIIa	NCBI	SRX1075082	1.98	no	68.84	0.80	0.00	Parks, D. H. *et al.*, 2017
UBA600	UBA600	MGIIb	NCBI	ERX552256	1.37	no	70.80	0.00	0.00	Parks, D. H. *et al.*, 2017
UBA601	UBA601	MGIIb	NCBI	ERX552256	1.34	no	74.13	0.80	0.00	Parks, D. H. *et al.*, 2017
UBA602	UBA602	MGIIb	NCBI	ERX552256	1.83	no	75.54	0.00	0.00	Parks, D. H. *et al.*, 2017
UBA603	UBA603	MGIIb	NCBI	ERX552256	1.19	no	62.80	0.00	0.00	Parks, D. H. *et al.*, 2017
UBA604	UBA604	MGIIa	NCBI	ERX552254	1.69	no	69.86	0.00	0.00	Parks, D. H. *et al.*, 2017
UBA605	UBA605	MGIIb	NCBI	ERX552254	1.44	no	83.07	0.00	0.00	Parks, D. H. *et al.*, 2017
UBA606	UBA606	MGIIb	NCBI	ERX552254	1.16	no	59.91	0.86	50.00	Parks, D. H. *et al.*, 2017
UBA607	UBA607	MGIIb	NCBI	ERX552253	1.37	no	79.87	0.80	0.00	Parks, D. H. *et al.*, 2017
UBA608	UBA608	MGIIb	NCBI	ERX552255	1.17	no	65.65	0.80	0.00	Parks, D. H. *et al.*, 2017
UBA609	UBA609	MGIIa	NCBI	ERX552255	1.75	no	75.65	2.00	0.00	Parks, D. H. *et al.*, 2017
UBA610	UBA610	MGIIb	NCBI	ERX552252	1.44	no	82.67	0.29	0.00	Parks, D. H. *et al.*, 2017

UBA618	UBA618	MGIIa	NCBI	ERX555941	1.97	no	71.86	0.80	0.00	Parks, D. H. *et al.*, 2017
UBA619	UBA619	MGIIb	NCBI	ERX555947	1.37	no	72.80	0.00	0.00	Parks, D. H. *et al.*, 2017
UBA62	UBA62	MGIIb	NCBI	ERX289005	1.32	yes	77.16	0.40	100.00	Parks, D. H. *et al.*, 2017
UBA620	UBA620	MGIIa	NCBI	ERX555947	2.79	no	83.52	2.80	0.00	Parks, D. H. *et al.*, 2017
UBA621	UBA621	MGIIa	NCBI	ERX555945	1.70	no	66.89	1.20	0.00	Parks, D. H. *et al.*, 2017
UBA622	UBA622	MGIIb	NCBI	ERX555949	1.30	no	72.22	0.00	0.00	Parks, D. H. *et al.*, 2017
UBA623	UBA623	MGIIb	NCBI	ERX555949	1.22	no	58.75	0.80	0.00	Parks, D. H. *et al.*, 2017
UBA63	UBA63	MGIIa	NCBI	ERX289005	1.88	no	77.33	0.40	0.00	Parks, D. H. *et al.*, 2017
UBA66	UBA66	MGIIb	NCBI	ERX552239	1.32	no	71.07	0.80	0.00	Parks, D. H. *et al.*, 2017
UBA68	UBA68	MGIIa	NCBI	ERX552238	1.62	no	77.08	0.80	0.00	Parks, D. H. *et al.*, 2017
UBA69	UBA69	MGIIb	NCBI	ERX552238	1.48	yes	75.60	4.80	0.00	Parks, D. H. *et al.*, 2017
UBA82	UBA82	MGIIa	NCBI	ERX555961	1.62	no	72.23	1.52	9.09	Parks, D. H. *et al.*, 2017
UBA83	UBA83	MGIIa	NCBI	ERX555963	1.85	no	71.24	0.00	0.00	Parks, D. H. *et al.*, 2017
UBA84	UBA84	MGIIb	NCBI	ERX555963	1.30	no	56.91	0.00	0.00	Parks, D. H. *et al.*, 2017
UBA85	UBA85	MGIIa	NCBI	ERX555966	1.64	no	71.60	0.80	0.00	Parks, D. H. *et al.*, 2017
UBA86	UBA86	MGIIb	NCBI	ERX555966	1.28	no	69.16	0.80	0.00	Parks, D. H. *et al.*, 2017
UBA87	UBA87	MGIIa	NCBI	ERX555966	1.73	no	74.43	2.46	0.00	Parks, D. H. *et al.*, 2017
UBA88	UBA88	MGIIa	NCBI	ERX555967	1.87	no	81.97	0.00	0.00	Parks, D. H. *et al.*, 2017
UBA89	UBA89	MGIIb	NCBI	ERX555967	1.39	yes	78.90	0.80	100.00	Parks, D. H. *et al.*, 2017
UBA9	UBA9	MGIIb	NCBI	ERX556127	1.30	no	71.22	1.60	0.00	Parks, D. H. *et al.*, 2017
UBA90	UBA90	MGIIa	NCBI	ERX555967	1.65	no	74.75	0.80	0.00	Parks, D. H. *et al.*, 2017
UBA91	UBA91	MGIIa	NCBI	ERX555967	1.88	no	78.80	0.00	0.00	Parks, D. H. *et al.*, 2017
UBA98	UBA98	MGIIb	NCBI	ERX556035	1.34	no	68.53	0.80	100.00	Parks, D. H. *et al.*, 2017
UBA99	UBA99	MGIIb	NCBI	ERX556033	1.20	no	63.42	0.80	100.00	Parks, D. H. *et al.*, 2017

Supplementary Table 2. Information for analyzed genomes used in study, including clade and subclade assignment, completion stats (length, percent complete, percent contamination, percent strain heterogeneity), and source reference. Estimated completeness (%Comp); estimated contamination (%Contam); estimated strain heterogeneity (%Strain); percent GC (%G+C). All lengths are in Mbp. Approximate length determined by (Size × %Comp).

GenomeID	MGII Clade	Clade ID	Source	Accession ID	Size	16S rRNA present	%Comp	%Cont	%Strain	%G+C	Approximate Length
MGIIbin14	MGIIb	12	IMG	2651870037	1.39	no	71.2	0.8	100	39.61	1.95
MGIIbin15	MGIIb	n/a	IMG	2651870038	1.89	yes	83.2	1.6	0	37.49	2.27
MGIIbin17	MGIIa	n/a	IMG	2651870039	1.8	yes	81.9	0.1	50	41.76	2.2
MGIIbin18	MGIIb	16	IMG	2651870040	1.03	no	61.1	0.8	100	41.74	1.69
MG2-GG3puget	MGIIa	8	NCBI	AHCG00000000	2.06	yes	83.2	0	0	51.56	2.48
UBA62	MGIIb	17	NCBI	ERX289005	1.32	yes	77.16	0.4	100	46.79	1.71
UBA20	MGIIb	11	NCBI	ERX552241	1.4	no	76.4	0	0	48.64	1.83
UBA605	MGIIb	15	NCBI	ERX552254	1.44	no	83.07	0	0	37.49	1.73
UBA602	MGIIb	9	NCBI	ERX552256	1.83	no	75.54	0	0	48.97	2.42
UBA601	MGIIb	17	NCBI	ERX552256	1.34	no	74.13	0.8	0	47.66	1.81
UBA498	MGIIb	17	NCBI	ERX552274	1.26	no	72.13	0.13	100	46.83	1.75
UBA501	MGIIb	9	NCBI	ERX552275	1.51	no	71.6	1.6	50	48.43	2.11
UBA500	MGIIb	12	NCBI	ERX552275	1.5	no	84.93	0.8	0	40.58	1.77
UBA540	MGIIb	16	NCBI	ERX552281	1.1	no	55.57	0	0	51.98	1.98
UBA484	MGIIa	4	NCBI	ERX552297	2	no	83.33	3.6	0	62.57	2.4
UBA486	MGIIa	6	NCBI	ERX552297	1.8	no	76.36	0.8	0	58.04	2.36
UBA171	MGIIb	11	NCBI	ERX555907	1.34	no	74.4	0	0	49.19	1.8
UBA130	MGIIb	17	NCBI	ERX555912	1.35	no	80.13	0	0	47.71	1.68
UBA124	MGIIa	5	NCBI	ERX555914	1.88	no	80	0	0	48.43	2.35
UBA123	MGIIa	7	NCBI	ERX555914	1.87	yes	80.53	4	0	38.71	2.32
UBA125	MGIIb	10	NCBI	ERX555914	1.82	no	80.51	0.04	100	39.62	2.26
UBA128	MGIIa	7	NCBI	ERX555916	1.62	no	80.73	3.52	33.33	44.3	2.01
UBA126	MGIIb	12	NCBI	ERX555917	1.45	no	82.51	0.93	100	45.1	1.76
UBA121	MGIIb	17	NCBI	ERX555918	1.3	no	73.82	0.08	0	46.5	1.76
UBA120	MGIIa	3	NCBI	ERX555919	1.72	no	75.56	0.8	100	41.94	2.28
UBA196	MGIIb	n/a	NCBI	ERX555931	1.82	no	72.4	3.32	28.57	39.43	2.51
UBA193	MGIIb	12	NCBI	ERX555931	1.19	no	67.27	0	0	44.99	1.77
UBA200	MGIIb	16	NCBI	ERX555932	1.07	no	62	0	0	48.52	1.73
UBA622	MGIIb	14	NCBI	ERX555949	1.3	no	72.22	0	0	57.64	1.8
UBA623	MGIIb	16	NCBI	ERX555949	1.22	no	58.75	0.8	0	54.1	2.08
UBA559	MGIIa	4	NCBI	ERX555954	1.73	no	68.51	0.86	0	60.25	2.53
UBA561	MGIIa	6	NCBI	ERX555954	1.95	no	76.27	0	0	53.88	2.56
UBA558	MGIIb	11	NCBI	ERX555954	1.44	no	76.4	1.6	0	49.76	1.88
UBA560	MGIIb	17	NCBI	ERX555954	1.34	no	72.36	3.2	0	47.55	1.85
UBA82	MGIIa	5	NCBI	ERX555961	1.62	no	72.23	1.52	9.09	44.61	2.24
UBA83	MGIIa	4	NCBI	ERX555963	1.85	no	71.24	0	0	60.39	2.6
UBA84	MGIIb	10	NCBI	ERX555963	1.3	no	56.91	0	0	40.05	2.28
UBA85	MGIIa	3	NCBI	ERX555966	1.64	no	71.6	0.8	0	43.43	2.29
UBA90	MGIIa	7	NCBI	ERX555967	1.65	no	74.75	0.8	0	41.83	2.21
UBA88	MGIIa	8	NCBI	ERX555967	1.87	no	81.97	0	0	49.54	2.28
UBA91	MGIIa	8	NCBI	ERX555967	1.88	no	78.8	0	0	46.38	2.39
UBA89	MGIIb	15	NCBI	ERX555967	1.39	yes	78.9	0.8	100	37.73	1.76
UBA34	MGIIb	12	NCBI	ERX555973	1.4	no	75.88	2.4	60	53.05	1.85
UBA36	MGIIb	14	NCBI	ERX555978	1.34	no	69.72	1.2	100	37.18	1.92
UBA441	MGIIa	3	NCBI	ERX555984	1.7	no	75.61	2.66	9.09	41.69	2.25

UBA442	MGIIa	6	NCBI	ERX555984	1.76	no	77.84	0.04	100	54.93	2.26
UBA439	MGIIb	15	NCBI	ERX555987	1.39	no	81.73	0	0	37.96	1.7
UBA438	MGIIb	17	NCBI	ERX555987	1.28	no	72.93	0	0	52.36	1.76
UBA529	MGIIb	12	NCBI	ERX555990	1.37	no	76.4	0	0	45.49	1.79
UBA538	MGIIb	17	NCBI	ERX555999	1.2	no	68.27	0	0	46.68	1.76
UBA40	MGIIb	10	NCBI	ERX556005	1.8	no	78.67	1.64	0	48.7	2.29
UBA39	MGIIb	10	NCBI	ERX556005	1.58	no	64.94	1.33	33.33	40.36	2.43
UBA4	MGIIb	17	NCBI	ERX556017	0.93	no	55.47	0	0	51.68	1.68
UBA3	MGIIb	17	NCBI	ERX556017	0.95	no	52.1	0	0	51.33	1.82
UBA220	MGIIb	16	NCBI	ERX556028	1.31	no	79.36	1.6	0	48.44	1.65
UBA99	MGIIb	15	NCBI	ERX556033	1.2	no	63.42	0.8	100	43.84	1.89
UBA98	MGIIb	11	NCBI	ERX556035	1.34	no	68.53	0.8	100	49.69	1.96
UBA175	MGIIb	12	NCBI	ERX556040	1.27	no	70	0	0	52.34	1.81
UBA180	MGIIb	17	NCBI	ERX556054	1.35	no	69.8	2.4	0	51.77	1.93
UBA181	MGIIb	17	NCBI	ERX556054	1.33	no	77.41	0.8	0	46.8	1.72
UBA182	MGIIb	11	NCBI	ERX556056	1.37	no	73.2	0	0	50.11	1.87
UBA251	MGIIb	10	NCBI	ERX556060	1.76	no	81.27	1.6	50	48.53	2.17
UBA250	MGIIb	11	NCBI	ERX556060	1.26	no	75.2	0.8	0	49.5	1.68
UBA229	MGIIa	7	NCBI	ERX556094	1.72	no	75.29	1.6	0	44.77	2.28
UBA226	MGIIa	n/a	NCBI	ERX556098	1.62	no	71.07	0	0	46.87	2.28
UBA105	MGIIb	12	NCBI	ERX556101	1.35	no	73.28	2.04	25	45.47	1.84
UBA103	MGIIb	17	NCBI	ERX556101	1.31	no	77.61	0	0	46.95	1.69
UBA107	MGIIa	3	NCBI	ERX556103	1.68	no	69.73	0.97	0	43.78	2.41
UBA106	MGIIb	12	NCBI	ERX556103	1.08	no	64.13	0	0	43.51	1.68
UBA108	MGIIb	12	NCBI	ERX556103	0.91	no	57.4	0	0	45.35	1.59
UBA112	MGIIb	14	NCBI	ERX556105	0.86	no	55.45	0	0	50.39	1.55
UBA111	MGIIa	7	NCBI	ERX556106	1.43	no	59.25	0.8	0	44.18	2.41
UBA101	MGIIb	16	NCBI	ERX556108	1.12	no	67.73	2	33.33	51.51	1.65
UBA10	MGIIa	3	NCBI	ERX556126	1.71	no	75.09	0	0	44.91	2.28
UBA9	MGIIb	15	NCBI	ERX556127	1.3	no	71.22	1.6	0	38.02	1.83
UBA53	MGIIa	3	NCBI	ERX556130	1.73	yes	80.22	0	0	41.91	2.16
UBA54	MGIIb	16	NCBI	ERX556139	1.13	no	59.73	0	0	55.94	1.89
TARA-ANE-MAG-00030	MGIIa	1	figshare	figshare	1.65	no	80.4	2.86	20	34.37	2.05
TARA-RED-MAG-00055	MGIIa	2	figshare	figshare	1.82	no	79.6	0.06	0	42.7	2.29
TARA-ASW-MAG-00017	MGIIa	3	figshare	figshare	1.83	no	71.69	1.87	0	43.05	2.55
TARA-ASW-MAG-00011	MGIIa	3	figshare	figshare	1.96	no	83.2	0	0	42.52	2.36
TARA-ANE-MAG-00024	MGIIa	3	figshare	figshare	1.89	no	82.93	0	0	42.88	2.28
TARA-ANE-MAG-00027	MGIIa	4	figshare	figshare	1.92	no	79.6	0.8	0	60.91	2.41
TARA-ANE-MAG-00055	MGIIa	6	figshare	figshare	1.54	no	72.69	0	0	48.16	2.12
TARA-PSW-MAG-00056	MGIIa	6	figshare	figshare	1.86	no	80.8	1.6	100	55.89	2.3
TARA-MED-MAG-00060	MGIIa	6	figshare	figshare	1.62	no	77.14	1.82	80	54.17	2.1
TARA-PSW-MAG-00086	MGIIa	7	figshare	figshare	1.91	no	70	1.42	50	41.61	2.73
TARA-ASW-MAG-00019	MGIIa	7	figshare	figshare	1.51	no	66.67	0.84	25	41.78	2.26
TARA-PON-MAG-00033	MGIIa	8	figshare	figshare	1.84	no	75.73	0	0	46.09	2.43
TARA-RED-MAG-00049	MGIIb	n/a	figshare	figshare	1.57	no	74.56	0.96	50	51.02	2.11
TARA-ANE-MAG-00019	MGIIb	10	figshare	figshare	1.85	no	84.53	1.6	0	48.79	2.19
TARA-ANW-MAG-00045	MGIIb	10	figshare	figshare	1.63	no	66.53	0	0	41.78	2.45
TARA-PSW-MAG-00075	MGIIb	11	figshare	figshare	1.42	no	80.27	0	0	49.76	1.77
TARA-MED-MAG-00095	MGIIb	11	figshare	figshare	1.41	no	76.71	0	0	49.09	1.84
TARA-PSW-MAG-00057	MGIIb	11	figshare	figshare	1.44	no	76.4	0	0	50.05	1.88
TARA-ION-MAG-00033	MGIIb	13	figshare	figshare	1.51	no	83.27	0.93	50	35.95	1.81
TARA-ION-MAG-00034	MGIIb	14	figshare	figshare	1.32	no	70.09	0.8	0	49.76	1.88
TARA-ION-MAG-00040	MGIIb	14	figshare	figshare	1.2	no	64.13	0.92	33.33	50.41	1.87
TARA-ION-MAG-00044	MGIIb	14	figshare	figshare	1.27	no	74.42	0	0	46.35	1.71
MED710	MGIIb	14	figshare	figshare	1.04	no	59.74	0	0	51.39	1.74
TARA-RED-MAG-00062	MGIIb	15	figshare	figshare	1.27	no	74.22	0.4	0	38	1.71
TARA-PSE-MAG-00070	MGIIb	15	figshare	figshare	1.14	no	64.94	0.8	50	39.41	1.76
TARA-ANE-MAG-00052	MGIIb	16	figshare	figshare	1.25	no	68.9	0	0	51.59	1.81
TARA-PSW-MAG-00073	MGIIb	16	figshare	figshare	1.14	no	71.96	0.04	0	53.39	1.58
TARA-ANW-MAG-00046	MGIIb	17	figshare	figshare	1.3	no	75.78	0.8	100	51.8	1.72
TARA-ASW-MAG-00021	MGIIb	17	figshare	figshare	1.15	no	66.09	1.73	100	47.9	1.74
REDSEA-S03-B6	MGIIb	12	NCBI	LURP00000000	1.32	yes	64.47	2.3	62.5	45.47	2.05
REDSEA-S11-B3N4	MGIIb	14	NCBI	LURR00000000	1.3	yes	81.96	0	0	50.98	1.59
REDSEA-S30-B12	MGIIb	14	NCBI	LURV00000000	1.27	no	67.79	0.8	0	36.9	1.87
REDSEA-S40-B11N13	MGIIb	14	NCBI	LURX00000000	1.24	no	71.96	0	0	50.09	1.72
REDSEA-S41-B6	MGIIb	14	NCBI	LURY00000000	1.13	no	71.35	1.12	66.67	49.97	1.58
TMED97	MGIIb	13	NCBI	NHEY00000000	1.63	no	76.74	0.8	0	35.63	2.12
TMED99	MGIIa	8	NCBI	NHFA00000000	2.09	no	76.53	3.24	33.33	45.08	2.73
TMED129	MGIIb	13	NCBI	NHGE00000000	1.4	no	69.33	0	0	36.77	2.02
TMED132	MGIIa	7	ANVIO-REFINED	NHGH00000000	1.5	no	66.67	1.72	75	40.64	2.25
TMED141	MGIIa	4	NCBI	NHGQ00000000	2.36	no	67.53	4	0	53.7	3.49
MED-G37	MGIIb	12	NCBI	NTJQ00000000	1.28	yes	71.73	0	0	45.79	1.79
MED-G36	MGIIb	12	NCBI	NTJR00000000	0.89	no	56.4	0	0	43.65	1.57
MED-G35	MGIIb	14	NCBI	NTJS00000000	1.2	no	65.39	0	0	52.4	1.84
MED-G34	MGIIb	16	NCBI	NTJT00000000	1.03	no	59.35	0	0	53.64	1.73
MED-G33	MGIIb	9	NCBI	NTJU00000000	1.27	yes	57.26	0	0	48.15	2.22
CPC89	MGIIb	14	NCBI	NYST00000000	0.88	no	53.24	0	0	49.63	1.65
ARS96	MGIIb	12	NCBI	NYXO00000000	1.62	no	61.73	0.55	0	42.68	2.62
ARS91	MGIIb	11	NCBI	NYXY00000000	1.31	no	66.4	1.6	0	49.87	1.97
ARS60	MGIIa	8	NCBI	NYZH00000000	2.2	no	82.8	1.6	0	46.3	2.66
ARS58	MGIIb	14	NCBI	NYZK00000000	1.08	no	65.82	1.44	20	51.03	1.64
ARS47	MGIIb	17	NCBI	NYZV00000000	1.17	no	58.4	0	0	47.59	2
ARS1134	MGIIb	15	NCBI	NZDA00000000	1.36	no	73.14	0.8	50	37.92	1.86
MED743	MGIIb	14	NCBI	NZEY00000000	1.94	no	82.93	4	0	40.84	2.34

MED705	MGIIa	6	NCBI	NZFV00000000	1.81	no	74.53	0	0	48.22	2.43
MED678	MGIIa	3	NCBI	NZGQ00000000	1.62	no	56.74	0.8	100	42.32	2.86
IN961	MGIIa	1	NCBI	NZKH00000000	1.5	no	72.4	0.8	100	34.75	2.07
IN46	MGIIa	6	NCBI	NZLA00000000	1.61	no	63.25	1.6	0	59.47	2.55
IN37	MGIIa	2	NCBI	NZLK00000000	1.72	no	71.64	0.93	50	42.58	2.4
IN35	MGIIa	3	NCBI	NZLM00000000	1.98	no	84.13	2.8	50	42.81	2.35
IN34	MGIIb	17	NCBI	NZLN00000000	1.12	no	56.14	1.33	66.67	47.55	2
IN1222	MGIIa	4	NCBI	NZMM00000000	1.71	no	69.73	2.4	66.67	62.58	2.45
IN1219	MGIIa	6	NCBI	NZMN00000000	1.6	no	67.73	0	0	58.62	2.36
EAC710	MGIIa	2	NCBI	NZOE00000000	1.83	no	76.8	2.4	33.33	43.07	2.38
EAC40	MGIIb	17	NCBI	NZRH00000000	1.32	no	69.16	1.07	50	47.13	1.91
EAC1768	MGIIb	10	NCBI	NZSV00000000	1.84	no	72.67	4	50	46.88	2.53
NAT86	MGIIb	15	NCBI	NZUM00000000	1.08	no	54.33	1.6	0	38.06	1.99
NAT37	MGIIa	7	NCBI	NZXL00000000	1.6	no	65.01	0.8	100	39.8	2.46
NAT36	MGIIb	9	NCBI	NZXM00000000	1.39	no	58.45	1.26	33.33	47.45	2.38
NAT302	MGIIb	13	NCBI	NZXS00000000	1.41	no	60.35	3.73	100	35.93	2.34
NAT279	MGIIa	3	NCBI	NZYG00000000	1.34	no	53.57	3.12	50	43.84	2.5
NAT256	MGIIa	5	NCBI	NZZD00000000	1.24	no	51.48	2.4	100	44.06	2.41
NAT234	MGIIb	14	NCBI	NZZZ00000000	1.36	no	65.69	4	50	58.82	2.07
NAT11	MGIIa	2	NCBI	PABV00000000	1.91	no	78.93	0.93	0	41.54	2.42
NAT150	MGIIb	10	NCBI	PADI00000000	1.59	no	63.64	1.6	100	39.83	2.5
MED921	MGIIb	10	ANVIO-REFINED	PADP00000000	1.67	no	74.93	1.6	100	49.06	2.23
MED870	MGIIa	3	NCBI	PAEE00000000	1.61	no	74.13	2.4	100	43.62	2.17
MED853	MGIIa	5	ANVIO-REFINED	PAEM00000000	1.91	no	81.33	3.2	75	46.96	2.35
MED846	MGIIb	15	NCBI	PAET00000000	1.33	no	74.27	3.36	90	38.05	1.79
MED770	MGIIb	17	NCBI	PAGY00000000	1.22	no	64.76	1.87	33.33	47.07	1.88
SAT95	MGIIa	5	NCBI	PAJM00000000	1.89	no	81.6	0.8	100	48.06	2.32
SAT38	MGIIa	3	NCBI	PALS00000000	1.89	no	80.13	3.07	83.33	41.42	2.36
SAT2781	MGIIa	8	NCBI	PANB00000000	1.5	no	64.27	3.73	100	50.25	2.33
SAT2681	MGIIb	12	NCBI	PANM00000000	1.14	no	62.78	0.8	0	43.51	1.82
SAT218	MGIIb	10	NCBI	PAOK00000000	1.64	no	67.27	1.6	100	39.77	2.44
SP175	MGIIb	10	NCBI	PAOZ00000000	1.77	no	62.67	1.6	0	39.5	2.82
SP143	MGIIa	3	NCBI	PAQE00000000	1.53	no	63.33	1.6	100	44.03	2.42
SAT165	MGIIb	10	NCBI	PATC00000000	1.7	no	77.69	0.8	100	46.41	2.19
SAT157	MGIIa	4	NCBI	PATU00000000	2.48	no	81.2	4.27	66.67	61.51	3.05
SAT1553	MGIIb	13	NCBI	PATZ00000000	1.59	no	76.27	4	33.33	37.37	2.08
SAT1510	MGIIb	11	NCBI	PAUH00000000	1.55	no	75.02	4	37.5	49.62	2.07
SAT1485	MGIIa	3	ANVIO-REFINED	PAUS00000000	1.27	no	56.4	3.2	60	43.54	2.25
SAT1460	MGIIb	14	NCBI	PAUZ00000000	1.09	no	59.61	2.4	33.33	58.19	1.83
SAT1329	MGIIa	6	NCBI	PAWY00000000	1.63	no	77.06	2.4	100	54.87	2.12
SAT122	MGIIa	5	NCBI	PAXR00000000	1.9	no	70.81	1.6	0	47.24	2.68
SAT116	MGIIb	15	NCBI	PAXY00000000	1.29	no	70.34	1.6	50	37.82	1.83
SAT102	MGIIb	15	NCBI	PAYN00000000	1.03	no	57.73	0	0	37.66	1.78
SP88	MGIIb	14	NCBI	PAYZ00000000	1.58	no	79.07	0.8	0	49.37	2
SP42	MGIIb	11	NCBI	PBBV00000000	1.21	no	66.09	0	0	48.03	1.83
SP338	MGIIb	10	NCBI	PBDV00000000	1.58	no	67.02	0	0	41.53	2.36
SP3107	MGIIb	16	NCBI	PBFF00000000	1.3	no	73.78	3.2	83.33	48.81	1.76
SP3093	MGIIb	14	NCBI	PBFO00000000	1.17	no	58.67	3.2	25	48.59	1.99
SP3092	MGIIb	14	NCBI	PBFP00000000	1.36	no	67.2	2.53	28.57	49.73	2.02
SP3088	MGIIb	9	NCBI	PBFT00000000	1.44	no	58.74	4	11.11	46.91	2.45
SP3068	MGIIb	14	NCBI	PBGH00000000	1.56	no	76.81	4.8	100	46.54	2.03
SP3067	MGIIb	14	NCBI	PBGI00000000	1.14	no	58.47	4.8	100	51.46	1.95
SP3063	MGIIb	17	ANVIO-REFINED	PBGL00000000	1.48	no	86.4	2.4	66.67	51.2	1.71
SP3054	MGIIa	3	NCBI	PBGS00000000	2.03	no	74.93	4	66.67	43.03	2.71
SP3051	MGIIb	10	NCBI	PBGT00000000	1.65	no	60.99	2.4	66.67	40.76	2.71
SP303	MGIIa	2	NCBI	PBHM00000000	1.74	no	73.2	1.6	50	42.75	2.38
SP3027	MGIIb	9	NCBI	PBHP00000000	1.18	no	54.22	4.59	71.43	47.9	2.18
SP3025	MGIIa	n/a	NCBI	PBHQ00000000	1.89	no	74.67	3.36	66.67	46.05	2.53
SP3024	MGIIa	1	NCBI	PBHR00000000	1.82	no	77.73	3.26	20	34.74	2.34
SP3014	MGIIb	13	NCBI	PBHZ00000000	1.67	no	75.33	2.4	66.67	36.04	2.22
SP3007	MGIIb	17	NCBI	PBIF00000000	1.09	yes	54.22	3.6	80	46.89	2.01
SP231	MGIIb	16	NCBI	PBLT00000000	0.81	no	52.42	1.6	50	58.74	1.55
SP210	MGIIb	9	NCBI	PBMO00000000	1.64	no	77.16	1.6	50	44.71	2.13
SP192	MGIIb	14	NCBI	PBNI00000000	0.87	no	52.44	0	0	50.94	1.66
RS392	MGIIb	14	NCBI	PBOL00000000	1.41	no	76.4	0.8	0	52.4	1.85
RS354	MGIIb	14	NCBI	PBPQ00000000	1.25	no	68.24	1.6	50	51.3	1.83
RS348	MGIIb	9	NCBI	PBPV00000000	1.84	no	78.51	1.92	100	49.5	2.34
NP965	MGIIb	17	NCBI	PBRB00000000	1.42	no	75	4.8	83.33	47.28	1.89
NP84	MGIIb	17	NCBI	PBSO00000000	1.24	no	69.2	0	0	52.17	1.79
NP83	MGIIa	5	NCBI	PBSP00000000	1.48	no	60.83	0.13	100	48.03	2.43
NP65	MGIIa	6	NCBI	PBTH00000000	1.73	no	79.4	0	0	48.12	2.18
RS462	MGIIb	14	NCBI	PBUH00000000	1.26	no	63.6	3.2	0	51.85	1.98
RS443	MGIIb	14	NCBI	PBUW00000000	1.13	no	66	4.29	75	52.22	1.71
RS440	MGIIa	6	NCBI	PBUZ00000000	1.84	no	74.53	3.2	100	54.86	2.47
RS814	MGIIb	16	NCBI	PBWO00000000	1.38	no	74.56	2.74	72.73	50.2	1.85
RS604	MGIIa	3	NCBI	PBXF00000000	1.88	no	75.6	4.11	52.63	41.44	2.49
NP1521	MGIIb	17	NCBI	PBZE00000000	1.28	no	75.58	0.8	0	47.72	1.69
NP147	MGIIa	5	NCBI	PBZN00000000	1.33	no	50.74	0	0	48.74	2.62
NP1344	MGIIb	13	NCBI	PCAI00000000	1.12	no	60.39	0.27	0	36.82	1.85
Guaymas26	MGIIa	n/a	Personal comm	Personal comm	1.75	no	77.73	0.8	0	43.48	2.25
Guaymas28	MGIIa	3	Personal comm	Personal comm	1.92	no	85.33	2.86	0	45.19	2.25
Guaymas21	MGIIb	n/a	Personal comm	Personal comm	1.5	no	80	2.4	33.33	56.27	1.88
Guaymas23	MGIIb	14	Personal comm	Personal comm	1.63	no	86	2.4	0	50.54	1.9
Guaymas29	MGIIb	15	Personal comm	Personal comm	1.6	no	78.76	2.78	83.33	43.84	2.03

Cayman51-deep	MGIIb	16	NCBI	PSPG00000000	1.48	yes	84.67	0	0	60.84	1.75
Guaymas22	MGIIb	16	Personal comm	Personal comm	1.45	no	81.56	0.64	25	55.54	1.78
UBA242	MGIIa	8	NCBI	SRX1050770	1.81	yes	82.4	0	0	41.6	2.2
UBA60	MGIIa	n/a	NCBI	SRX1075082	1.98	no	68.84	0.8	0	53.1	2.88
UBA59	MGIIb	n/a	NCBI	SRX1075082	1.84	no	73.37	0.93	0	56.79	2.51
UBA528	MGIIb	16	NCBI	SRX147858	1.11	no	61.92	0.93	25	59.47	1.79
UBA461	MGIIa	5	NCBI	SRX514547	1.88	no	83.42	0	0	45.72	2.25
UBA464	MGIIb	14	NCBI	SRX514549	1.23	no	70.76	0	0	46.99	1.74
UBA465	MGIIb	17	NCBI	SRX514549	1.36	no	79.73	3.2	33.33	47.44	1.71
UBA433	MGIIa	8	NCBI	SRX672291	1.84	no	80.02	1.6	50	51.53	2.3
UBA256	MGIIa	6	NCBI	SRX802076	1.79	yes	84.13	0	0	51.38	2.13
UBA258	MGIIa	8	NCBI	SRX802076	1.88	no	73.07	4.53	37.5	51.48	2.57
UBA259	MGIIa	8	NCBI	SRX802076	1.17	no	67.47	0	0	48.78	1.73
UBA260	MGIIb	15	NCBI	SRX802076	1.45	no	73.26	3.07	60	38.39	1.98
UBA255	MGIIa	6	NCBI	SRX802077	1.94	yes	83.47	0	0	50.11	2.32
UBA557	MGIIb	13	NCBI	SRX802189	1.52	yes	84.53	0	0	35.72	1.8
EAC30	MGIIa	1	NCBI	NZRR00000000	1.71	no	77.33	0.80	100.00	43.26	2.21
RS406	MGIIa	7	NCBI	PBWC00000000	2.03	no	80.93	1.60	100.00	41.92	2.51
SP100	MGIIb	9	NCBI	PARS00000000	1.46	no	59.42	0.80	0.00	40.54	2.46
SP3115	MGIIb	16	NCBI	PBEZ00000000	0.87	no	53.6	4	22.22	41.84	1.62
TARA-ANE-MAG-00063	MGIIb	17	figshare	figshare	1.14	no	60.82	0.00	0.00	44.42	1.87
TARA-ANW-MAG-00024	MGIIa	5	figshare	figshare	1.81	no	80	0.04	100	39.73	2.26
TARA-ANW-MAG-00033	MGIIb	17	figshare	figshare	1.32	no	75.34	0	0	44.36	1.75
TARA-ASE-MAG-00018	MGIIa	6	figshare	figshare	1.43	no	64.51	0	0	38.34	2.22
TARA-ASW-MAG-00013	MGIIb	17	figshare	figshare	1.24	no	70.93	0	0	44.31	1.75
TARA-IOS-MAG-00030	MGIIb	12	figshare	figshare	1.23	no	73.47	0	0	42.59	1.67
TARA-MED-MAG-00079	MGIIa	5	figshare	figshare	1.71	no	73.33	0	0	39.21	2.33
TARA-MED-MAG-00080	MGIIb	10	figshare	figshare	1.77	no	78.4	0.8	0	40.64	2.26
TARA-PON-MAG-00047	MGIIb	17	figshare	figshare	1.22	no	61.69	3.66	66.67	44.01	1.98
TARA-PSW-MAG-00102	MGIIa	6	figshare	figshare	1.51	no	63.21	0.4	100	37.36	2.39
UBA110	MGIIa	5	NCBI	ERX556103	1.81	no	76.31	0	0	39.22	2.37
UBA221	MGIIb	9	NCBI	ERX556028	1.47	no	56.4	0	0	40.41	2.61
UBA530	MGIIb	9	NCBI	ERX555990	1.62	no	71.42	0.8	0	40.48	2.27
UBA548	MGIIa	7	NCBI	ERX552262	1.75	no	73.25	1.76	0	39.55	2.39
UBA550	MGIIa	3	NCBI	ERX552262	1.15	no	54.21	0	0	40.83	2.12
UBA58	MGIIb	n/a	NCBI	SRX1075082	1.58	no	84.62	0.80	0.00	39.27	1.87
UBA620	MGIIa	1	NCBI	ERX555947	2.79	no	83.52	2.80	0.00	44.45	3.34

Supplementary Table 3. All genomes with identified archaeal flagellum components, including the number of identified components and a prediction of if a full operon is present. Genomes from Tully *et al*. (2018) used to visualize the putative operon have NCBI contig accession and operon protein IDs listed.

GenomeID	flaJ present?	flaI present?	No. of archaeal flagellin	Putative Operon Detected	MGII Clade	Clade ID	NCBI Contig Accession ID	Proposed Operon (protein ID)	Operon Start (bp)	Operon End (bp)	Orientation
TARA-ANE-MAG-00030	Y	Y	1	Y	MGIIa	1	n.a.	n.a.	n.a.	n.a.	n.a.
UBA620	Y	Y	3	Y	MGIIa	1	n.a.	n.a.	n.a.	n.a.	n.a.
IN961	Y	Y	2	Y	MGIIa	1	NZKH01000090	MAK05506-12	2	7407	FORWARD
EAC30	Y	Y	3	Y	MGIIa	1	NZRR01000031	MAO70188-94	1	7386	FORWARD
SP3024	Y	Y	2	Y	MGIIa	1	PBHR01000008	MBJ99730-6	1	14007	REVERSE
TARA-RED-MAG-00055	Y	Y	1	Y	MGIIa	2	n.a.	n.a.	n.a.	n.a.	n.a.
IN37	Y	Y	1	Y	MGIIa	2	NZLK01000058	MAK41862-7	14983	31	REVERSE
EAC710	Y	Y	3	Y	MGIIa	2	NZOE01000070	MAN07532-41	1	12400	FORWARD
NAT11	Y	Y	1	Y	MGIIa	2	PABV01000030	MAT86057-61	28410	33545	REVERSE
SP303	Y	Y	3	Y	MGIIa	2	PBHM01000005	MBK54526-36	6152	20664	FORWARD
UBA120	Y	Y	2	N	MGIIa	3	n.a.	n.a.	n.a.	n.a.	n.a.
UBA85	Y	Y	3	Y	MGIIa	3	n.a.	n.a.	n.a.	n.a.	n.a.
UBA441	N	Y	3	Y	MGIIa	3	n.a.	n.a.	n.a.	n.a.	n.a.
UBA10	Y	Y	2	N	MGIIa	3	n.a.	n.a.	n.a.	n.a.	n.a.
UBA53	Y	Y	3	Y	MGIIa	3	n.a.	n.a.	n.a.	n.a.	n.a.
TARA-ASW-MAG-00017	Y	Y	2	Y	MGIIa	3	n.a.	n.a.	n.a.	n.a.	n.a.
TARA-ASW-MAG-00011	Y	Y	3	Y	MGIIa	3	n.a.	n.a.	n.a.	n.a.	n.a.
TARA-ANE-MAG-00024	Y	Y	3	Y	MGIIa	3	n.a.	n.a.	n.a.	n.a.	n.a.
SAT1485	N	Y	0	N	MGIIa	3	n.a.	n.a.	n.a.	n.a.	n.a.
Guaymas28	Y	Y	1	Y	MGIIa	3	n.a.	n.a.	n.a.	n.a.	n.a.
UBA550	Y	Y	1	Y	MGIIa	3	n.a.	n.a.	n.a.	n.a.	n.a.
MED678	N	Y	3	Y	MGIIa	3	NZGQ01000030	MAI04448-57	1	10852	FORWARD
IN35	Y	Y	3	Y	MGIIa	3	NZLM01000078	MAK97313-23	5366	19273	REVERSE
NAT279	Y	Y	3	Y	MGIIa	3	NZYG01000005	MAS31401-11	5331	19166	FORWARD
MED870	Y	Y	3	Y	MGIIa	3	PAEE01000046	MAV03405-14	1	12815	FORWARD
SAT38	Y	Y	3	Y	MGIIa	3	PALS01000035	MAY13247-57	30075	43960	REVERSE
SP143	Y	Y	3	Y	MGIIa	3	PAQE01000022	MBB70227-37	21690	35575	REVERSE
SP3054	Y	Y	3	Y	MGIIa	3	PBGS01000033	MBJ64265-73	167	8665	FORWARD
RS604	Y	Y	3	Y	MGIIa	3	PBXF01000047	MBS73948-57	8891	18510	REVERSE
UBA484	Y	Y	3	N	MGIIa	4	n.a.	n.a.	n.a.	n.a.	n.a.
UBA559	Y	Y	3	Y	MGIIa	4	n.a.	n.a.	n.a.	n.a.	n.a.
UBA83	N	Y	3	N	MGIIa	4	n.a.	n.a.	n.a.	n.a.	n.a.
TARA-ANE-MAG-00027	Y	Y	3	Y	MGIIa	4	n.a.	n.a.	n.a.	n.a.	n.a.
TMED141	Y	N	3	Y	MGIIa	4	NHGQ01000037	OUV95478-87	29050	42894	REVERSE
IN1222	Y	Y	3	Y	MGIIa	4	NZMM01000004	MAL51079-87	3682	11731	REVERSE

SAT157	Y	Y	3	Y	MGIIa	4	PATU01000055	MBD40710-9	27792	39858	REVERSE
MED853	Y	Y	3	Y	MGIIa	5	ANVIO	UPDATED	n.a.	n.a.	n.a.
UBA124	Y	Y	3	Y	MGIIa	5	n.a.	n.a.	n.a.	n.a.	n.a.
UBA82	Y	Y	3	N	MGIIa	5	n.a.	n.a.	n.a.	n.a.	n.a.
NAT256	Y	Y	0	N	MGIIa	5	n.a.	n.a.	n.a.	n.a.	n.a.
NP83	N	Y	1	N	MGIIa	5	n.a.	n.a.	n.a.	n.a.	n.a.
UBA461	Y	Y	2	N	MGIIa	5	n.a.	n.a.	n.a.	n.a.	n.a.
TARA-ANW-MAG-00024	Y	Y	3	N	MGIIa	5	n.a.	n.a.	n.a.	n.a.	n.a.
TARA-MED-MAG-00079	Y	Y	3	Y	MGIIa	5	n.a.	n.a.	n.a.	n.a.	n.a.
UBA110	Y	Y	3	Y	MGIIa	5	n.a.	n.a.	n.a.	n.a.	n.a.
SAT95	Y	Y	3	Y	MGIIa	5	PAJM01000030	MAY05111-22	39983	54291	FORWARD
SAT122	Y	Y	3	Y	MGIIa	5	PAXR01000050	MBF55834-45	53518	67937	REVERSE
UBA486	Y	Y	2	N	MGIIa	6	n.a.	n.a.	n.a.	n.a.	n.a.
UBA561	Y	Y	2	N	MGIIa	6	n.a.	n.a.	n.a.	n.a.	n.a.
UBA442	Y	Y	3	N	MGIIa	6	n.a.	n.a.	n.a.	n.a.	n.a.
TARA-ANE-MAG-00055	Y	Y	2	N	MGIIa	6	n.a.	n.a.	n.a.	n.a.	n.a.
TARA-PSW-MAG-00056	Y	Y	1	Y	MGIIa	6	n.a.	n.a.	n.a.	n.a.	n.a.
TARA-MED-MAG-00060	Y	Y	3	N	MGIIa	6	n.a.	n.a.	n.a.	n.a.	n.a.
IN1219	Y	Y	0	N	MGIIa	6	n.a.	n.a.	n.a.	n.a.	n.a.
RS440	N	Y	2	N	MGIIa	6	n.a.	n.a.	n.a.	n.a.	n.a.
UBA256	Y	Y	3	Y	MGIIa	6	n.a.	n.a.	n.a.	n.a.	n.a.
UBA255	Y	Y	2	Y	MGIIa	6	n.a.	n.a.	n.a.	n.a.	n.a.
TARA-ASE-MAG-00018	Y	Y	0	N	MGIIa	6	n.a.	n.a.	n.a.	n.a.	n.a.
TARA-PSW-MAG-00102	Y	Y	1	N	MGIIa	6	n.a.	n.a.	n.a.	n.a.	n.a.
MED705	Y	Y	2	Y	MGIIa	6	NZFV01000030	MAJ01420-31	54305	68616	REVERSE
IN46	N	Y	3	Y	MGIIa	6	NZLA01000044	MAK84468-76	1	9143	FORWARD
SAT1329	Y	Y	1	Y	MGIIa	6	PAWY01000021	MBE54355-61	1	6364	FORWARD
NP65	Y	Y	2	Y	MGIIa	6	PBTH01000053	MBQ70935-46	20079	34390	REVERSE
UBA123	Y	Y	2	Y	MGIIa	7	n.a.	n.a.	n.a.	n.a.	n.a.
UBA128	Y	Y	3	N	MGIIa	7	n.a.	n.a.	n.a.	n.a.	n.a.
UBA90	Y	Y	3	Y	MGIIa	7	n.a.	n.a.	n.a.	n.a.	n.a.
UBA229	Y	Y	3	Y	MGIIa	7	n.a.	n.a.	n.a.	n.a.	n.a.
UBA111	Y	Y	0	N	MGIIa	7	n.a.	n.a.	n.a.	n.a.	n.a.
TARA-PSW-MAG-00086	Y	Y	0	N	MGIIa	7	n.a.	n.a.	n.a.	n.a.	n.a.
TARA-ASW-MAG-00019	Y	Y	3	Y	MGIIa	7	n.a.	n.a.	n.a.	n.a.	n.a.
TMED132	Y	Y	0	N	MGIIa	7	n.a.	n.a.	n.a.	n.a.	n.a.
UBA548	Y	Y	3	N	MGIIa	7	n.a.	n.a.	n.a.	n.a.	n.a.
NAT37	Y	Y	3	Y	MGIIa	7	NZXL01000015	MAR93637-48	6024	20510	REVERSE
RS406	Y	Y	2	Y	MGIIa	7	PBWC01000025	MBR95553-60	14420	21807	REVERSE
MG2-GG3puget	Y	Y	2	Y	MGIIa	8	n.a.	n.a.	n.a.	n.a.	n.a.
UBA88	Y	Y	3	Y	MGIIa	8	n.a.	n.a.	n.a.	n.a.	n.a.
UBA91	Y	Y	3	Y	MGIIa	8	n.a.	n.a.	n.a.	n.a.	n.a.
TARA-PON-MAG-00033	Y	Y	3	Y	MGIIa	8	n.a.	n.a.	n.a.	n.a.	n.a.
UBA242	N	Y	3	Y	MGIIa	8	n.a.	n.a.	n.a.	n.a.	n.a.
UBA433	Y	Y	3	Y	MGIIa	8	n.a.	n.a.	n.a.	n.a.	n.a.
UBA258	Y	Y	2	N	MGIIa	8	n.a.	n.a.	n.a.	n.a.	n.a.
TMED99	Y	Y	3	Y	MGIIa	8	NHFA01000044	OUV16342-52	17030	31609	FORWARD
ARS60	Y	Y	3	Y	MGIIa	8	NYZH01000002	MAE78245-56	43385	58034	REVERSE
SAT2781	Y	Y	2	Y	MGIIa	8	PANB01000005	MAZ42588-98	71033	84957	FORWARD
UBA501	Y	Y	0	N	MGIIb	9	n.a.	n.a.	n.a.	n.a.	n.a.
MED-G33	Y	Y	2	Y	MGIIb	9	n.a.	n.a.	n.a.	n.a.	n.a.
NAT36	Y	Y	0	N	MGIIb	9	n.a.	n.a.	n.a.	n.a.	n.a.
SP3088	Y	Y	0	N	MGIIb	9	n.a.	n.a.	n.a.	n.a.	n.a.
UBA221	Y	Y	2	Y	MGIIb	9	n.a.	n.a.	n.a.	n.a.	n.a.
UBA530	N	Y	2	Y	MGIIb	9	n.a.	n.a.	n.a.	n.a.	n.a.
SP100	N	Y	2	Y	MGIIb	9	PARS01000019	MBC28847-58	1	16293	REVERSE
SP210	Y	Y	2	Y	MGIIb	9	PBMO01000024	MBN17249-64	74408	41797	REVERSE
RS348	Y	Y	2	Y	MGIIb	9	PBPV01000050	MBN74945-58	1	22635	REVERSE
MED921	Y	Y	2	Y	MGIIb	10	ANVIO	UPDATED	n.a.	n.a.	n.a.
UBA125	Y	Y	2	Y	MGIIb	10	n.a.	n.a.	n.a.	n.a.	n.a.
UBA84	Y	Y	0	N	MGIIb	10	n.a.	n.a.	n.a.	n.a.	n.a.
UBA40	Y	Y	2	Y	MGIIb	10	n.a.	n.a.	n.a.	n.a.	n.a.
UBA39	Y	Y	0	N	MGIIb	10	n.a.	n.a.	n.a.	n.a.	n.a.
UBA251	Y	Y	2	N	MGIIb	10	n.a.	n.a.	n.a.	n.a.	n.a.
TARA-ANE-MAG-00019	Y	Y	0	N	MGIIb	10	n.a.	n.a.	n.a.	n.a.	n.a.
TARA-ANW-MAG-00045	Y	Y	2	Y	MGIIb	10	n.a.	n.a.	n.a.	n.a.	n.a.
TARA-MED-MAG-00080	Y	Y	2	Y	MGIIb	10	n.a.	n.a.	n.a.	n.a.	n.a.
EAC1768	Y	Y	1	Y	MGIIb	10	NZSV01000017	MAP42503-9	4892	11194	REVERSE
NAT150	Y	Y	1	Y	MGIIb	10	PADI01000008	MAV06397-407	4071	17800	FORWARD
SAT218	Y	Y	2	Y	MGIIb	10	PAOK01000029	MBA40910-20	12441	26174	FORWARD
SP175	Y	Y	2	Y	MGIIb	10	PAOZ01000037	MBA46027-36	18981	31629	FORWARD
SAT165	N	Y	2	Y	MGIIb	10	PATC01000004	MBD18126-34	5580	18326	FORWARD
SP338	Y	Y	2	Y	MGIIb	10	PBDV01000009	MBI31325-34	24627	37290	FORWARD
UBA175	N	Y	1	Y	MGIIb	12	n.a.	n.a.	n.a.	n.a.	n.a.
SP3014	N	Y	1	N	MGIIb	13	n.a.	n.a.	n.a.	n.a.	n.a.
UBA557	N	Y	3	Y	MGIIb	13	n.a.	n.a.	n.a.	n.a.	n.a.
MED710	Y	Y	3	Y	MGIIb	14	MISSING	RECORD	n.a.	n.a.	n.a.
UBA622	Y	Y	0	N	MGIIb	14	n.a.	n.a.	n.a.	n.a.	n.a.
UBA36	Y	Y	1	N	MGIIb	14	n.a.	n.a.	n.a.	n.a.	n.a.
TARA-ION-MAG-00034	Y	Y	1	Y	MGIIb	14	n.a.	n.a.	n.a.	n.a.	n.a.
TARA-ION-MAG-00040	Y	Y	0	N	MGIIb	14	n.a.	n.a.	n.a.	n.a.	n.a.
TARA-ION-MAG-00044	Y	Y	1	Y	MGIIb	14	n.a.	n.a.	n.a.	n.a.	n.a.
REDSEA-S11-B3N4	Y	Y	1	Y	MGIIb	14	n.a.	n.a.	n.a.	n.a.	n.a.
REDSEA-S30-B12	N	Y	1	N	MGIIb	14	n.a.	n.a.	n.a.	n.a.	n.a.

REDSEA-S40-B11N13	Y	Y	1	Y	MGIIb	14	n.a.	n.a.	n.a.	n.a.	n.a.
REDSEA-S41-B6	Y	Y	0	N	MGIIb	14	n.a.	n.a.	n.a.	n.a.	n.a.
MED-G35	Y	Y	3	Y	MGIIb	14	n.a.	n.a.	n.a.	n.a.	n.a.
RS392	N	N	1	N	MGIIb	14	n.a.	n.a.	n.a.	n.a.	n.a.
RS354	Y	Y	0	N	MGIIb	14	n.a.	n.a.	n.a.	n.a.	n.a.
Guaymas23	Y	Y	0	N	MGIIb	14	n.a.	n.a.	n.a.	n.a.	n.a.
UBA464	Y	Y	2	N	MGIIb	14	n.a.	n.a.	n.a.	n.a.	n.a.
ARS58	Y	Y	3	Y	MGIIb	14	NYZK01000014	MAF19325-33	1	6838	REVERSE
MED743	Y	Y	2	Y	MGIIb	14	NZEY01000035	MAH98781-90	121239	135532	REVERSE
SP88	Y	Y	3	Y	MGIIb	14	PAYZ01000018	MBF93195-205	37613	52510	FORWARD
SP3093	Y	Y	3	Y	MGIIb	14	PBFO01000010	MBI87721-31	52047	66950	REVERSE
SP3092	Y	Y	0	Y	MGIIb	14	PBFP01000025	MBJ17679-86	1	7583	FORWARD
SP3069	Y	Y	0	Y	MGIIb	14	PBGH01000018	MBJ29465-71	172	7495	FORWARD
SP3067	Y	Y	3	Y	MGIIb	14	PBGI01000033	MBJ08384-92	7788	16479	REVERSE
RS462	Y	Y	1	Y	MGIIb	14	PBUH01000033	MBR41269-74	17	5357	FORWARD
RS443	Y	Y	3	Y	MGIIb	14	PBUW01000046	MBR79722-31	14046	29375	FORWARD
UBA602	Y	Y	2	Y	MGIIa	null	n.a.	n.a.	n.a.	n.a.	n.a.
UBA196	Y	Y	0	N	MGIIb	null	n.a.	n.a.	n.a.	n.a.	n.a.
UBA226	Y	Y	3	N	MGIIa	null	n.a.	n.a.	n.a.	n.a.	n.a.
TARA-RED-MAG-00049	Y	Y	0	N	MGIIb	null	n.a.	n.a.	n.a.	n.a.	n.a.
SP3025	N	Y	0	N	MGIIa	null	n.a.	n.a.	n.a.	n.a.	n.a.
Guaymas26	Y	Y	3	Y	MGIIa	null	n.a.	n.a.	n.a.	n.a.	n.a.
UBA60	N	Y	1	N	MGIIa	null	n.a.	n.a.	n.a.	n.a.	n.a.
UBA59	Y	Y	0	N	MGIIa	null	n.a.	n.a.	n.a.	n.a.	n.a.
UBA58	N	Y	0	N	MGIIb	null	n.a.	n.a.	n.a.	n.a.	n.a.

Supplementary Table 4. A breakdown of the peptidases from the MEROPS database with corresponding IDs and Pfams.

Family	Subfamily	Name	PFAM ID
A1	A1A	pepsin A	PF00026
A1	A1A	pepsin A	PF14543
A1	A1B	nepenthesin	PF00026
A1	A1B	nepenthesin	PF14543
A1	A1B	nepenthesin	PF14541
A2	A2A	HIV-1 retropepsin	PF00077
A2	A2A	HIV-1 retropepsin	PF12382
A2	A2B	Ty3 transposon peptidase	PF12384
A2	A2C	Gypsy transposon peptidase	PF00077
A2	A2D	Osvaldo retrotransposon peptidase	PF00077
A2	A2D	Osvaldo retrotransposon peptidase	PF13650
A2	A2D	Osvaldo retrotransposon peptidase	PF13975
A3	A3A	cauliflower mosaic virus-type peptidase	PF00077
A3	A3A	cauliflower mosaic virus-type peptidase	PF02160
A3	A3A	cauliflower mosaic virus-type peptidase	PF13650
A3	A3B	bacilliform virus peptidase	PF02160
A5	null	thermopsin	PF05317
A8	null	signal peptidase II	PF01252
A9	null	spumapepsin	PF03539
A11	A11A	Copia transposon peptidase	PF01021
A11	A11A	Copia transposon peptidase	PF13976
A11	A11B	Ty1 transposon peptidase	PF01021
A22	A22A	presenilin 1	PF01080
A22	A22A	presenilin 1	PF06550
A22	A22B	impas 1 peptidase	PF04258
A22	A22B	impas 1 peptidase	PF06550
A24	A24A	type 4 prepilin peptidase 1	PF01478
A24	A24B	FlaK peptidase	PF01478
A25	null	gpr peptidase	PF03418
A26	null	omptin	PF01278
A28	A28A	DNA-damage inducible protein 1	PF00077
A28	A28A	DNA-damage inducible protein 1	PF09668
A28	A28B	skin SASPase	PF00077
A28	A28B	skin SASPase	PF09668
A28	A28B	skin SASPase	PF13650
A28	A28B	skin SASPase	PF13975
A31	null	HybD peptidase	PF01750
A32	null	PerP peptidase	PF13650
A36	null	sporulation factor SpoIIGA	PF03419
A37	null	sso1175 g.p.	null
C1	C1A	papain	PF00112
C1	C1B	bleomycin hydrolase	PF00112
C1	C1B	bleomycin hydrolase	PF03051
C2	C2A	calpain-2	PF00648
C3	C3A	poliovirus-type picornain 3C	PF00548
C3	C3B	enterovirus picornain 2A	PF00947
C3	C3C	foot-and-mouth disease virus picornain 3C	PF00548
C3	C3C	foot-and-mouth disease virus picornain 3C	PF12381
C3	C3D	cowpea mosaic comovirus-type picornain 3C	PF00548
C3	C3E	hepatitis A virus-type picornain 3C	PF00548
C3	C3F	parechovirus picornain 3C	PF00548
C3	C3G	rice tungro spherical virus-type peptidase	PF12381

C3	C3H	grapevine fanleaf-type nepovirus picornain 3C	null
C4	null	nuclear-inclusion-a peptidase	PF00863
C5	null	adenain	PF00770
C6	null	potato virus Y-type helper component peptidase	PF00851
C7	null	chestnut blight fungus virus p29 peptidase	PF01830
C8	null	chestnut blight fungus virus p48 peptidase	PF03569
C9	null	sindbis virus-type nsP2 peptidase	PF01707
C10	null	streptopain	PF01640
C11	C11A	clostripain	PF03415
C11	C11B	PNT1 peptidase	PF03415
C12	null	ubiquitinyl hydrolase-L1	PF01088
C13	null	legumain	PF01650
C14	C14A	caspase-1	PF00656
C14	C14B	metacaspase Yca1	PF00656
C15	null	pyroglutamyl-peptidase I	PF01470
C15	null	pyroglutamyl-peptidase I	PF06162
C16	C16A	murine hepatitis coronavirus papain-like peptidase 1	PF01831
C16	C16B	murine hepatitis coronavirus papain-like peptidase 2	PF08715
C18	null	hepatitis C virus peptidase 2	PF01538
C19	null	ubiquitin-specific peptidase 14	PF00443
C19	null	ubiquitin-specific peptidase 14	PF13423
C21	null	tymovirus peptidase	PF05381
C23	null	carlavirus peptidase	PF05379
C24	null	rabbit hemorrhagic disease virus 3C-like peptidase	PF03510
C25	null	gingipain RgpA	PF01364
C26	null	gamma-glutamyl hydrolase	PF00117
C26	null	gamma-glutamyl hydrolase	PF01174
C26	null	gamma-glutamyl hydrolase	PF07685
C26	null	gamma-glutamyl hydrolase	PF07722
C26	null	gamma-glutamyl hydrolase	PF09825
C27	null	rubella virus peptidase	PF05407
C28	null	foot-and-mouth disease virus L-peptidase	PF05408
C30	null	porcine transmissible gastroenteritis virus-type main peptidase	PF05409
C31	null	porcine reproductive and respiratory syndrome arterivirus-type cysteine peptidase alpha	PF05410
C32	null	equine arteritis virus-type cysteine peptidase	PF05411
C33	null	equine arteritis virus Nsp2-type cysteine peptidase	PF05412
C36	null	beet necrotic yellow vein furovirus-type papain-like peptidase	PF05415
C37	null	calicivirin	PF05416
C39	null	bacteriocin-processing peptidase	PF03412
C39	null	bacteriocin-processing peptidase	PF13529
C40	null	dipeptidyl-peptidase VI	PF00877
C40	null	dipeptidyl-peptidase VI	PF05382
C42	null	beet yellows virus-type papain-like peptidase	PF05533
C44	null	amidophosphoribosyltransferase precursor	PF00310
C44	null	amidophosphoribosyltransferase precursor	PF00733
C44	null	amidophosphoribosyltransferase precursor	PF12481
C44	null	amidophosphoribosyltransferase precursor	PF13230
C44	null	amidophosphoribosyltransferase precursor	PF13522
C44	null	amidophosphoribosyltransferase precursor	PF13537
C45	null	acyl-coenzyme A:6-aminopenicillanic acid acyl-transferase precursor	PF03417
C46	null	hedgehog protein	PF01079
C47	null	staphopain A	PF05543
C48	null	Ulp1 peptidase	PF02902
C49	null	Strawberry mottle virus peptidase	null
C50	null	separase	PF03568
C51	null	D-alanyl-glycyl peptidase	PF05257
C53	null	pestivirus Npro peptidase	PF05550
C54	null	autophagin-1	PF03416
C55	null	YopJ protein	PF03421
C56	null	PfpI peptidase	PF01965
C56	null	PfpI peptidase	PF13278
C56	null	PfpI peptidase	PF13507
C57	null	vaccinia virus I7L processing peptidase	PF03290
C58	C58A	YopT peptidase	PF03543
C58	C58B	HopN1 peptidase	PF03543
C59	null	penicillin V acylase precursor	PF02275
C60	C60A	sortase A	PF04203
C60	C60B	sortase B	PF04203
C60	C60B	sortase B	PF07170
C62	null	gill-associated virus 3C-like peptidase	PF12380
C63	null	African swine fever virus processing peptidase	PF02902
C64	null	Cezanne peptidase	PF02338
C65	null	otubain-1	PF10275
C66	null	IdeS peptidase	PF09028
C67	null	CylD peptidase	PF00443
C69	null	dipeptidase A	PF03577
C70	null	AvrRpt2 peptidase	PF12385
C71	null	pseudomurein endoisopeptidase Pei	PF12386
C74	null	pestivirus NS2 peptidase	PF12387
C75	null	AgrB peptidase	PF04647
C76	null	viral tegument protein deubiquitinylating peptidase	PF04843
C78	null	UfSP1 peptidase	PF07910
C79	null	ElaD peptidase	PF02902
C80	null	RTX self-cleaving toxin	PF11713

C82	C82A	L,D-transpeptidase	PF03734
C83	null	gamma-glutamylcysteine dipeptidyltranspeptidase	PF05023
C84	null	prtH peptidase	null
C85	C85A	OTLD1 deubiquitinylating enzyme	PF02338
C85	C85B	OTU1 peptidase	PF02338
C86	null	ataxin-3	PF02099
C87	null	nairovirus deubiquitinylating peptidase	PF02338
C89	null	acid ceramidase precursor	PF02275
C93	null	LapG peptidase	PF06035
C95	null	lysosomal 66.3 kDa protein	PF04916
C96	null	McjB peptidase	PF13471
C97	null	DeSI-1 peptidase	PF05903
C98	null	USPL1 peptidase	PF15499
C99	null	iflavirus processing peptidase	null
C100	null	agglutinin peptidase	null
C101	null	OTULIN peptidase	null
C102	null	GtgE peptidase	null
C104	null	PlyC phage lysin	null
C105	null	papain-like peptidase 1 alpha	null
C107	null	alphamesonivirus 3C-like peptidase	null
C108	null	Prp peptidase	null
C110	null	kyphoscoliosis peptidase	null
C111	null	coagulation factor XIIIa	null
C113	null	IgdE peptidase	null
C115	null	MINDY-1 protein	null
C116	null	dermonecrotic toxin	null
C117	null	SpvD g.p.	null
C118	null	EspL g.p.	null
G01	null	scytalidoglutamic peptidase	PF01828
G02	null	pre-neck appendage protein	PF11962
M1	null	aminopeptidase N	PF01433
M1	null	aminopeptidase N	PF13485
M2	null	angiotensin-converting enzyme peptidase unit 1	PF01401
M3	M3A	thimet oligopeptidase	PF01432
M3	M3B	oligopeptidase F	PF01432
M3	M3C	Pz-peptidase A	null
M4	null	thermolysin	PF01447
M4	null	thermolysin	PF02868
M5	null	mycolysin	null
M6	null	immune inhibitor A peptidase	PF05547
M7	null	snapalysin	PF02031
M8	null	leishmanolysin	PF01457
M9	M9A	bacterial collagenase V	PF01752
M9	M9B	bacterial collagenase H	PF01752
M9	M9B	bacterial collagenase H	PF08453
M10	M10A	matrix metallopeptidase-1	PF00413
M10	M10B	serralysin	PF00413
M10	M10C	fragilysin	PF00413
M11	null	gametolysin	PF05548
M12	M12A	astacin	PF01400
M12	M12B	adamalysin	PF01421
M12	M12B	adamalysin	PF13574
M12	M12B	adamalysin	PF13582
M12	M12B	adamalysin	PF13583
M12	M12B	adamalysin	PF13688
M13	null	neprilysin	PF01431
M13	null	neprilysin	PF05649
M14	M14A	carboxypeptidase A1	PF00246
M14	M14B	carboxypeptidase E	PF00246
M14	M14C	gamma-D-glutamyl-(L)-meso-diaminopimelate peptidase I	PF00246
M14	M14C	gamma-D-glutamyl-(L)-meso-diaminopimelate peptidase I	PF01476
M14	M14D	cytosolic carboxypeptidase 6	PF00246
M15	M15A	zinc D-Ala-D-Ala carboxypeptidase	PF01471
M15	M15A	zinc D-Ala-D-Ala carboxypeptidase	PF05951
M15	M15A	zinc D-Ala-D-Ala carboxypeptidase	PF08291
M15	M15B	vanY D-Ala-D-Ala carboxypeptidase	PF02557
M15	M15C	Ply118 L-Ala-D-Glu peptidase	PF01510
M15	M15C	Ply118 L-Ala-D-Glu peptidase	PF02557
M15	M15C	Ply118 L-Ala-D-Glu peptidase	PF13539
M16	M16A	pitrilysin	PF00675
M16	M16A	pitrilysin	PF05193
M16	M16B	mitochondrial processing peptidase beta-subunit	PF00675
M16	M16B	mitochondrial processing peptidase beta-subunit	PF05193
M16	M16C	eupitrilysin	PF00675
M16	M16C	eupitrilysin	PF05193
M17	null	leucine aminopeptidase 3	PF00883
M18	null	aminopeptidase I	PF02127
M19	null	membrane dipeptidase	PF01244
M20	M20A	glutamate carboxypeptidase	PF01546
M20	M20B	peptidase T	PF01546
M20	M20C	Xaa-His dipeptidase	PF01546
M20	M20D	carboxypeptidase Ss1	PF01546
M20	M20F	carnosine dipeptidase II	PF01546
M23	M23A	beta-lytic metallopeptidase	PF01551

M23	M23B	lysostaphin	PF01551
M24	M24A	methionyl aminopeptidase 1	PF00557
M24	M24B	aminopeptidase P	PF00557
M26	null	IgA1-specific metallopeptidase	PF05342
M26	null	IgA1-specific metallopeptidase	PF07580
M27	null	tentoxilysin	PF01742
M28	M28A	aminopeptidase S	PF04389
M28	M28A	aminopeptidase S	PF09940
M28	M28B	glutamate carboxypeptidase II	PF04389
M28	M28C	IAP aminopeptidase	PF04389
M28	M28D	aminopeptidase ES-62	PF04389
M28	M28E	aminopeptidase Ap1	PF04389
M28	M28F	YwaD peptidase	null
M29	null	aminopeptidase T	PF02073
M30	null	hyicolysin	PF10026
M30	null	hyicolysin	PF10460
M30	null	hyicolysin	PF13485
M32	null	carboxypeptidase Taq	PF02074
M34	null	anthrax lethal factor	PF07737
M35	null	deuterolysin	PF02102
M35	null	deuterolysin	PF14521
M36	null	fungalysin	PF02128
M38	null	isoaspartyl dipeptidase	PF00962
M38	null	isoaspartyl dipeptidase	PF01979
M38	null	isoaspartyl dipeptidase	PF04909
M38	null	isoaspartyl dipeptidase	PF07969
M38	null	isoaspartyl dipeptidase	PF12890
M38	null	isoaspartyl dipeptidase	PF13147
M38	null	isoaspartyl dipeptidase	PF13594
M41	null	FtsH peptidase	PF01434
M42	null	glutamyl aminopeptidase	PF05343
M43	M43A	cytophagalysin	PF05572
M43	M43B	pappalysin-1	PF05572
M44	null	pox virus metallopeptidase	PF03410
M48	M48A	Ste24 peptidase	PF01435
M48	M48B	HtpX peptidase	PF01435
M48	M48C	Oma1 peptidase	PF01435
M49	null	dipeptidyl-peptidase III	PF03571
M50	M50A	site 2 peptidase	PF02163
M50	M50B	sporulation factor SpoIVFB	PF02163
M50	M50B	sporulation factor SpoIVFB	PF13398
M54	null	archaelysin	PF07998
M55	null	D-aminopeptidase DppA	PF04951
M56	null	BlaR1 peptidase	PF05569
M57	null	prtB g.p.	PF12388
M60	null	enhancin	PF03272
M60	null	enhancin	PF13402
M61	null	glycyl aminopeptidase	PF05299
M61	null	glycyl aminopeptidase	PF13485
M64	null	IgA peptidase	PF09471
M66	null	StcE peptidase	PF10462
M67	M67A	RPN11 peptidase	PF01398
M67	M67A	RPN11 peptidase	PF05021
M67	M67A	RPN11 peptidase	PF14464
M67	M67B	JAMM-like protein	PF01398
M67	M67B	JAMM-like protein	PF14464
M67	M67C	STAMBP isopeptidase	PF01398
M72	null	peptidyl-Asp metallopeptidase	PF13574
M72	null	peptidyl-Asp metallopeptidase	PF13582
M72	null	peptidyl-Asp metallopeptidase	PF13583
M72	null	peptidyl-Asp metallopeptidase	PF13688
M73	null	camelysin	PF12389
M74	null	murein endopeptidase	PF03411
M75	null	imelysin	PF09375
M76	null	Atp23 peptidase	PF09768
M77	null	tryptophanyl aminopeptidase 7-DMATS-type peptidase	PF11991
M78	null	ImmA peptidase	PF06114
M79	null	RCE1 peptidase	PF02517
M80	null	Wss1 peptidase	PF08325
M81	null	microcystinase MlrC	PF07171
M81	null	microcystinase MlrC	PF07364
M82	null	PrsW peptidase	PF10086
M82	null	PrsW peptidase	PF13367
M84	null	MpriBi peptidase	PF13582
M84	null	MpriBi peptidase	PF13688
M85	null	NleC peptidase	PF13678
M86	null	PghP gamma-polyglutamate hydrolase	PF05908
M87	null	chloride channel accessory protein 1	PF08434
M88	null	IMPa peptidase	PF13402
M90	null	MtfA peptidase	PF06167
M91	null	NleD peptidase	PF14891
M93	null	BACCAC_01431 g.p.	PF15890
M95	null	selecase	PF01863
M96	null	Tiki1 peptidase	PF01963

M97	null	EcxAB peptidase	null
M98	null	YghJ g.p.	PF13402
M99	null	Csd4 peptidase	null
M100	null	spartan peptidase	null
M101	null	flagellinolysin	null
M102	null	DA1 peptidase	null
N1	null	nodavirus peptide lyase	PF01829
N2	null	tetravirus coat protein	PF03566
N4	null	Tsh-associated self-cleaving domain	PF03797
N5	null	picobirnavirus self-cleaving protein	null
N6	null	YscU protein	PF01312
N7	null	reovirus type 1 coat protein	PF05993
N8	null	poliovirus capsid VP0-type self-cleaving protein	PF00073
N8	null	poliovirus capsid VP0-type self-cleaving protein	PF02226
N9	null	intein-containing V-type proton ATPase catalytic subunit A	PF05203
N10	null	intein-containing replicative DNA helicase precursor	PF07591
N10	null	intein-containing replicative DNA helicase precursor	PF14890
N11	null	intein-containing chloroplast ATP-dependent peptide lyase	PF00574
P1	null	DmpA aminopeptidase	PF03576
P2	P2A	EGF-like module containing mucin-like hormone receptor-like 2	PF00002
P2	P2B	polycystin-1	PF01825
S1	S1A	chymotrypsin A	PF00089
S1	S1A	chymotrypsin A	PF03761
S1	S1A	chymotrypsin A	PF13365
S1	S1B	glutamyl endopeptidase I	PF13365
S1	S1C	DegP peptidase	PF00089
S1	S1C	DegP peptidase	PF13365
S1	S1D	lysyl endopeptidase	PF00089
S1	S1D	lysyl endopeptidase	PF13365
S1	S1E	streptogrisin A	PF00089
S1	S1F	astrovirus serine peptidase	PF13365
S3	null	togavirin	PF00944
S6	null	IgA1-specific serine peptidase	PF02395
S7	null	flavivirin	PF00949
S8	S8A	subtilisin Carlsberg	PF00082
S8	S8B	kexin	PF00082
S9	S9A	prolyl oligopeptidase	PF00326
S9	S9B	dipeptidyl-peptidase IV	PF00326
S9	S9B	dipeptidyl-peptidase IV	PF00756
S9	S9B	dipeptidyl-peptidase IV	PF12695
S9	S9B	dipeptidyl-peptidase IV	PF12697
S9	S9C	acylaminoacyl-peptidase	PF00135
S9	S9C	acylaminoacyl-peptidase	PF00326
S9	S9C	acylaminoacyl-peptidase	PF00756
S9	S9C	acylaminoacyl-peptidase	PF01738
S9	S9C	acylaminoacyl-peptidase	PF02230
S9	S9C	acylaminoacyl-peptidase	PF05728
S9	S9C	acylaminoacyl-peptidase	PF07859
S9	S9C	acylaminoacyl-peptidase	PF12695
S9	S9C	acylaminoacyl-peptidase	PF12697
S9	S9C	acylaminoacyl-peptidase	PF12715
S9	S9C	acylaminoacyl-peptidase	PF12740
S9	S9D	glutamyl endopeptidase C	PF00326
S9	S9D	glutamyl endopeptidase C	PF07859
S10	null	carboxypeptidase Y	PF00450
S11	null	D-Ala-D-Ala carboxypeptidase A	PF00144
S11	null	D-Ala-D-Ala carboxypeptidase A	PF00768
S11	null	D-Ala-D-Ala carboxypeptidase A	PF13354
S12	null	D-Ala-D-Ala carboxypeptidase B	PF00144
S13	null	D-Ala-D-Ala peptidase C	PF02113
S14	null	peptidase Clp	PF00574
S15	null	Xaa-Pro dipeptidyl-peptidase	PF02129
S15	null	Xaa-Pro dipeptidyl-peptidase	PF08840
S15	null	Xaa-Pro dipeptidyl-peptidase	PF12695
S15	null	Xaa-Pro dipeptidyl-peptidase	PF12697
S16	null	Lon-A peptidase	PF05362
S16	null	Lon-A peptidase	PF13541
S21	null	cytomegalovirus assemblin	PF00716
S24	null	repressor LexA	PF00717
S26	S26A	signal peptidase I	PF10502
S26	S26B	signalase	PF00461
S26	S26C	TraF peptidase	PF10502
S27	null	lysosomal Pro-Xaa carboxypeptidase	PF05577
S29	null	hepacivirin	PF02907
S30	null	potyvirus P1 peptidase	PF01577
S31	null	pestivirus NS3 polyprotein peptidase	PF05578
S32	null	equine arteritis virus serine peptidase	PF05579
S33	null	prolyl aminopeptidase	PF00561
S33	null	prolyl aminopeptidase	PF00975
S33	null	prolyl aminopeptidase	PF03096
S33	null	prolyl aminopeptidase	PF07819
S33	null	prolyl aminopeptidase	PF08386
S33	null	prolyl aminopeptidase	PF11339
S33	null	prolyl aminopeptidase	PF12146

S33	null	prolyl aminopeptidase	PF12147
S33	null	prolyl aminopeptidase	PF12695
S33	null	prolyl aminopeptidase	PF12697
S37	null	PS-10 peptidase	PF05576
S39	S39A	sobemovirus peptidase	PF02122
S39	S39B	luteovirus peptidase	PF02122
S41	S41A	C-terminal processing peptidase-1	PF03572
S41	S41B	tricorn core peptidas	PF03572
S41	S41B	tricorn core peptidas	PF14684
S45	null	penicillin G acylase precursor	PF01804
S46	null	dipeptidyl-peptidase 7	PF10459
S48	null	HetR putative peptidase	PF03574
S49	S49A	signal peptide peptidase A	PF01343
S49	S49A	signal peptide peptidase A	PF01972
S49	S49B	protein C	PF01343
S49	S49C	archaean signal peptide peptidase 1	PF01343
S49	S49C	archaean signal peptide peptidase 1	PF01972
S50	null	infectious pancreatic necrosis birnavirus Vp4 peptidase	PF01768
S51	null	dipeptidase E	PF03575
S53	null	sedolisin	PF00082
S54	null	rhomboid-1	PF01694
S54	null	rhomboid-1	PF04511
S55	null	SpoIVB peptidase	PF05580
S59	null	nucleoporin 145	PF03093
S59	null	nucleoporin 145	PF04096
S60	null	lactoferrin	PF00405
S62	null	influenza A PA peptidase	PF00603
S64	null	Ssy5 peptidase	PF08192
S65	null	picornain-like serine peptidase	null
S66	null	murein tetrapeptidase LD-carboxypeptidase	PF02016
S68	null	PIDD auto-processing protein unit 1	PF10461
S69	null	Tellina virus 1 VP4 peptidase	PF01766
S71	null	MUC1 self-cleaving mucin	PF01390
S72	null	dystroglycan	PF05454
S73	null	gpO peptidase	PF05929
S74	null	Escherichia coli phage K1F endosialidase CIMCD self-cleaving protein	PF13884
S75	null	White bream virus serine peptidase	null
S77	null	prohead peptidase gp21	PF03420
S78	null	prohead peptidase	PF04586
S79	null	CARD8 self-cleaving protein	PF13553
S80	null	prohead peptidase gp175	null
S81	null	destabilase	PF05497
S82	null	autocrine proliferation repressor protein A	null
T1	T1A	archaean proteasome, beta component	PF00227
T1	T1A	archaean proteasome, beta component	PF10584
T1	T1B	HslV component of HslUV peptidase	PF00227
T2	null	glycosylasparaginase precursor	PF01112
T3	null	gamma-glutamyltransferase 1	PF01019
T5	null	ornithine acetyltransferase precursor	PF01960
T7	null	CwpV	null
T8	null	HopB1 g.p.	null
U32	null	collagenase	PF01136
U40	null	protein P5 murein endopeptidase	PF10464
U49	null	Lit peptidase	PF10463
U56	null	homomultimeric peptidase	PF04454
U57	null	yabG protein	PF05582
U62	null	microcin-processing peptidase 1	PF01523
U69	null	AIDA-I self-cleaving autotransporter protein	PF03212
U72	null	Dop isopeptidase	PF03136
U73	null	small protease	PF04264
U74	null	neprosin	PF03080
U75	null	Ras/Rap1-specific peptidase	null

Supplementary Table 5. Corresponding metadata (environmental parameters) for *Tara* Oceans samples. Latitude (degrees North); Size (Tara size fraction scaled from 0-8); Depth (m); Temp (Temperature °C); Oxygen (μmol kg^{-1}); Chloro (HPLC chlorophyll mg Chl m^{-3}); PO4 (phosphate μM); NO2NO3 (nitrite + nitrate μM); Sal (salinity PSU)

Abbrev. ID	Metagenomic Library Accessions	Latitude	Size	Depth	Temp	Oxygen	Chloro	PO4	N02NO3	Sal
tara004_bact_dcm	ERR598950, ERR599095	NaN	5	NaN	NaN	NaN	NaN	NaN	NaN	NaN
tara004_bact_surface	ERR598955, ERR599003	NaN	5	NaN	NaN	NaN	NaN	NaN	NaN	NaN
tara007_girus_dcm	ERR315856	37.0211	5	40	17.8403	NaN	0.2959	0.2535	9.9505	37.0928
tara007_girus_surface	ERR315857	37.0211	5	5	23.8457	NaN	0.075	0.218	4.1205	37.4815
tara007_prot_dcm	ERR538182, ERR550537, ERR562726	37.0211	8	40	17.8403	NaN	0.2959	0.2535	9.9505	37.0928
tara007_prot_surface	ERR550396, ERR550403, ERR562530, ERR654518	37.0211	8	5	23.8457	NaN	0.075	0.218	4.1205	37.4815
tara009_girus_dcm	ERR594315, ERR594329	39.1633	5	55	15.8022	NaN	0.5447	NaN	NaN	37.5287

tara009_girus_surface	ERR594288, ERR594316, ERR594317	39.1633	5	5	24.5247	NaN	0	NaN	NaN	37.8064
tara009_prot_dcm	ERR868473	39.1633	8	55	15.8022	NaN	0.5447	NaN	NaN	37.5287
tara009_prot_surface	ERR868407	39.1633	8	5	24.5247	NaN	0	NaN	NaN	37.8064
tara018_bact_dcm	ERR599073, ERR599092	35.7606	5	48.723	21.3101	209.593	0.147566	0.0201	0.038	37.9295
tara018_bact_surface	ERR598993, ERR599140	35.7606	5	3.978	21.5526	208.084	0.066985	0.0256	0.118	37.8999
tara023_bact_dcm	ERR315859, ERR315860	42.1744	5	48.696	15.95485	227.0715	0.29645	0.012	0.05	38.30335
tara023_bact_surface	ERR315858, ERR315861	42.1744	5	4.402	17.0572	223.516	0.1299	0.0105	0.04825	38.2175
tara023_prot_dcm	ERR538175	42.1744	8	48.696	15.95485	227.0715	0.29645	0.012	0.05	38.30335
tara023_prot_surface	ERR538173	42.1744	8	4.402	17.0572	223.516	0.1299	0.0105	0.04825	38.2175
tara025_bact_dcm	ERR599094, ERR599153	39.4085	5	48.622	15.1954	238.087	0.227128	0.073	0.154	38.4808
tara025_bact_surface	ERR598951, ERR599043	39.4085	5	3.992	18.3544	218.089	0.147566	0.0065	0.0255	38.1886
tara030_bact_dcm	ERR318618, ERR318619, ERR318620, ERR318621	33.9235	5	68.783	18.41	222.997	0.165775	NaN	NaN	39.1998
tara030_bact_surface	ERR315862, ERR315863	33.9202	5	4.236	20.4437	207.533	0.11119	0	0.0455	39.4227
tara030_prot_dcm	ERR538179	33.9235	8	68.783	18.41	222.997	0.165775	NaN	NaN	39.1998
tara030_prot_surface	ERR538186	33.9202	8	4.236	20.4437	207.533	0.11119	0	0.0455	39.4227
tara031_bact_surface	ERR598969, ERR599106	27.145	5	3.95	24.993	191.23	0	0.0235	0.03	39.919
tara032_bact_dcm	ERR599061, ERR599097	23.3867	5	78.4975	26.1435	182.6315	0.256459	0.025	0.012	40.18055
tara032_bact_surface	ERR599041, ERR599116, ERR599155	23.3867	5	4.095	25.9619	187.332	0	0.0175	0	39.716
tara033_bact_surface	ERR599049, ERR599134	22.1233	5	3.976	27.2923	184.416	0.068955	0.0525	0.0045	38.9505
tara034_bact_dcm	ERR598975, ERR599111	18.3983	5	58.607	27.579	181.945	0.212492	0.0885	0.635	38.8893
tara034_bact_surface	ERR598959, ERR598991	18.3983	5	4.201	27.6643	184.3465	0.242561	0.185	0.029	38.6332
tara034_girus_surface	ERR594328	18.3983	2	4.201	27.6643	184.3465	0.242561	0.185	0.029	38.6332
tara036_bact_dcm	ERR598974, ERR599028	20.8176	5	19.053	25.23695	211.768	0.61899	0.514	2.078	36.5421
tara036_bact_surface	ERR598966, ERR599143	20.8176	5	3.926	26.0199	211.691	0.165775	0.367	0.131	36.5269
tara036_girus_surface	ERR594334	20.8176	2	3.926	26.0199	211.691	0.165775	0.367	0.131	36.5269
tara037_girus_meso	ERR594290, ERR594345	20.8415	2	594.608	11.9499	0	0	NaN	NaN	35.683
tara037_prot_meso	ERR873962, ERR873969	20.8415	8	594.608	11.9499	0	0	NaN	NaN	35.683
tara038_girus_meso	ERR594312	19.0208	2	336.82	14.8724	2.447	0	NaN	NaN	36.0077
tara038_girus_surface	ERR594330	19.0386	2	3.9455	26.27865	198.1505	0.181885	NaN	NaN	36.621
tara038_prot_dcm	ERR868414	19.0258	8	23.904	25.809	203.074	0.584227	0.417	1.858	NaN
tara038_prot_meso	ERR868353	19.0208	8	336.82	14.8724	2.447	0	NaN	NaN	36.0077
tara038_prot_surface	ERR868498, ERR868503	19.0386	8	3.9455	26.27865	198.1505	0.181885	NaN	NaN	36.621
tara039_bact_dcm	ERR599145	18.5939	5	23.925	26.8534	191.926	0.528296	NaN	NaN	36.4707
tara039_bact_meso	ERR599037, ERR599172	18.7341	5	266.885	15.5942	2.3085	0.040949	NaN	NaN	35.9102
tara039_girus_meso	ERR594346	18.7341	2	266.885	15.5942	2.3085	0.040949	NaN	NaN	35.9102
tara039_girus_surface	ERR594327	18.5939	2	3.751	27.0367	191.855	0.1299	NaN	NaN	36.3512
tara041_girus_surface	ERR594295	14.56	2	3.883	29.6726	185.618	0	0.144	0.088	36.0636
tara041_virus_dcm	ERR594366, ERR594367, ERR594371, ERR594372, ERR594373	14.5822	1	58.642	27.5759	191.079	0.372521	NaN	NaN	36.5184
tara041_virus_surface	ERR594384	14.56	1	3.883	29.6726	185.618	0	0.144	0.088	36.0636
tara042_bact_dcm	ERR599013, ERR599130	5.9997	5	78.561	27.9444	159.585	0.407267	0.342	1.385	35.035
tara042_bact_surface	ERR599075, ERR599141	5.9997	5	3.928	30.3376	187.9115	0	0.084	0.026	34.5739
tara042_virus_dcm	ERR594413	5.9997	1	78.561	27.9444	159.585	0.407267	0.342	1.385	35.035
tara042_virus_surface	ERR594398, ERR594403	5.9997	1	3.928	30.3376	187.9115	0	0.084	0.026	34.5739
tara045_bact_surface	ERR599045, ERR599054	1.0689	5	2.158	31.2344	186.327	0	NaN	NaN	34.9707
tara046_girus_surface	ERR599356	-0.66245	1	3.951	30.1218	185.283	0.165775	NaN	NaN	35.111
tara046_virus_surface	ERR594376	-0.66245	1	3.951	30.1218	185.283	0.165775	NaN	NaN	35.111
tara056_bact_meso	ERR599112	-15.3379	6	990.512	6.2402	87.902	0	NaN	NaN	34.8332
tara056_bact_surface	ERR599057	NaN	6	NaN	NaN	NaN	NaN	NaN	NaN	NaN
tara056_virus_meso	ERR599371	-15.3379	1	990.512	6.2402	87.902	0	NaN	NaN	34.8332
tara056_virus_surface	ERR599376	NaN	1	NaN	NaN	NaN	NaN	NaN	NaN	NaN
tara057_bact_surface	ERR599058	-16.0917	6	3.8485	27.2767	192.2445	0.11119	-0.622313	-0.116682	35.0585
tara058_bact_dcm	ERR599026	-17.3189	6	58.737	25.8182	197.3175	0.384245	-0.594677	0.063072	35.167
tara058_virus_dcm	ERR599362	-17.3189	1	58.737	25.8182	197.3175	0.384245	-0.594677	0.063072	35.167
tara062_bact_surface	ERR599012	NaN	6	NaN	NaN	NaN	NaN	NaN	NaN	NaN
tara062_virus_surface	ERR599339	NaN	1	NaN	NaN	NaN	NaN	NaN	NaN	NaN
tara064_bact_dcm	ERR598972, ERR599023, ERR599025	-29.4956	6	59.401	22.2177	207.7045	0.212492	0.0815	0.015	35.3244
tara064_bact_meso	ERR599021, ERR599164	-29.5046	6	990.711	7.6584	192.352	0	NaN	NaN	34.5801
tara064_bact_surface	ERR598970, ERR599088, ERR599150	-29.4956	6	4.752	22.223	209.658	0.11119	0.084	0	35.3214
tara064_girus_dcm	ERR594324	-29.4956	2	59.401	22.2177	207.7045	0.212492	0.0815	0.015	35.3244
tara064_prot_meso	ERR599225	-29.5046	8	990.711	7.6584	192.352	0	NaN	NaN	34.5801
tara064_virus_dcm	ERR594385	-29.4956	1	59.401	22.2177	207.7045	0.212492	0.0815	0.015	35.3244
tara064_virus_meso	ERR599351	-29.5046	1	990.711	7.6584	192.352	0	NaN	NaN	34.5801
tara064_virus_surface	ERR594392	-29.4956	1	4.752	22.223	209.658	0.11119	0.084	0	35.3214
tara065_bact_dcm	ERR598990, ERR599018, ERR599110	-35.2515	6	28.711	21.8139	206.255	0.283378	NaN	NaN	NaN
tara065_bact_meso	ERR598960, ERR599034	-35.2789	6	792.785	9.3892	202.157	0	NaN	NaN	34.7414
tara065_bact_surface	ERR598979, ERR599146	NaN	6	NaN	NaN	NaN	NaN	NaN	NaN	NaN
tara065_girus_dcm	ERR594291	-35.2515	2	28.711	21.8139	206.255	0.283378	NaN	NaN	NaN
tara065_girus_surface	ERR594320	NaN	2	NaN	NaN	NaN	NaN	NaN	NaN	NaN
tara065_virus_dcm	ERR594382, ERR594414	-35.2515	1	28.711	21.8139	206.255	0.283378	NaN	NaN	NaN
tara066_bact_dcm	ERR598982, ERR599107	-34.8901	6	28.42	15.0296	240.744	0.430696	NaN	NaN	35.3315
tara066_bact_surface	ERR598973, ERR599068, ERR599173	-34.9363	6	3.908	15.0573	239.2995	0.283378	0.343	3.34	35.334
tara066_virus_dcm	ERR594389	-34.8901	1	28.42	15.0296	240.744	0.430696	NaN	NaN	35.3315
tara066_virus_surface	ERR594362	-34.9363	1	3.908	15.0573	239.2995	0.283378	0.343	3.34	35.334
tara067_girus_surface	ERR598994, ERR599144	-32.215	6	3.8305	14.9346	250.294	0.712852	NaN	NaN	35.3054
tara067_girus_surface	ERR594313, ERR594325	-32.215	4	3.8305	14.9346	250.294	0.712852	NaN	NaN	35.3054

tara067_virus_surface	ERR594395, ERR594404	-32.215	1	3.8305	14.9346	250.294	0.712852	NaN	NaN	35.3054
tara068_bact_dcm	ERR599017, ERR599056, ERR599103	-31.0616	6	38.77	16.7478	233.899	0.666209	0.227	1.08	35.6837
tara068_bact_meso	ERR598947, ERR599131	-31.0528	6	693.73	7.47825	193.3835	0	NaN	NaN	34.5142
tara068_bact_surface	ERR599129, ERR599171, ERR599174	-31.0616	6	2.046	17.0055	233.313	0.527444	0.229	1.303	35.6904
tara068_girus_dcm	ERR594294, ERR594348	-31.0616	4	38.77	16.7478	233.899	0.666209	0.227	1.08	35.6837
tara068_girus_meso	ERR594302	-31.0528	7	693.73	7.47825	193.3835	0	NaN	NaN	34.5142
tara068_girus_surface	ERR594318, ERR594297	-31.0616	4	2.046	17.0055	233.313	0.527444	0.229	1.303	35.6904
tara068_prot_dcm	ERR599284	-31.0616	8	38.77	16.7478	233.899	0.666209	0.227	1.08	35.6837
tara068_virus_meso	ERR599382	-31.0528	1	693.73	7.47825	193.3835	0	NaN	NaN	34.5142
tara068_virus_surface	ERR594391	-31.0616	1	2.046	17.0055	233.313	0.527444	0.229	1.303	35.6904
tara070_bact_meso	ERR599044, ERR599149	-20.3862	6	792.539	4.1806	163.512	0	NaN	NaN	34.4415
tara070_bact_surface	ERR599135, ERR599165	-20.4112	6	4.04	19.8679	215.2365	0.156078	0.364	0.986	36.3746
tara070_girus_meso	ERR594299, ERR594308, ERR594331	-20.3862	4	792.539	4.1806	163.512	0	NaN	NaN	34.4415
tara070_girus_surface	ERR594349, ERR594335	-20.4112	4	4.04	19.8679	215.2365	0.156078	0.364	0.986	36.3746
tara070_virus_meso	ERR594407	-20.3862	1	792.539	4.1806	163.512	0	NaN	NaN	34.4415
tara070_virus_surface	ERR594353	-20.4112	1	4.04	19.8679	215.2365	0.156078	0.364	0.986	36.3746
tara072_bact_dcm	ERR599133, ERR599137	-8.7026	6	98.3045	23.8798	199.4	0.41858	0.143	0.044	36.6898
tara072_bact_meso	ERR599005, ERR599048	-8.7953	6	792.766	4.7021	141.71	0	NaN	NaN	34.4703
tara072_bact_surface	ERR598984, ERR599105	-8.7026	6	4.196	25.1569	199.6565	0	0.104	0.018	36.4254
tara072_virus_dcm	ERR594379	-8.7026	1	98.3045	23.8798	199.4	0.41858	0.143	0.044	36.6898
tara072_virus_meso	ERR594388	-8.7953	1	792.766	4.7021	141.71	0	NaN	NaN	34.4703
tara072_virus_surface	ERR594364	-8.7026	1	4.196	25.1569	199.6565	0	0.104	0.018	36.4254
tara076_bact_dcm	ERR599040, ERR599148	-21.0621	6	148.1035	21.9409	203.8055	0.147566	NaN	NaN	36.7941
tara076_bact_meso	ERR599154	-20.9742	6	792.737	4.7802	190.3565	0	NaN	NaN	34.3704
tara076_girus_dcm	ERR594321, ERR594298	-21.0621	4	148.1035	21.9409	203.8055	0.147566	NaN	NaN	36.7941
tara076_girus_meso	ERR594333	-20.9742	7	792.737	4.7802	190.3565	0	NaN	NaN	34.3704
tara076_girus_surface	ERR594310, ERR594286	-20.999	4	4.1135	23.2997	205.688	0	0.055883	-0.422054	37.0669
tara076_virus_dcm	ERR594355	-21.0621	1	148.1035	21.9409	203.8055	0.147566	NaN	NaN	36.7941
tara076_virus_meso	ERR599347, ERR599370	-20.9742	1	792.737	4.7802	190.3565	0	NaN	NaN	34.3704
tara076_virus_surface	ERR594354	-20.999	1	4.1135	23.2997	205.688	0	0.055883	-0.422054	37.0669
tara078_bact_dcm	ERR599046, ERR599101	-30.1487	6	118.228	19.2805	216.167	0.212492	NaN	NaN	36.2744
tara078_bact_meso	ERR599124, ERR599159	-30.1468	6	792.738	5.8659	214.842	0	NaN	NaN	34.3629
tara078_girus_dcm	ERR594336, ERR594303	-30.1487	4	118.228	19.2805	216.167	0.212492	NaN	NaN	36.2744
tara078_girus_meso	ERR594289	-30.1468	7	792.738	5.8659	214.842	0	NaN	NaN	34.3629
tara078_girus_surface	ERR594340, ERR594332	-30.1551	4	3.599	20.3618	221.706	0.040949	-0.079	0.017	36.3248
tara078_virus_dcm	ERR599374	-30.1487	1	118.228	19.2805	216.167	0.212492	NaN	NaN	36.2744
tara078_virus_surface	ERR594411	-30.1551	1	3.599	20.3618	221.706	0.040949	-0.079	0.017	36.3248
tara082_virus_dcm	ERR594409	-47.2129	1	28.841	7.0344	308.32	1.019725	1.296	17.98	34.0537
tara082_virus_surface	ERR599384	-47.1847	1	3.695	7.3324	254.672	0.256459	NaN	NaN	34.0475
tara093_bact_dcm	ERR598965	-33.8469	6	33.753	16.6594	240.436	1.144411	NaN	NaN	34.2584
tara093_bact_surface	ERR599064	-34.0665	6	3.901	18.2387	241.349	0.197431	0.515	0.017	34.3289
tara093_prot_dcm	ERR868358	-33.8469	8	33.753	16.6594	240.436	1.144411	NaN	NaN	34.2584
tara093_prot_surface	ERR868416	-34.0665	8	3.901	18.2387	241.349	0.197431	0.515	0.017	34.3289
tara094_bact_surface	ERR599050	-33.2471	6	3.927	19.4648	222.9405	0.1299	NaN	NaN	34.5
tara096_bact_surface	ERR598967	-30.4157	6	3.9515	23.1067	206.824	0.040949	NaN	NaN	35.5244
tara096_prot_dcm	ERR868415	-29.668	8	152.86	18.1104	220.355	0.256459	NaN	NaN	35.3273
tara096_prot_surface	ERR868404	-30.4157	8	3.9515	23.1067	206.824	0.040949	NaN	NaN	35.5244
tara098_bact_dcm	ERR599042, ERR599079	-25.8259	6	187.976	20.0319	210.332	0.148998	NaN	NaN	35.825
tara098_bact_meso	ERR599071, ERR599085	-25.8087	6	480.567	7.9158	154.79	0	2.023	27.2885	34.3596
tara098_bact_surface	ERR599093, ERR599120	-25.8252	6	3.7005	25.1282	200.1155	0	0.2	0.0465	36.3787
tara098_prot_dcm	ERR1712093, ERR1712225	-25.8259	8	187.976	20.0319	210.332	0.148998	NaN	NaN	35.825
tara099_bact_surface	ERR599024	-21.1724	6	3.8375	24.01875	203.5655	0	0.2895	0.0355	36.147
tara100_prot_dcm	ERR868424, ERR868465	-12.9945	8	49.125	24.6961	201.176	0.372521	0.7785	5.6545	35.77555
tara100_prot_meso	ERR868453, ERR868457	-12.9391	8	174.9465	13.0484	0.6255	0.181885	NaN	NaN	34.854
tara100_prot_surface	ERR868493	-12.9945	8	3.934	25.2711	198.83	0.270056	0.677	6.156	35.8349
tara100_virus_dcm	ERR599359	-12.9945	1	49.125	24.6961	201.176	0.372521	0.7785	5.6545	35.77555
tara100_virus_meso	ERR599375	-12.9391	1	174.9465	13.0484	0.6255	0.181885	NaN	NaN	34.854
tara100_virus_surface	ERR599342	-12.9945	1	3.934	25.2711	198.83	0.270056	0.677	6.156	35.8349
tara102_prot_dcm	ERR868422, ERR868440	-5.2697	8	39.1065	19.36175	125.113	0.803339	1.8615	24.3645	34.85785
tara102_prot_meso	ERR868362	-5.2749	8	475.988	9.1079	6.636	0	NaN	NaN	34.688
tara102_prot_surface	ERR868357	-5.2697	8	3.74	25.1061	205.1105	0.22833	1.0035	12.573	34.7037
tara102_virus_dcm	ERR599349	-5.2697	1	39.1065	19.36175	125.113	0.803339	1.8615	24.3645	34.85785
tara102_virus_meso	ERR599346	-5.2749	1	475.988	9.1079	6.636	0	NaN	NaN	34.688
tara102_virus_surface	ERR599353	-5.2697	1	3.74	25.1061	205.1105	0.22833	1.0035	12.573	34.7037
tara109_prot_dcm	ERR868450, ERR868472	2.0422	8	29.005	26.3384	202.663	0.589128	0.50475	4.638	34.4176
tara109_prot_meso	ERR868470, ERR868506	2.0841	8	376.307	11.163	1.845	0	NaN	NaN	34.7899
tara109_prot_surface	ERR868374, ERR868441	2.0422	8	3.744	27.9235	198.856	0.212492	NaN	NaN	33.1688
tara109_virus_dcm	ERR594357, ERR594380, ERR594381, ERR594383, ERR594387	2.0422	1	29.005	26.3384	202.663	0.589128	0.50475	4.638	34.4176
tara109_virus_surface	ERR594412	2.0422	1	3.744	27.9235	198.856	0.212492	NaN	NaN	33.1688
tara110_bact_dcm	ERR599014	-1.9245	6	48.604	22.2632	162.385	0.558634	0.9295	10.701	34.9474
tara110_bact_meso	ERR599020	-1.8902	6	376.597	10.3738	0.624	0	NaN	NaN	34.7643
tara110_bact_surface	ERR599039	-1.9245	6	3.977	24.058	193.386	0.297528	0.751	8.243	35.0184
tara110_prot_dcm	ERR868364, ERR868438	-1.9245	8	48.604	22.2632	162.385	0.558634	0.9295	10.701	34.9474
tara110_prot_meso	ERR868443	-1.8902	8	376.597	10.3738	0.624	0	NaN	NaN	34.7643
tara110_prot_surface	ERR868442	-1.9245	8	3.977	24.058	193.386	0.297528	0.751	8.243	35.0184
tara111_bact_meso	ERR599086	-16.9486	6	346.584	10.8438	3.134	0	NaN	NaN	34.70905
tara111_prot_dcm	ERR868381	-16.9559	8	88.4425	19.9874	213.302	0.378401	0.4375	0.931	35.69855
tara111_prot_meso	ERR873963, ERR599368	-16.9486	8	346.584	10.8438	3.134	0	NaN	NaN	34.70905
tara111_prot_surface	ERR868476	-16.9604	8	3.693	22.7599	207.736	0.165775	NaN	NaN	35.9722

tara111_virus_dcm	ERR599369	-16.9559	1	88.4425	19.9874	213.302	0.378401	0.4375	0.931	35.69855
tara111_virus_surface	ERR599357	-16.9604	1	3.693	22.7599	207.736	0.165775	NaN	NaN	35.9722
tara112_bact_dcm	ERR598957	-23.23	6	153.181	21.6683	207.9275	0.212492	0.109	0.0205	35.85885
tara112_bact_meso	ERR599072	-23.2232	6	689.7385	5.7082	202.0875	0	NaN	NaN	34.3026
tara112_bact_surface	ERR598954	-23.23	6	8.863	24.3149	200.486	0.040949	0.142	0.021	36.4532
tara122_girus_dcm	ERR594284, ERR594304, ERR594301	-8.9966	3	113.299	25.3013	178.4745	0.347595	0.58545	2.7985	35.87635
tara122_girus_meso	ERR594309, ERR594305, ERR594322	-8.9856	3	594.497	7.1574	33.827	0	NaN	NaN	34.5784
tara122_girus_surface	ERR594292, ERR594307, ERR594306	-8.9966	3	3.6865	26.5817	186.7565	0.11119	0.56795	5.456	35.3648
tara122_prot_dcm	ERR868419, ERR868425	-8.9966	8	113.299	25.3013	178.4745	0.347595	0.58545	2.7985	35.87635
tara122_prot_meso	ERR873959, ERR873968	-8.9856	8	594.497	7.1574	33.827	0	NaN	NaN	34.5784
tara122_prot_surface	ERR868475, ERR868513	-8.9966	8	3.6865	26.5817	186.7565	0.11119	0.56795	5.456	35.3648
tara122_virus_dcm	ERR599377	-8.9966	1	113.299	25.3013	178.4745	0.347595	0.58545	2.7985	35.87635
tara122_virus_meso	ERR599341	-8.9856	1	594.497	7.1574	33.827	0	NaN	NaN	34.5784
tara122_virus_surface	ERR599380	-8.9966	1	3.6865	26.5817	186.7565	0.11119	0.56795	5.456	35.3648
tara123_girus_epi	ERR594293, ERR594337, ERR594319	-8.9075	3	148.136	22.3191	165.726	0.040949	0.65945	5.4315	35.94535
tara123_girus_surface	ERR594326, ERR594347	-8.9075	4	3.8675	26.62	189.2825	0.165775	0.53495	4.9015	35.3547
tara123_prot_epi	ERR868360, ERR868481	-8.9075	8	148.136	22.3191	165.726	0.040949	0.65945	5.4315	35.94535
tara123_prot_surface	ERR868466, ERR868469	-8.9075	8	3.8675	26.62	189.2825	0.165775	0.53495	4.9015	35.3547
tara123_virus_epi	ERR599343, ERR599348	-8.9075	1	148.136	22.3191	165.726	0.040949	0.65945	5.4315	35.94535
tara123_virus_surface	ERR599378, ERR634968	-8.9075	1	3.8675	26.62	189.2825	0.165775	0.53495	4.9015	35.3547
tara124_girus_epi	ERR594285, ERR594343, ERR594338	-9.1501	3	118.175	24.48495	178.1925	0.148998	0.49125	1.1845	36.1555
tara124_girus_surface	ERR594287, ERR594311, ERR594296	-8.8521	3	3.912	26.5758	189.723	0.46445	NaN	NaN	35.3693
tara124_prot_epi	ERR868371, ERR868491, ERR868494	-9.1501	8	118.175	24.48495	178.1925	0.148998	0.49125	1.1845	36.1555
tara124_prot_surface	ERR588857, ERR868363, ERR868489	-8.8521	8	3.912	26.5758	189.723	0.46445	NaN	NaN	35.3693
tara124_virus_epi	ERR599367	-9.1501	1	118.175	24.48495	178.1925	0.148998	0.49125	1.1845	36.1555
tara124_virus_surface	ERR599354	-8.8521	1	3.912	26.5758	189.723	0.46445	NaN	NaN	35.3693
tara125_girus_epi	ERR594341, ERR594342, ERR594300	-8.8999	3	138.242	24.3076	159.652	0.040949	NaN	NaN	36.3271
tara125_girus_surface	ERR594344, ERR594339, ERR594323	-8.8818	3	3.782	26.8707	187.438	0.1299	0.55725	3.7335	35.4211
tara125_prot_epi	ERR868359, ERR868426	-8.8999	8	138.242	24.3076	159.652	0.040949	NaN	NaN	36.3271
tara125_prot_surface	ERR868352, ERR868382	-8.8818	8	3.782	26.8707	187.438	0.1299	0.55725	3.7335	35.4211
tara125_virus_epi	ERR599338	-8.8999	1	138.242	24.3076	159.652	0.040949	NaN	NaN	36.3271
tara125_virus_surface	ERR599337	-8.8818	1	3.782	26.8707	187.438	0.1299	0.55725	3.7335	35.4211
tara128_bact_dcm	ERR599032	0.0005	6	38.88	26.0828	177.115	0.475182	0.54925	5.264	35.1296
tara128_bact_surface	ERR599038	0.0005	6	3.69	26.2312	179.341	0.148998	0.53975	5.0815	35.1166
tara128_prot_dcm	ERR868458	0.0005	8	38.88	26.0828	177.115	0.475182	0.54925	5.264	35.1296
tara128_prot_surface	ERR868462	0.0005	8	3.69	26.2312	179.341	0.148998	0.53975	5.0815	35.1166
tara133_bact_dcm	ERR598942	35.4068	6	43.666	14.7316	261.053	0.971687	0.46075	1.302	33.1293
tara133_bact_meso	ERR599115	35.2859	6	644.11	4.9214	8.534	0	NaN	NaN	34.3283
tara133_bact_surface	ERR599052	35.4068	6	4.078	19.32465	221.9735	0.055682	0.29175	0.0165	33.0979
tara137_bact_dcm	ERR598987, ERR599070, ERR599099, ERR599147	14.1749	6	38.653	22.4127	164.005	0.685481	0.884	6.0475	34.3302
tara137_bact_surface	ERR598989	14.1749	6	3.908	26.5526	194.8275	0.165775	0.456	2.444	33.8657
tara137_prot_dcm	ERR868492	14.1749	8	38.653	22.4127	164.005	0.685481	0.884	6.0475	34.3302
tara137_prot_meso	ERR868512	14.2025	8	371.768	8.9578	0.683	0	NaN	NaN	34.5897
tara137_prot_surface	ERR868477	14.1749	8	3.908	26.5526	194.8275	0.165775	0.456	2.444	33.8657
tara137_virus_dcm	ERR599340	14.1749	1	38.653	22.4127	164.005	0.685481	0.884	6.0475	34.3302
tara137_virus_meso	ERR599366	14.2025	1	371.768	8.9578	0.683	0	NaN	NaN	34.5897
tara137_virus_surface	ERR599363	14.1749	1	3.908	26.5526	194.8275	0.165775	0.456	2.444	33.8657
tara138_prot_dcm	ERR868398	6.3094	8	58.419	21.5415	123.126	0.430696	1.108	12.904	34.5573
tara138_prot_meso	ERR868467	6.3572	8	445.8565	8.23995	0.9255	0	NaN	NaN	34.6103
tara138_prot_surface	ERR868390	6.3332	8	3.745	26.5769	195.645	0	NaN	NaN	33.2033
tara138_virus_dcm	ERR599345	6.3094	1	58.419	21.5415	123.126	0.430696	1.108	12.904	34.5573
tara138_virus_meso	ERR599365	6.3572	1	445.8565	8.23995	0.9255	0	NaN	NaN	34.6103
tara138_virus_surface	ERR599355	6.3332	1	3.745	26.5769	195.645	0	NaN	NaN	33.2033
tara140_bact_surface	ERR599162	7.4194	6	4.015	26.6736	205.1455	0.068955	0.0812	-0.0005	28.9144
tara141_bact_surface	ERR599029	9.8374	6	2.2575	27.3376	194.7225	0.066985	0.0142	0	34.6516
tara142_bact_dcm	ERR599100	25.5101	6	123.5775	25.0027	192.9375	0.197431	0.0215	0.0615	36.1977
tara142_bact_meso	ERR598985	25.6665	6	634.2345	9.7093	107.5585	0	NaN	NaN	35.1637
tara142_bact_surface	ERR599136	25.5101	6	4.063	24.962	192.63	0.197431	0.003	0.049	36.1913
tara145_bact_meso	ERR599166	39.2392	6	584.707	4.8635	236.592	0.040949	NaN	NaN	34.9656
tara145_bact_surface	ERR598983	39.2003	6	4.026	14.0646	235.135	0.322958	0.3535	4.3775	35.1371
tara146_bact_meso	ERR599047	34.6663	6	634.2595	10.33405	135.904	0	NaN	NaN	35.3152
tara146_bact_surface	ERR598968	34.841	6	4.0595	19.46795	214.0965	0.165775	0.022	0.7375	36.5426
tara146_prot_meso	ERR868449	34.6663	8	634.2595	10.33405	135.904	0	NaN	NaN	35.3152
tara146_prot_surface	ERR1719409	34.841	8	4.0595	19.46795	214.0965	0.165775	0.022	0.7375	36.5426
tara148_prot_surface	ERR868395	31.8321	8	4.1535	20.487	214.237	0.11119	0.0015	0.1505	36.5979
tara149_prot_meso	ERR868380	34.0771	8	733.559	10.4136	153.3315	0	NaN	NaN	35.3574
tara149_prot_surface	ERR868392	34.0964	8	3.6375	18.7425	219.196	0.165775	NaN	NaN	36.4095
tara150_bact_dcm	ERR598996	35.8579	6	38.891	17.5217	228.791	0.384245	0	0.1745	36.3026
tara150_prot_dcm	ERR868511	35.8579	8	38.891	17.5217	228.791	0.384245	0	0.1745	36.3026
tara150_prot_surface	ERR1711990, ERR1726817, ERR1719258	35.8579	8	3.851	17.595	229.344	0.147566	0.0055	0.1695	36.3036
tara151_bact_dcm	ERR598986	36.1811	6	78.6725	16.8847	230.5355	0.29645	NaN	NaN	36.2075
tara151_prot_dcm	ERR868423	36.1811	8	78.6725	16.8847	230.5355	0.29645	NaN	NaN	36.2075

tara152_bact_meso	ERR598944	43.734	6	792.735	10.1737	174.719	0	NaN	NaN	35.5657
tara152_bact_mixed	ERR599001	43.6876	6	157.988	13.8959	235.338	0.040949	0.2605	4.196	35.9021
tara152_bact_surface	ERR599078	43.6876	6	3.64	14.2865	242.997	0.283378	0.1595	2.159	35.9867
tara152_prot_dcm	ERR1719178, ERR1719417, ERR1726761, ERR1719398, ERR1726972, ERR1711934, ERR1712010	43.6876	8	23.974	14.2727	242.2005	0.372521	0.167	2.2565	35.9849
tara152_prot_meso	ERR868434	43.734	8	792.735	10.1737	174.719	0	NaN	NaN	35.5657

Supplementary Table 6. Corresponding metadata (environmental parameters) for Ocean Sampling Day samples. Lat (latitude degree North); Depth (m); Temp (temperature °C); PO4 (phosphate µM); NO3 (nitrate µM); Oxygen (µmol kg^{-1}); Coast (distance from coast in m).

label	Run ID	Lat	Depth	Site Name	Province	Temp	PO4	NO3	Oxygen	Coast
OSD100	ERR771104	35.35	1	Crete - GOS	Aegean Sea	24.21	0.01	0.05	NaN	1498
OSD101	ERR771103	32.741808	0	Quinta do Lorde	North Atlantic Ocean	20.5	NaN	NaN	NaN	0
OSD102	ERR771102	32.64605	0	Marina do Funchal	North Atlantic Ocean	20.8	NaN	NaN	NaN	0
OSD103	ERR771101	32.7747	0	Porto da Cruz	North Atlantic Ocean	20.2	NaN	NaN	NaN	0
OSD105	ERR771100	69.023323	4.12	Cambridge Bay, Nunavut,	Northwestern Passages	-0.72	NaN	NaN	NaN	7116
OSD106	ERR771098	65.9449	15	REYKIS	North Atlantic Ocean	7.5	NaN	NaN	NaN	1080
OSD106	ERR771099	65.9449	0	REYKIS	North Atlantic Ocean	7.6	NaN	NaN	NaN	1080
OSD107	ERR771097	39.14039	0	Lisboa	North Atlantic Ocean	20.2	NaN	NaN	NaN	243
OSD108	ERR771096	38.757283	0	Alcochete	North Atlantic Ocean	20.1	NaN	NaN	NaN	508
OSD109	ERR771095	38.676942	0	Rosário	North Atlantic Ocean	20.5	NaN	NaN	NaN	33
OSD110	ERR771094	40.145122	0	Figueira da Foz	North Atlantic Ocean	23	NaN	NaN	NaN	618
OSD111	ERR771093	40.659875	0	Ria de Aveiro_1	North Atlantic Ocean	25.2	NaN	NaN	NaN	238
OSD113	ERR771092	38.6667	39	CascaisWatch	North Atlantic Ocean	18	0.65	1.4225	NaN	2464
OSD115	ERR771090	39.134347	0	Santa Cruz	North Atlantic Ocean	20.3	NaN	NaN	NaN	309
OSD116	ERR771089	39.415067	0	Lagoa de Óbidos	North Atlantic Ocean	24.7	NaN	NaN	NaN	613
OSD117	ERR771088	37.167	0	S Iberian Atlantic - Tavira Beach	North Atlantic Ocean	23.64	NaN	NaN	NaN	0
OSD118	ERR771087	51.7423	0.2	Robert's Cove	Celtic Sea	18	5.2647	0.8065	NaN	70
OSD122	ERR771086	29.4667	0	Station A Gulf Of Eilat	Gulf of Aqaba	24	NaN	0.25	NaN	3703
OSD124	ERR771084	34.32444	5	Osaka Bay	Japan Sea	21.15	NaN	NaN	NaN	422
OSD125	ERR771083	55.03306	0	Dove Marine Laboratory Cullercoats Beach	North Sea	16.04	NaN	NaN	50	0
OSD126	ERR771082	66.00691	1	Eyafjordur_1	Greenland Sea	12.2	NaN	NaN	NaN	0
OSD127	ERR771081	66.00776	10	Eyafjordur_2	Greenland Sea	11.24	NaN	NaN	NaN	0
OSD128	ERR771080	65.4886	1	Eyafjordur_3	Greenland Sea	12	NaN	NaN	NaN	0
OSD129	ERR771079	65.817186	10	Eyafjordur_4	Greenland Sea	9.9	NaN	NaN	NaN	1325
OSD13	ERR771078	43.185	0	Varna Bay	Black Sea	22.87	NaN	NaN	NaN	779
OSD130	ERR771077	66.1316	1	Eyafjordur_5	Greenland Sea	10.1	NaN	NaN	NaN	170
OSD131	ERR771076	42.4138056	1	Sozopol bay (Zlatana ribka)	Black Sea	22.5	NaN	NaN	NaN	0
OSD132	ERR771075	32.0694	0.5	Sdot YAM	Mediterranean Sea - Eastern Basin	27.3	NaN	1.57	NaN	0
OSD133	ERR771074	-33.897069	0	Robben Island	South Atlantic Ocean	15.06	NaN	NaN	95.87	312
OSD14	ERR771073	42.49	2	Banyuls	Mediterranean Sea - Western Basin	20.422	0.01	0.02	224.06	523
OSD141	ERR771072	60.16121	5	Raunefjorden	North Sea	10.13	0.2	NaN	NaN	1557
OSD143	ERR771070	31.98282	1	Skidaway Institute of Oceanography	North Atlantic Ocean	30.79	0.7	0.1	5.85	0
OSD144	ERR771069	21.26882	0	Maunalua Bay O'ahu	North Pacific Ocean	25.8	NaN	NaN	NaN	708
OSD145	ERR771068	51.361369	0	North Sea - Blankenberge	North Sea	17	NaN	NaN	NaN	4528
OSD147	ERR771066	8.873739	0	Rajarata	Bay of Bengal	28.8	NaN	NaN	229.2	1103
OSD148	ERR771065	53.580926	0	Wadden Sea	North Sea	17.77	NaN	NaN	9.1	254
OSD149	ERR771064	-34.616	0	Laguna Rocha Norte	South Atlantic Ocean	10.98	NaN	3.8	NaN	0
OSD15	ERR771062	43.6861111	50	Villefranche - SOMLIT	Mediterranean Sea - Western Basin	16.0522	NaN	NaN	5.43573	529
OSD15	ERR771063	43.6861111	0	Villefranche - SOMLIT	Mediterranean Sea - Western Basin	21.778	NaN	NaN	4.84812	529
OSD150	ERR771061	-34.666	0	Laguna Rocha Sur	South Atlantic Ocean	9.99	NaN	3.5	NaN	0
OSD151	ERR771060	-34.7	0	South Atlantic Microbial Observatory	South Atlantic Ocean	11.67	NaN	21.7	15.6	0
OSD152	ERR771058	44.6936	5	Compass Buoy Station - Bedford Basin	North Atlantic Ocean	10.8	NaN	NaN	304	0
OSD152	ERR771059	44.6936	1	Compass Buoy Station - Bedford Basin	North Atlantic Ocean	12.8	NaN	NaN	294	0
OSD153	ERR771057	36.997655	0	Faro Island	North Atlantic Ocean	21.1	NaN	NaN	8.83	0
OSD154	ERR771056	44.66666	1	Arcachon/Eyrac-SOMLIT	Bay of Biscay	20.7	0.04	1	231.4	260
OSD155	ERR771055	59.81618	1	Steilene Oslofjord	Skaggerak	18.7	NaN	NaN	NaN	1278
OSD156	ERR771054	59.89961	1	KAVRINGEN ligthouse	Skaggerak	17.8	NaN	NaN	NaN	769
OSD157	ERR771053	59.622	1	Drøbak, Oslofjorden (ELLEIm2)	Skaggerak	18	NaN	NaN	NaN	469
OSD159	ERR771051	48.359	2	Brest-SOMLIT	Celtic Sea	16	0.034	0.274	239.48	462
OSD162	ERR771050	56.9631	1	Stonehaven	North Sea	12.2	NaN	NaN	NaN	5358
OSD163	ERR771049	58.957	2	Scapa	North Sea	11.7	NaN	NaN	NaN	81
OSD165	ERR771048	57.8498	2	Loch Ewe	Inner Seas off the West Coast of Scotland	14.3	NaN	NaN	NaN	508
OSD166	ERR771047	43.43287	0.5	Armintza	Bay of Biscay	17.88	2.79	95.15	NaN	0
OSD167	ERR771046	65.7064	0.1	Eyafjordur_6	Greenland Sea	11	NaN	NaN	NaN	100
OSD168	ERR771045	38.41333	2	IMST_izmir	Aegean Sea	25.7352	4.563	0.123	268.671	0
OSD169	ERR771044	51.796139	0.1	Brightlingsea Reach, Essex	North Sea	18.5	1.426	3.842	NaN	480
OSD170	ERR771042	51.269517	3	130	North Sea	18.6188	NaN	NaN	NaN	3489
OSD171	ERR771041	51.307333	3	230	North Sea	18.2617	NaN	NaN	NaN	9015
OSD172	ERR771040	51.37485	3	700	North Sea	18.4817	NaN	NaN	NaN	3038
OSD177	ERR771035	51.18575	3	120	North Sea	18.7362	NaN	NaN	NaN	3717
OSD18	ERR771033	35.363732	75	Kyrenia	Mediterranean Sea - Eastern Basin	20.7	0.03	0.2	8.02	2087
OSD186	ERR771028	38.885507	2.4	SERC Rhode River Maryland	North Atlantic Ocean	26.8	NaN	NaN	6.2	456
OSD19	ERR771027	35	2	Famagusta	Mediterranean Sea - Eastern Basin	29	NaN	NaN	NaN	0
OSD2	ERR771026	48.7778	0	Roscoff - SOMLIT	English Channel	14.38	0.1	1.3	282.7	5401
OSD20	ERR771024	64.208333	0	Faxafloi	North Atlantic Ocean	11	NaN	NaN	NaN	5090
OSD20	ERR771025	64.208333	20	Faxafloi	North Atlantic Ocean	11	NaN	NaN	NaN	5090
OSD21	ERR771023	45.08	0	Croatia	Adriatic Sea	19.5	NaN	NaN	NaN	1559

OSD24	ERR771021	35.1927	0	Marchica	Alboran Sea	26.5	0.25	12.5	7.78	2362
OSD25	ERR771020	35.086353	0	Saidia Rocher	Alboran Sea	23.1	0.16	0.67	7	0
OSD26	ERR771019	35.82	0	Tangier	Strait of Gibraltar	28	NaN	NaN	NaN	216
OSD28	ERR771018	16.802575	0.2	Belize	Caribbean Sea	29.5	NaN	NaN	NaN	2802
OSD29	ERR771017	27.4694	0.2	Florida	North Atlantic Ocean	26.9	NaN	NaN	5.6	524
OSD3	ERR771016	54.18194	0	Helgoland	North Sea	14	0.1	2.86	NaN	0
OSD30	ERR771015	59.8822	2	Tvärminne	Gulf of Finland	10.5	0.2	NaN	10.46	1297
OSD34	ERR771014	31.21667	0	Alexandria	Mediterranean Sea - Eastern Basin	27	NaN	NaN	NaN	0
OSD35	ERR771013	38.6792	1	Cheasapeake Bay	North Atlantic Ocean	26.32	NaN	NaN	260.37	0
OSD36	ERR771012	39.3322	0.5	Delaware	North Atlantic Ocean	23.55	NaN	NaN	392.86	454
OSD37	ERR771011	26.10293	1	Port Everglades	North Atlantic Ocean	27.7	NaN	NaN	NaN	1069
OSD38	ERR771010	24.7449	0	Long Key	North Atlantic Ocean	29.6	NaN	NaN	NaN	6942
OSD39	ERR771009	32.7524	0.1	Charleston Harbor	North Atlantic Ocean	31.3	NaN	NaN	5.27	42
OSD4	ERR771008	40.808	0	LTER-MC	Tyrrhenian Sea	23.3	0.24	0.59	200.59	2034
OSD41	ERR771007	48.04051	1	Sequim Bay Park	The Coastal Waters of Southeast Alaska and British Columbia	15.9	0.931	0.3	0.01	155
OSD42	ERR771006	38.26861	3	Faro Lake	Tyrrhenian Sea	20	NaN	NaN	NaN	0
OSD43	ERR771005	32.86698	0	SIO Pier	North Pacific Ocean	20.04	0.18	0.07	257.73	402
OSD45	ERR771004	27.61578	0	Tampa Bay	Gulf of Mexico	31.2	NaN	NaN	NaN	130
OSD46	ERR771003	30.2484	0	Horn Island	Gulf of Mexico	29.8	NaN	NaN	187	611
OSD47	ERR771002	45.502	0	Venice Lagoon	Adriatic Sea	25.3	0.165	1.75	4.26	253
OSD48	ERR771001	45.4125	0	Venice Gulf	Adriatic Sea	22.2	0.09	4.89	7.31	5262
OSD50	ERR770997	43.333333	0	Pasaia	Bay of Biscay	20	0.16	1.6	NaN	0
OSD51	ERR770996	9.3485	2	Bocas del Toro	Caribbean Sea	29.1	NaN	NaN	1866.66	97
OSD52	ERR770995	27.02527	1	Abu Hashish	Red Sea	27	NaN	NaN	191.1249	1327
OSD53	ERR770994	27.041533	1	Ras Disha	Red Sea	27.28333333	NaN	NaN	204.6354167	782
OSD54	ERR770993	43.8444	1	Maine Booth Bay	North Atlantic Ocean	11.9	NaN	NaN	NaN	189
OSD55	ERR770992	43.8604	1	Maine Damariscotta River	North Atlantic Ocean	12.5	NaN	NaN	NaN	0
OSD57	ERR770990	21.28656	0	Hawaii Oahu	North Pacific Ocean	27.58	NaN	NaN	NaN	0
OSD58	ERR770989	34.7181	1	PICO	North Atlantic Ocean	25.8	NaN	NaN	NaN	0
OSD6	ERR770988	41.6666	0	Blanes	Balearic Sea	20.66	NaN	NaN	NaN	932
OSD60	ERR770987	33.32306	0.1	South Carolina 2 - North Inlet	North Atlantic Ocean	27.77	NaN	NaN	5.51	90
OSD61	ERR770986	41.524467	0.5	Vineyard Sound	North Atlantic Ocean	19.2	NaN	NaN	7.38	0
OSD62	ERR770985	53.225417	0.2	Manai Straits	Irish Sea and St. George's Channel	16	NaN	NaN	NaN	0
OSD64	ERR770983	46.44155	1	Odessa	Black Sea	20.3	1	1	NaN	145
OSD65	ERR770982	-36.292794	0.1	Leigh Marine Laboratory (NZ)	South Pacific Ocean	16	NaN	1.1	NaN	575
OSD69	ERR770981	45.4568	0	Marghera	Adriatic Sea	25.7	0.24	7.69	5.01	0
OSD70	ERR770979	45.4142	0	Lido	Adriatic Sea	23.4	0.145	3.81	5.47	1547
OSD71	ERR770978	-45.7442	0	Otago	South Pacific Ocean	10.99	NaN	NaN	9.05	4621
OSD72	ERR770977	54.8333	0.8	Boknis Eck	Kattegat	13.994	0.03	0.03	9.426	2504
OSD73	ERR770976	41.6835	1	Lima Estuary	North Atlantic Ocean	18.4	3.52	12.16	8.03	315
OSD74	ERR770975	41.1416	1	Douro Estuary	North Atlantic Ocean	20.2	3.96	58.92	9.08	387
OSD76	ERR770974	43.9475	0.5	Foglia	Adriatic Sea	23.61	0.31	4.8	NaN	2898
OSD77	ERR770973	43.8514	0.5	Metauro	Adriatic Sea	24.1	0.6	7.4	NaN	2079
OSD78	ERR770972	43.57	0.5	CONISMA	Adriatic Sea	24.25	NaN	NaN	NaN	247
OSD80	ERR770970	74.31	2	Young Sound	Greenland Sea	-1.6	NaN	NaN	NaN	1666
OSD80	ERR770971	74.31	0	Young Sound	Greenland Sea	-0.1	NaN	NaN	NaN	1666
OSD81	ERR770969	37.005053	0	Ria Formosa Lagoon	North Atlantic Ocean	22.2	NaN	NaN	7.86	407
OSD90	ERR770967	38.48435	2	Etoliko Lagoon	Ionian Sea	26.29	0.0116	0.016	NaN	1083
OSD91	ERR770966	32.74675	2	Oualidiya	North Atlantic Ocean	19	NaN	107.93	0.7	0
OSD92	ERR770965	33.583917	2	Casablanca	North Atlantic Ocean	24	NaN	105.01	0.78	0
OSD93	ERR770964	33.259611	2	Eljadida	North Atlantic Ocean	19	NaN	111.76	0.78	49
OSD94	ERR770963	35.086353	0	Saidia Marina	Alboran Sea	23.6	0.27	0.15	7	0
OSD95	ERR770962	1.2685	0	Singapore Indigo V	Singapore Strait	31	NaN	NaN	171	3040
OSD97	ERR770960	38.5297	0.5	Faial Azores	North Atlantic Ocean	16.9	NaN	NaN	NaN	1623
OSD98	ERR770959	38.64	0.5	Sao Jorge Azores	North Atlantic Ocean	18.7	NaN	NaN	NaN	179
OSD99	ERR770958	45.70092	1	C1	Adriatic Sea	20.821	0.012	2.321	208.31	360

Supplementary Methods

Reconstructing Rinke et al. (2018) phylogenetic tree

The 270 genomes used in Rinke *et al.* (2018)[1] were accessed based on NCBI accession IDs provided in Supplementary Table and Figures document provided as a supplemental information for that manuscript. As in the methodology section, putative coding DNA sequence (CDS) were predicted for each using Prodigal[2] (v.2.6.3). The predicted proteins sequences for each genome were searched (HMMER[3] v.3.1b2; hmmsearch -E 1E-5) using HMM models representing the 120 single copy marker proteins as detailed by the Genome Taxonomy Database (GTDB v.86.0; http://gtdb.ecogenomic.org/) and sourced from TIGRfam[4] (v.15) and Pfam[5] (v.31.0) (Supplemental Data 3). A total of 110 markers were detected in the genomes as a single copy. Individual markers genes were aligned using MUSCLE[6] (v.3.8.31; -maxiters 8) and automatically trimmed using trimAL[7] (v.1.2rev59; -automated1). No minimum number of markers was applied

to filter the dataset. Proteins alignments were concatenated and a phylogenomic tree was constructed using FastTreeDbl[8] (v.2.1.10; -gamma -lg).

Manual bin refinement

As detailed in the Methods section, several genomes originating from Tully *et al.* (2017, 2018)[9,10] were identified that had elevated estimated genome contamination levels (~5% or greater). To access the data of these refinement steps for the five genomes present in the analyzed genome dataset, the Anvi'o[11] contigs (contigs.db) and profile (PROFILE.db) have been provided (figshare). The (iterative) manual binning results can be accessed using the Anvi'o command anvi-interactive (-p PROFILE.db -c contigs.db). Contigs related to the TOBG genomes MED853 and MED921 and the TMED genome TMED132 were combined with overlapping bins generated by CONCOCT[12] and used to recruit the 20 *Tara* Oceans metagenomes originating from the Mediterranean Sea. The same approach was used for TOBG genomes SAT1485 and SP3063, except that reads were recruited only from the bacterial and girus fractions originating from the corresponding 25 South Atlantic and 24 South Pacific metagenomes, respectively.

Generally for each genome and profile pair, there are three bin collections that can be overlaid on the contig recruitment data: 'CONCOCT' presents predicted bins as determined by CONCOCT; 'Stage1' presents the contigs identified in the 'original' genome; and, 'Version1' presents the contigs in the 'refined' genome and the contigs that remain in the 'original'. 'Refined' contigs were selected based on the location of the majority of contigs in the original genome and contigs were removed that are not within that cluster. Contigs were added to the genome if they clustered on the hierarchical dendrogram with the majority of contigs and had complementary recruitment patterns and percent GC-content. Refined genomes were assessed using CheckM for estimated completeness and contamination. Refined genomes that did not see an improvement in completion or contamination were further refined, as is the case with SP3063. SP3063 has two bin collections, 'Version1' and 'Version2', where 'Version1' represents the first manual refinement attempt ('attempt1') and 'Version2' represents the 'refined' genome.

Supplementary Discussion

On the Rinke *et al.* (2018)[1] phylogenetic tree, the genera J1-3 and Q1-2 were represented by 1-2 genomes each. Two additional genera level groups were missed by Rinke *et al.* due to the lack of inclusion of MGII genomes from Thrash *et al.* (2017)[13]. Both MGIIBin17 and MGIIBin15 may represent unique subclades, distinct from proposed clades J and Q. Subclade MGIIa.2 and genus K2 likely represent the same phylogenetic group, but have no overlapping genomes. Future phylogenetic trees combining the new genomes from Rinke *et al.* should alleviate this discrepancy. The single representative of genus L4 (UBA15) was present on the phylogenetic tree constructed for all redundant genomes for this manuscript. UBA15, detected by Rinke *et al.* in the Northwest Arabian Sea, had ≥98.5% average nucleotide identity (ANI) to the genome SP339, reconstructed from the South Pacific, This expands the range of that genus beyond the Arabian Sea. Discrepancies between MGIIa.7 and MGIIa.8/genus L1 and MGIIb.12 and MGIIb.13/genus O3 could be resolved by slight modifications in the cutoff value for relative evolutionary distance applied to each genus. This change that would likely maintain the rank normalization groupings presented in Figure S5 in Rinke *et al.*

Supplementary References

1. Rinke, C. *et al.* A phylogenomic and ecological analysis of the globally abundant Marine Group II archaea (Ca. Poseidoniales ord. nov.). *ISME J* **89,** 1–13 (2018).

2. Hyatt, D., LoCascio, P. F., Hauser, L. J. & Uberbacher, E. C. Gene and translation initiation site prediction in metagenomic sequences. *Bioinformatics* **28,** 2223–2230 (2012).
3. Finn, R. D., Clements, J. & Eddy, S. R. HMMER web server: interactive sequence similarity searching. *Nucleic Acids Res.* **39,** W29–W37 (2011).
4. Haft, D. H., Selengut, J. D. & White, O. The TIGRFAMs database of protein families. *Nucleic Acids Res.* **31,** 371–373 (2003).
5. Bateman, A. *et al.* The Pfam Protein Families Database. *Nucleic Acids Res.* **30,** 276–280 (2002).
6. Edgar, R. C. MUSCLE: multiple sequence alignment with high accuracy and high throughput. *Nucleic Acids Res.* **32,** 1792–1797 (2004).
7. Capella-Gutiérrez, S., Silla-Martínez, J. M. & Gabaldón, T. trimAl: a tool for automated alignment trimming in large-scale phylogenetic analyses. *Bioinformatics* **25,** 1972–1973 (2009).
8. Price, M. N., Dehal, P. S. & Arkin, A. P. FastTree 2--approximately maximum-likelihood trees for large alignments. *PLoS ONE* **5,** e9490 (2010).
9. Tully, B. J., Sachdeva, R., Graham, E. D. & Heidelberg, J. F. 290 metagenome-assembled genomes from the Mediterranean Sea: a resource for marine microbiology. *PeerJ* **5,** e3558–15 (2017).
10. Tully, B. J., Graham, E. D. & Heidelberg, J. F. The Reconstruction of 2,631 Draft Metagenome-Assembled Genomes from the Global Oceans. *bioRxiv* 1–10 (2017). doi:10.1101/162503
11. Eren, A. M. *et al.* Anvi'o: an advanced analysis and visualization platform for 'omics data. *PeerJ* **3,** e1319 (2015).
12. Alneberg, J. *et al.* Binning metagenomic contigs by coverage and composition. *Nat Meth* **11,** 1144–1146 (2014).
13. Thrash, J. C. *et al.* Metabolic Roles of Uncultivated Bacterioplankton Lineages in the Northern Gulf of Mexico 'Dead Zone'. *mBio* **8,** e01017–17–20 (2017).
