# Supplementary material for: Metabolic diversity within the globally abundant Marine Group II Euryarchaea offers insight into ecological patterns
Source: Nat Commun. 2019 Jan 17;10:271. doi: 10.1038/s41467-018-07840-4 (PMC6336850; doi:10.1038/s41467-018-07840-4)
Supplement: Supplementary file 3 — Description of Additional Supplementary Files [file 41467_2018_7840_MOESM3_ESM.pdf]

Description of Additional Supplementary Files

File Name: Supplementary Data 1
Description: Groups of MGII genomes with ≥98.5% ANI denoting representative and duplicate genome IDs.

File Name: Supplementary Data 2
Description: Spreadsheet of the pairwise ANI and AAI values for MGIIa and MGIIb.

File Name: Supplementary Data 3
Description: Counts of the number of proteins identified as an extracellular peptidase or carbohydrateactive enzyme for each genome

File Name: Supplementary Data 4
Description: Newick file of the phylogenomic tree generated using 120 concatenated marker proteins for the full redundant set of MGII genomes with ≥60 markers. Corresponds to Supplementary Figure 1.

File Name: Supplementary Data 5
Description: Newick file of the phylogenomic tree generated using 120 concatenated marker proteins for the analyzed set of MGII genomes. Corresponds to Figure 1.

File Name: Supplementary Data 6
Description: FASTA format of the 16S rRNA gene sequences present in the MGII genomes.

File Name: Supplementary Data 7
Description: Newick file of the phylogenetic tree generated using the 16S rRNA gene sequences present in the MGII genomes. Corresponds to Supplementary Figure 4.

File Name: Supplementary Data 8
Description: FASTA format of the putative proteorhodopsin sequences identified in the MGII genomes

File Name: Supplementary Data 9
Description: Newick file of the phylogenetic tree generated using the proteorhodopsin sequences present in the MGII genomes. Corresponds to Supplementary Figure 6.

File Name: Supplementary Data 10
Description: Protein clusters generated using the Anvi'o pangenome workflow identified as either 'Core MGII', 'Core MGIIa', or 'Core MGIIb'. Only includes proteins with putative KEGG assignments.

File Name: Supplementary Data 11
Description: Derived relative fraction and RPKM values for all MGII genomes for all Tara Oceans and Ocean Sampling Day samples. Corresponds to Figure 5 and Supplementary Figure 8.

File Name: Supplementary Data 12
Description: Spreadsheet of the PERMANOVA statistics (F statistic, p-value, and p-value BenjaminiHochberg False Discovery Rate) and the results of the ad hoc pairwise PERMANOVA tests (p-value Bonferroni corrected).
