# Supplementary material for: Metabolic diversity within the globally abundant Marine Group II Euryarchaea offers insight into ecological patterns
Source: Nat Commun. 2019 Jan 17;10:271. doi: 10.1038/s41467-018-07840-4 (PMC6336850; doi:10.1038/s41467-018-07840-4)
Supplement: Supplementary file 16 — Reporting Summary [file 41467_2018_7840_MOESM16_ESM.pdf]

nature research

Corresponding author(s): Benjamin J Tully

Reporting Summary

Nature Research wishes to improve the reproducibility of the work that we publish. This form provides structure for consistency and transparency in reporting. For further information on Nature Research policies, see Authors & Referees and the Editorial Policy Checklist.

Statistical parameters

When statistical analyses are reported, confirm that the following items are present in the relevant location (e.g. figure legend, table legend, main text, or Methods section).

n/a	Confirmed	
☐	☒	The exact sample size (n) for each experimental group/condition, given as a discrete number and unit of measurement
☐	☒	An indication of whether measurements were taken from distinct samples or whether the same sample was measured repeatedly
☐	☒	The statistical test(s) used AND whether they are one- or two-sided<br>*Only common tests should be described solely by name; describe more complex techniques in the Methods section.*
☐	☒	A description of all covariates tested
☐	☒	A description of any assumptions or corrections, such as tests of normality and adjustment for multiple comparisons
☐	☒	A full description of the statistics including central tendency (e.g. means) or other basic estimates (e.g. regression coefficient) AND variation (e.g. standard deviation) or associated estimates of uncertainty (e.g. confidence intervals)
☐	☒	For null hypothesis testing, the test statistic (e.g. F, t, r) with confidence intervals, effect sizes, degrees of freedom and P value noted<br>*Give P values as exact values whenever suitable.*
☒	☐	For Bayesian analysis, information on the choice of priors and Markov chain Monte Carlo settings
☒	☐	For hierarchical and complex designs, identification of the appropriate level for tests and full reporting of outcomes
☒	☐	Estimates of effect sizes (e.g. Cohen's d, Pearson's r), indicating how they were calculated
☐	☒	Clearly defined error bars<br>*State explicitly what error bars represent (e.g. SD, SE, CI)*

Our web collection on statistics for biologists may be useful.

Software and code

Policy information about availability of computer code

Data collection	n/a
Data analysis	*Provide a description of all commercial, open source and custom code used to analyse the data in this study, specifying the version used OR state that no software was used.*

For manuscripts utilizing custom algorithms or software that are central to the research but not yet described in published literature, software must be made available to editors/reviewers upon request. We strongly encourage code deposition in a community repository (e.g. GitHub). See the Nature Research guidelines for submitting code & software for further information.

Data

Policy information about availability of data

All manuscripts must include a data availability statement. This statement should provide the following information, where applicable:

- Accession codes, unique identifiers, or web links for publicly available datasets
- A list of figures that have associated raw data
- A description of any restrictions on data availability

The genomes used in this study are publicly available, except for a subset of deep-sea MGII from Guaymas basin presented in Li et al. (2015) which were provided by personal communication, and reference IDs are available in Supplemental Table 1. The contigs and proteins used in this study are also available through figshare

(10.6084/m9.figshare.7154813.v1). Genomes from Tully et al. (2017, 2018) that were manually refined have been updated in NCBI with the corresponding accession IDs: NZKR02000000, NZKQ02000000, NZJY02000000, PAEM02000000, PADP02000000, PAUS02000000, PAMN02000000, PBGP02000000, PBGL02000000, NHGH02000000. All supplemental data is available through figshare (10.6084/m9.figshare.7154813.v1). A reporting summary for this Article is available as a Supplementary Information file. The source data underlying Figures 1, 2a-d, 3, 4, 5 and Supplemental Figures 1, 2, 3, 4, 6, and 8 are provided as a Source Data file.

Field-specific reporting

Please select the best fit for your research. If you are not sure, read the appropriate sections before making your selection.

☒ Life sciences ☐ Behavioural & social sciences ☐ Ecological, evolutionary & environmental sciences

For a reference copy of the document with all sections, see nature.com/authors/policies/ReportingSummary-flat.pdf

Life sciences study design

All studies must disclose on these points even when the disclosure is negative.

Sample size	410 genomes assigned to the Marine Group II Euryarchaea were collected from NCBI GenBank prior to Jan 1, 2018
Data exclusions	Identical genomes were identified based on an average nucleotide identity cutoff of 98.5% and a single representative was selected. 8 additional genomes had an insufficient number of phylogenetic markers form placement of the phylogenetic tree are were excluded from the dataset.
Replication	n/a
Randomization	n/a
Blinding	n/a

Reporting for specific materials, systems and methods

Materials & experimental systems

n/a	Involved in the study	
☒	☐	Unique biological materials
☒	☐	Antibodies
☒	☐	Eukaryotic cell lines
☒	☐	Palaeontology
☒	☐	Animals and other organisms
☒	☐	Human research participants

Methods

n/a	Involved in the study	
☒	☐	ChIP-seq
☒	☐	Flow cytometry
☒	☐	MRI-based neuroimaging
